# Diversity and scale: Genetic architecture of 2068 traits in the VA Million Veteran Program

**DOI:** 10.1126/science.adj1182

**Published:** 2024-07-19

**Authors:** Anurag Verma, Jennifer E. Huffman, Alex Rodriguez, Mitchell Conery, Molei Liu, Yuk-Lam Ho, Youngdae Kim, David A. Heise, Lindsay Guare, Vidul Ayakulangara Panickan, Helene Garcon, Franciel Linares, Lauren Costa, Ian Goethert, Ryan Tipton, Jacqueline Honerlaw, Laura Davies, Stacey Whitbourne, Jeremy Cohen, Daniel C. Posner, Rahul Sangar, Michael Murray, Xuan Wang, Daniel R. Dochtermann, Poornima Devineni, Yunling Shi, Tarak Nath Nandi, Themistocles L. Assimes, Charles A. Brunette, Robert J. Carroll, Royce Clifford, Scott Duvall, Joel Gelernter, Adriana Hung, Sudha K. Iyengar, Jacob Joseph, Rachel Kember, Henry Kranzler, Colleen M. Kripke, Daniel Levey, Shiuh-Wen Luoh, Victoria C. Merritt, Cassie Overstreet, Joseph D. Deak, Struan F. A. Grant, Renato Polimanti, Panos Roussos, Gabrielle Shakt, Yan V. Sun, Noah Tsao, Sanan Venkatesh, Georgios Voloudakis, Amy Justice, Edmon Begoli, Rachel Ramoni, Georgia Tourassi, Saiju Pyarajan, Philip Tsao, Christopher J. O’Donnell, Sumitra Muralidhar, Jennifer Moser, Juan P. Casas, Alexander G. Bick, Wei Zhou, Tianxi Cai, Benjamin F. Voight, Kelly Cho, J. Michael Gaziano, Ravi K. Madduri, Scott Damrauer, Katherine P. Liao

**Affiliations:** 1 Corporal Michael Crescenz VA Medical Center, Philadelphia, PA 19104, USA; 2 Department of Medicine, Division of Translational Medicine and Human Genetics, University of Pennsylvania - Perelman School of Medicine, Philadelphia, PA 19104, USA; 3 Institute for Biomedical Informatics, University of Pennsylvania - Perelman School of Medicine, Philadelphia, PA 19104, USA; 4 Massachusetts Veterans Epidemiology Research and Information Center (MAVERIC), VA Boston Healthcare System, Boston, MA 02130, USA; 5 Palo Alto Veterans Institute for Research (PAVIR), Palo Alto Health Care System, Palo Alto, CA 94304, USA; 6 Department of Medicine, Harvard Medical School, Boston, MA 02115, USA; 7 Data Science and Learning, Argonne National Laboratory, Lemont, IL 60439, USA; 8 Department of Systems Pharmacology and Translational Therapeutics, University of Pennsylvania - Perelman School of Medicine, Philadelphia, PA 19104, USA; 9 Department of Biostatistics, Columbia University’s Mailman School of Public Health, New York, NY 10032, USA; 10 Mathematics and Computer Science Division, Argonne National Laboratory, Lemont, IL 60439, USA; 11 National Security Sciences Directorate, Cyber Resilience and Intelligence Division, Oak Ridge National Laboratory, Dept of Energy, Oak Ridge, TN 37831, USA; 12 Department of Biomedical Informatics, Harvard Medical School, Boston, MA 02115, USA; 13 R&D Systems Engineering, Information Technology Services Directorate, Oak Ridge National Laboratory, Dept of Energy, Oak Ridge, TN 37831, USA; 14 MVP Boston Coordinating Center, VA Boston Healthcare System, Boston, MA 02111, USA; 15 Data Management and Engineering, Information Technology Services Division, Oak Ridge National Laboratory, Dept of Energy, Oak Ridge, TN 37831, USA; 16 Knowledge Discovery Infrastructure, Information Technology Services Division, Oak Ridge National Laboratory, Dept of Energy, Oak Ridge, TN 37831, USA; 17 Computing and Computational Sciences Dir PMO, PMO, Oak Ridge National Laboratory, Dept of Energy, Oak Ridge, TN 37831, USA; 18 Department of Medicine, Division of Aging, Brigham and Women’s Hospital, Boston, MA 02115, USA; 19 Department of Population Health Sciences, University of Utah, Salt Lake City, UT 84112, USA; 20 VA Cooperative Studies Program, VA Boston Healthcare System, Boston, MA 02130, USA; 21 Medicine, Cardiology, VA Palo Alto Healthcare System, Palo Alto, CA 94304, USA; 22 Research Service, VA Boston Healthcare System, Boston, MA 02130, USA; 23 Department of Biomedical Informatics, Vanderbilt University Medical Center, Nashville, TN 37211, USA; 24 Research Department, VA San Diego Healthcare System, San Diego, CA 92161, USA; 25 Department of Otolaryngology, UCSD San Diego, La Jolla, CA 92093, USA; 26 VA Informatics and Computing Infrastructure, VA Salt Lake City Health Care System, Salt Lake City, UT 84148, USA; 27 Internal Medicine, Epidemiology, University of Utah School of Medicine, Salt Lake City, UT 84132, USA; 28 Psychiatry, Human Genetics, Yale University, New Haven, CT, 06520, USA; 29 VA Connecticut Healthcare System West Haven, West Haven, CT, 06516, USA; 30 Medicine, Nephrology & Hypertension, VA Tennessee Valley Healthcare System & Vanderbilt University, Nashville, TN 37232, USA; 31 Departments of Population and Quantitative Health Sciences, Genetics and Genome Sciences, and Ophthalmology and Visual Sciences and the Cleveland Institute for Computational Biology, Case Western Reserve University, Cleveland, OH 44106, USA; 32 Medicine, Cardiology Section, VA Providence Healthcare System, Providence, RI 02908, USA; 33 Department of Medicine, Brown University, Providence, RI, 02908, USA; 34 Mental Illness Research, Education and Clinical Center, Corporal Michael Crescenz VA Medical Center, Philadelphia, PA 19104, USA; 35 Department of Psychiatry, University of Pennsylvania - Perelman School of Medicine, Philadelphia, PA 19104, USA; 36 Medicine, VA Connecticut Healthcare System West Haven, West Haven, CT 06516, USA; 37 VA Portland Health Care System, Portland, OR 97239, USA; 38 Division of Hematology and Medical Oncology, Knight Cancer Institute, Oregon Health and Science University, Portland, OR 97239, USA; 39 Psychiatry, Yale University, New Haven, CT 06520, USA; 40 Psychiatry, VA Connecticut Healthcare System West Haven, West Haven, CT 06516, USA; 41 Center for Spatial and Functional Genomics, Children’s Hospital of Philadelphia, Philadelphia, PA 19104, USA; 42 Department of Pediatrics, University of Pennsylvania - Perelman School of Medicine, Philadelphia, PA 19104, USA; 43 Divisions of Human Genetics and Endocrinology and Diabetes, Children’s Hospital of Philadelphia, Philadelphia, PA 19104, USA; 44 Department of Genetics, University of Pennsylvania - Perelman School of Medicine, Philadelphia, PA 19104, USA; 45 Psychiatry, Mental Illness Research, Education and Clinical Center, James J. Peters VA Medical Center; Icahn School of Medicine at Mount Sinai, Bronx, NY 10468, USA; 46 Department of Surgery, University of Pennsylvania - Perelman School of Medicine, Philadelphia, PA 19104, USA; 47 Epidemiology, Emory University Rollins School of Public Health, Atlanta, GA 30322, USA; 48 Internal Medicine, General Medicine, Yale University, New Haven, CT 06520, USA; 49 Health Policy, Yale School of Public Health, New Haven, CT 06520, USA; 50 Oak Ridge National Laboratory, Dept of Energy, Oak Ridge, TN, 37831, USA; 51 Office of Research and Development, Department of Veterans Affairs, Washington, DC, 20420, USA; 52 National Center for Computational Sciences, Oak Ridge National Laboratory, Dept of Energy, Oak Ridge, TN, 37831, USA; 53 Department of Medicine, Stanford University, Palo Alto, CA, 94304, USA; 54 Medicine, Cardiology, VA Boston Healthcare System, Boston, MA 02130, USA; 55 Department of Medicine, Division of Genetic Medicine, Vanderbilt University, Nashville, TN, 37325, USA; 56 Department of Medicine, Analytic and Translational Genetics Unit, Massachusetts General Hospital, Boston, MA 02114, USA; 57 Stanley Center for Psychiatric Research, Cambridge, MA 02142, USA; 58 Program in Medical and Population Genetics, Cambridge, MA 02142, USA; 59 Institute of Translational Medicine and Therapeutics, University of Pennsylvania - Perelman School of Medicine, Philadelphia, PA 19104, USA; 60 Cardiovascular Institute, University of Pennsylvania - Perelman School of Medicine, Philadelphia, PA 19104, USA; 61 Medicine, Rheumatology, VA Boston Healthcare System, Boston, MA 02130, USA; 62 Department of Medicine, Division of Rheumatology, Inflammation, and Immunity, Brigham and Women’s Hospital, Boston, MA 02115, USA

## Abstract

**INTRODUCTION:**

Findings from genome-wide association studies (GWASs) have provided foundational knowledge of the genetic basis of disease, facilitating precision approaches for prevention and treatment. Current GWAS results are limited by underrepresentation of individuals from diverse populations, leading to concerns with generalizability regarding our knowledge of the relationships between genes, traits, and disease. The Department of Veterans Affairs (VA) Million Veteran Program (MVP), one of the largest US-based biobanks, addresses this need; 29% of MVP comprises individuals genetically similar to African (AFR), Admixed American (AMR), and East Asian (EAS) reference populations. With over 635,000 participants and more than 44.3M genotyped variants linked with detailed phenotypic data from the electronic health record (EHR), the MVP has the scale and richness of data to fill in the gaps in our knowledge of genotype-phenotype associations across diverse populations.

**RATIONALE:**

Leveraging dense MVP data, we conducted GWASs across 2068 traits in four population groups based on genetic similarity to AFR, AMR, EAS, and European (EUR) reference populations. We employed statistical fine-mapping to highlight putative causal variants. This effort allowed us to characterize the genetic architecture of complex traits within diverse populations and compare genetic predisposition between population groups. We also quantified the benefits of including individuals from non-EUR population groups in the study for variant discovery and fine-mapping precision. Fine-mapping provided a foundation for nominating putative effector genes at associated loci mapping the landscape of gene-trait associations across populations to highlight both pleiotropic and heterogeneous associations.

**RESULTS:**

Among 635,969 participants, we identified 26,049 variant-trait associations across 1270 traits, with 3477 being significant only when individuals from non-EUR populations were included. Fine-mapping revealed 57,601 independent signals across 936 traits, with 15,045 of these signals mapped with high confidence to a single variant. Predominantly resulting from interpopulation allele frequency differences, 2069 high-confidence signals and 549 gene nominations were unique to non-EUR groups. Notably, a signal mapped to rs76024540 implicated *SLC22A18/SLC22A18AS* as effector genes for keloid scarring, a condition vastly more prevalent in the AFR than the EUR population. Apart from the *APOE* locus’s association with dementia, we observed few instances of effect size heterogeneity across populations for fine-mapped variants.

**CONCLUSION:**

This study underscores the enhanced power of GWASs with increased participant diversity, achieving greater variant discovery and fine-mapping precision than possible in the EUR population alone. Our findings reveal more similarities than differences in genetic architectures across populations, with most differences attributable to allele frequency variations between populations.

## Abstract (summary)

One of the justifiable criticisms of human genetic studies is the underrepresentation of participants from diverse populations. Lack of inclusion must be addressed at-scale to identify causal disease factors and understand the genetic causes of health disparities. We present genome-wide associations for 2068 traits from 635,969 participants in the Department of Veterans Affairs Million Veteran Program, a longitudinal study of diverse United States Veterans. Systematic analysis revealed 13,672 genomic risk loci; 1608 were only significant after including non-European populations. Fine-mapping identified causal variants at 6318 signals across 613 traits. One-third (*n* = 2069) were identified in participants from non-European populations. This reveals a broadly similar genetic architecture across populations, highlights genetic insights gained from underrepresented groups, and presents an extensive atlas of genetic associations.

## Graphical Abstract

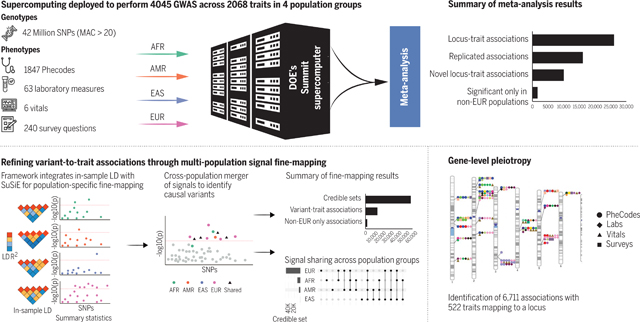

**Comprehensive phenome-wide genetic analysis across multiple populations**. Meta-analysis of 4045 GWASs comprising 2068 traits from four population groups identified 26,049 locus-trait associations, including 9989 previously unreported. Multi-population fine-mapping prioritized high confidence signals, highlighting shared associations and elucidated pleiotropic genes driving multiple variant-trait associations.

Among published genome-wide association studies (GWASs), 95% of participants are genetically similar to individuals from European (EUR) reference populations (1) This creates fundamental inequalities that exacerbate health care disparities as much of our knowledge regarding the relationship between genes, traits, and disease may have limited generalizability to other populations (2)Accordingly, understanding the degree to which the genetics of complex traits are similar remains a fundamental open question in human genetics.

Although steps have been taken to address these discrepancies, there remains a substantial unmet need for large-scale, well-powered analyses across diverse population groups. For example, although several large biobanks have been able to address some of this discrepancy in East Asian populations [China Kadoori (3) and Biobank Japan (4)], aggregated data for individuals genetically similar to African, Admixed American, and Asian reference populations still lack substantial depth. Large-scale sequencing projects have generated valuable resources in characterizing the genetic variation in a wide array of populations; however, they lack the breadth of clinical data common to DNA biobanks with linked electronic health records (EHR), thereby limiting the characterization of the genetic architecture of phenotypes at scale. The Department of Veteran Affairs (VA) Million Veteran Program (MVP), a longitudinal health, genomic, and precision medicine cohort, which was established in 2011 and enrolled its one-millionth Veteran in 2023 (5), has both the population diversity and the genomic and phenotypic depth to address this unmet need. These types of studies will grow as other diverse population-based cohorts, such as NIH’s All of Us program (6), continue to support research and mature.

Characterizing the genetic architecture of complex traits within diverse populations as well as assessing the similarities and differences across populations requires large-scale, population-specific, phenome- and GWASs. Thus, we conducted a set of population-specific, phenome-wide GWASs in 635,969 US Veterans, of whom 29% were genetically similar to African (AFR), Admixed American (AMR), and East Asian (EAS) population groups as determined by similarity to the 1000 Genomes Project reference panel. The results of these GWASs were then used in experiments to compare and contrast the relationship between genetic variation and health and disease traits across these population groups.

Throughout this work, we categorize individuals into groups based on their genetic similarity to individuals sampled from populations across the world. These labels are applied with the understanding that they represent broad, genetically similar groups of people. Although they are not intended to be deterministic of race or ethnic identities they are inextricably intertwined with these social constructs. Our intent in applying categorical population descriptors is to facilitate the study of genetic variation and its association with traits and diseases between diverse populations. We recognize that such categorizations, while necessary for analytical clarity, oversimplify the rich and complex mosaic of human genetic diversity. Nearly all individuals have a component of admixed ancestry, indicative of the blending of genetic lineages from different geographical regions. Therefore, the geographical descriptors applied here are not absolute markers of genetic identity. This approach provides a balance between the need for population-specific genetic insights using the current standardized definitions and the recognition of the continuous nature of human genetic variation.

In what follows, we describe large-scale genomic analysis across diverse populations, resulting in a collection of >13,000 locus-trait associations. Application of fine-mapping techniques enabled the construction of a catalog of putative causal variants across human traits and population groups, which included the identification of signals whereby a single variant is credibly implicated. This core analysis allowed us to interrogate the contribution of signals from non-EUR populations and facilitated a systematic comparison of genetic architecture across population groups, thereby identifying signals, variants, genes, and global genetic architecture that are similar and different between population groups. Findings from this study aim to expand the current knowledge base on population genetic architecture and to underscore the importance of diversity in genetic research in uncovering the full spectrum of human genetic variation and its impact on complex traits.

## Results

### Study design, population groups, and phenotypic definitions

**Fig. 1.**
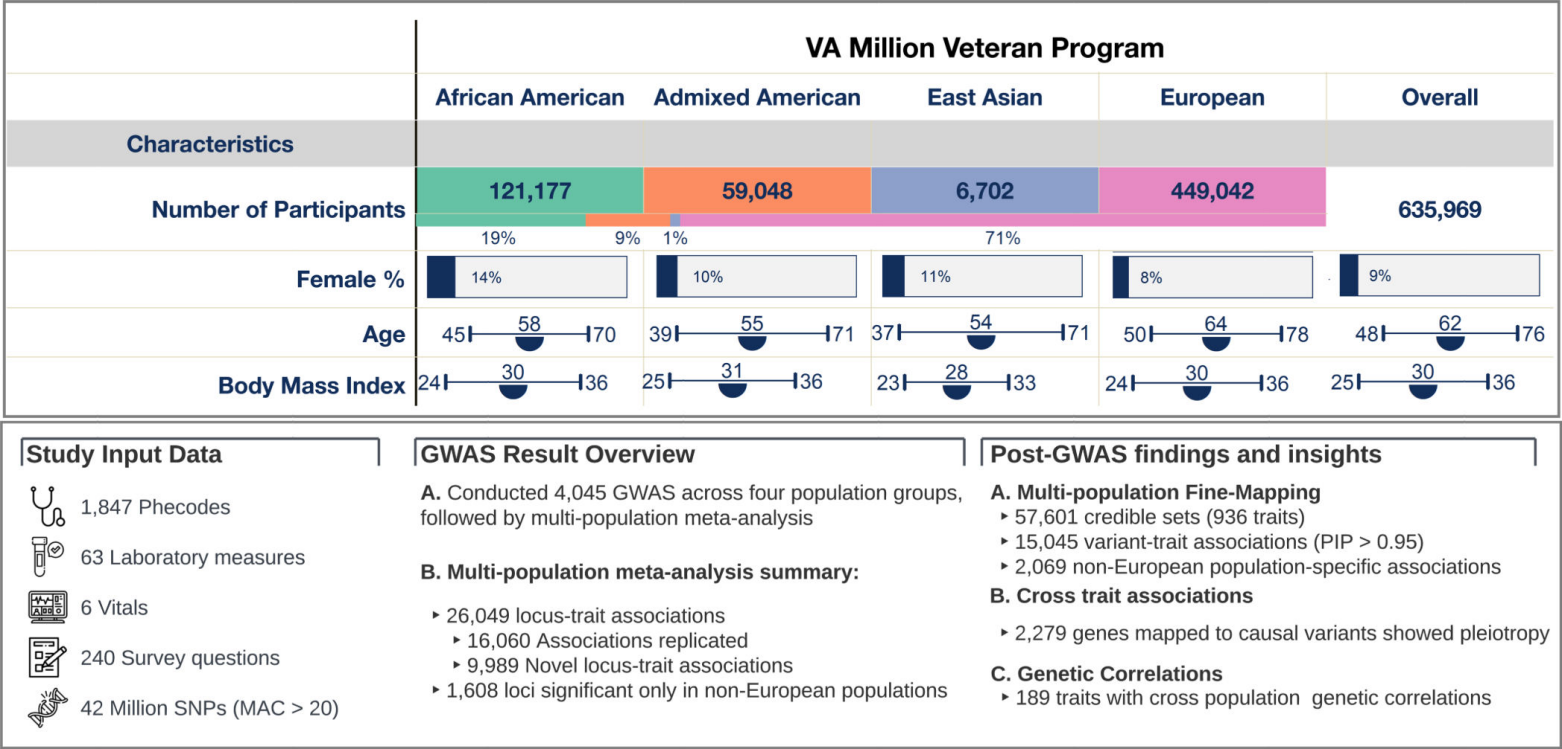
Overview of the study population, genetic association results, and post-GWAS findings. Top panel depicts the demographic characteristics of the study population; semicircles represent the mean values for age and body mass index. Bottom panel is organized into three sections: the left section summarizes the study data, the middle section provides key metrics of GWAS results such as the count of independent loci and lead SNPs, and the right section briefly outlines the post-GWAS findings.

The study analyzed data from 635,969 participants (MVP Genomics Release 4) (7), aggregated into four population groups based on genetic similarity to the 1000 Genomes Project (8) AFR (*n* = 121,177), AMR (*n* = 59,048), EAS (*n* = 6702), and EUR (*n* = 449,042) superpopulations (Fig. 1). The population was 8.8% female according to clinical records, with mean age 61.9 years and mean body mass index (BMI) 30.2 kg/m^2^; 20.6% were current smokers with 68.5% having smoked 100 cigarettes in their lifetime (table S1). After imputation and quality control (QC) filtering, > 44.3M variants [with minor allele count (MAC) > 40] were included for analysis (9). The frequency and imputation quality scores of single nucleotide polymorphisms (SNPs) among the population groups are provided within the GWAS results (see data and materials availability).

**Fig. 2.**
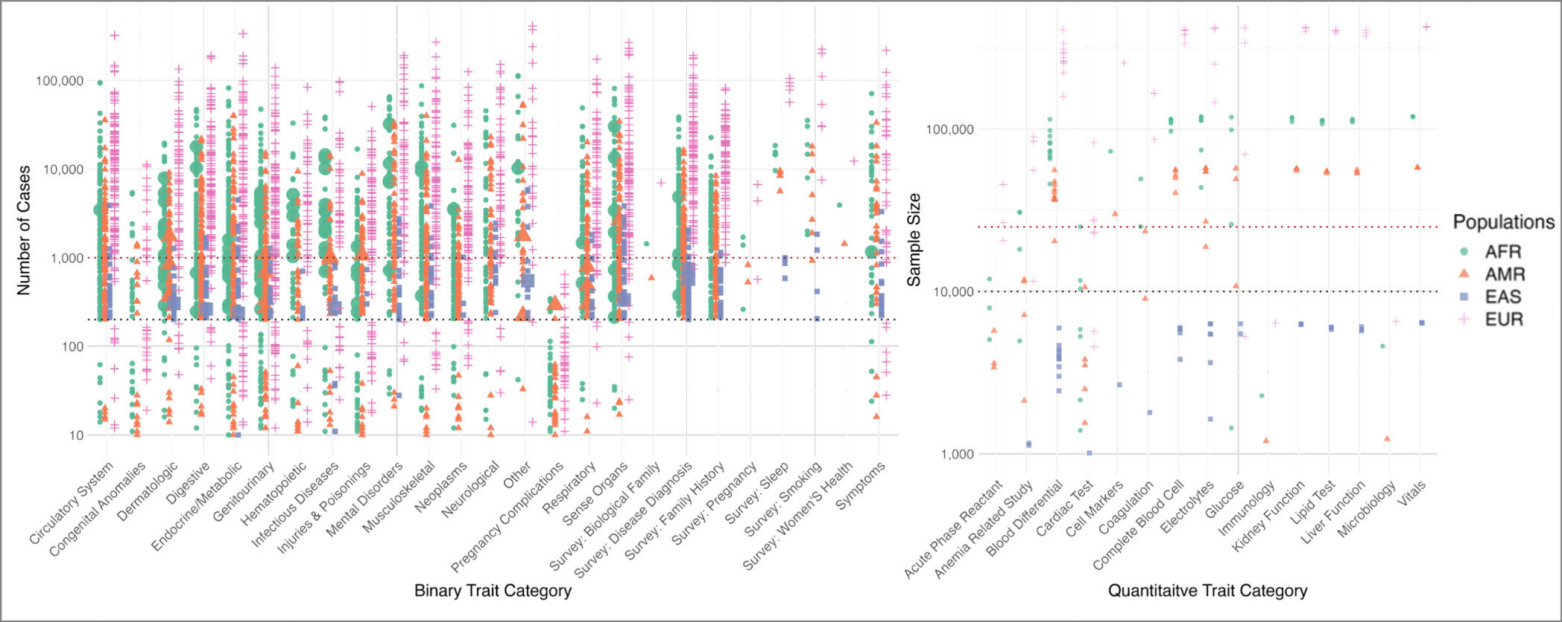
Prevalence and sample sizes of 2078 traits. The left plot illustrates the number of cases (y-axis) across binary trait categories (x-axis), and the right plot presents the sample (y-axis) across quantitative trait categories (x-axis). Population groups are represented by distinct colors and shapes. Larger shapes indicate conditions that are twice as prevalent when compared to EUR (see table S2)

We extracted phenotypic trait data comprising diagnosis codes, laboratory measures, and vital signs from the VA EHR. Additionally, we included responses to survey questions on health and behavior administered at MVP enrollment. After QC, 1854 binary and 214 quantitative traits were included in the downstream analysis in at least one population group (*n* = 2068, Fig. 1) (9). Several traits had increased prevalence in non-EUR groups compared to the EUR group (Fig. 2, table S2), highlighting the importance of including diverse populations in genetic studies. Within the AFR group, 101 traits (6.3%) exhibited a prevalence at least twice as high as that observed in the EUR group, notably including traits such as hereditary hemolytic anemias, sarcoidosis, and keloid scarring. While the sample sizes for the AMR and EAS groups were relatively smaller, there were 18 traits in AMR and 8 traits in EAS with at least twice the prevalence of EUR. Among these traits, alopecia areata in AMR and viral hepatitis B in EAS had notably higher prevalence.

### Biobank-scale genomic analysis across populations identifies tens of thousands of variant-trait associations

We next turned to the substantial computational task of calculating the >350 billion variant-trait associations across population groups. The existing implementation of the Scalable and Accurate Implementation of Generalized mixture model (SAIGE) algorithm (10)—ideal for our design in order to address case/control imbalances—was not analytically tractable at this scale of computation and would have required ~251 compute years to complete. As such, we enhanced the computational efficiency of this algorithm with baseline improvements, implemented graphics processing unit (GPU) optimization for performing matrix operations, and completed analyses on the US Department of Energy (DOE)’s Oak Ridge Leadership Computing Facility Summit and Andes systems. Using this framework, we conducted a total of 4045 independent GWASs for traits that met QC criteria in each population group (table S2). The actual analysis took 14,286 GPU hours (14 days of wall time), leading to an overall 160-fold reduction in the core hours required.

The relatively large sample size, in particular among the AFR and AMR population groups as compared to the published literature, facilitated substantial discovery even at the stringent study-wide significance level of *P* < 4.6 × 10^−11^ (table S3). In the AFR group there were 2447 significant loci across 339 traits, including 1470 locus-trait associations not previously reported. Among these, a locus was identified on chromosome 15 that was associated with keloid scar formation (*P* = 2.2 × 10^−11^), a condition three times more prevalent in the AFR group compared to the EUR group in the MVP cohort. In the AMR group there were 1105 significant loci across 255 traits, including 341 locus-trait associations. In the EAS group we found 61 significant locus-trait pairs, including four previously unreported locus-trait associations. In the EUR group, the largest population, there were 23,628 significant loci across 814 traits. Notably, 36.6% (8651) of these loci were linked to quantitative traits and 10.9% (2578) were linked to binary traits, previously not reported in the NHGRI-EBI GWAS (11) and Open Target Genetics catalogs (12). We have made all summary statistics, phenotype definitions, and optimized code publicly available to facilitate global research endeavors (see data and materials availability).

### Population-specific heritability and genetic correlation patterns for complex traits demonstrate substantial similarity between groups

To characterize phenotypic variation attributable to common genetic variants across the four major population groups, SNP heritability was calculated using linkage disequilibrium score regression (LDSC) with population-specific GWAS results and in-sample LD reference panels (9). This analysis identified significant (*P* <9× 10^−6^) SNP heritability for 233 traits (*n =* 1525, mean h^2^ = 20.5%) in the AFR group, 199 traits (*n=* 1226, mean h^2^ = 22.1%) in the AMR group, three traits (*n=* 353, mean h^2^ = 50.9%) in the EAS group, and 816 traits (*n =* 1898, mean h^2^ = 12.2%) in the EUR group (fig. S1A and table S4). Height was the most heritable trait across all four population groups, consistent with a previous report (13). Between-group differences in the number of significantly heritable traits were largely due to sample size and power.

There were 287 distinct traits with significant heritability in both the EUR group and another population group (fig. S1B), we analyzed their cross-population genetic correlation (461 trait-population pairs) using Popcorn (13). In contrast to LDSC (14), which calculates the genetic correlation between two traits in the same population group, Popcorn calculates the genetic correlation for a single trait between two population groups. With EUR as the reference group, 168 of 236 traits were significantly heritable in both the AFR and EUR groups and exhibited a significant genetic correlation (*P* < 2.1 × 10^−4^, 0.05 ÷ 236 traits); 16 of 199 traits had significant genetic correlation between the AMR and EUR groups (*P* < 2.5 × 10^−4^, 0.05 ÷ 199 traits); and two of five traits had significant genetic correlation between the EAS and EUR groups (*P* < 0.006, 0.05 ÷ five traits, table S5). Specifically, between the AFR and EUR groups, the trait with the strongest genetic correlation among quantitative traits was height (ρ_gi_ = 0.66), whereas among the binary traits it was type 2 diabetes (ρ_gi_ = 0.65) (table S5). We also observed that certain traits exhibited weaker correlations between these population groups. For instance, skin cancer showed a correlation of ρ_gi_ = 0.05 and anemia from chronic disease had a slightly higher correlation of ρ_gi_ = 0.08. Additionally, iron levels and white blood cell counts demonstrated correlations of ρ_gi_ = 0.18 and ρ_gi_ = 0.20, respectively.

### Multipopulation meta analysis improves the power to detect associations not detected in the EUR population alone

**Fig. 3.**
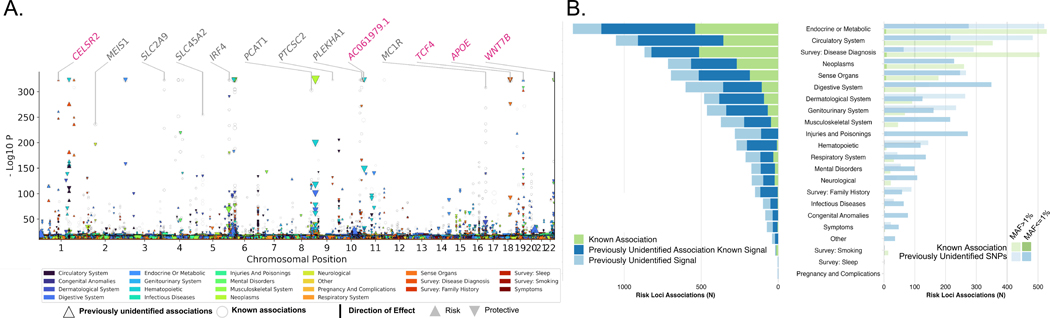
Multipopulation genetic associations with 1092 binary traits. Combined multitrait Manhattan plots and bar plots summarizing 8170 locus-trait associations for quantitative traits (*P*-value < 4.6 × 10^−11^). (**A**) Manhattan plot for binary traits displays associations across chromosomes (*x*-axis) and −log10P values (*y*-axis). Circles represent previously reported associations and triangles indicate previously unidentified trait associations. Triangle size corresponds to effect size, with upward triangles denoting risk associations and downward triangles signifying protective associations. On the top, gene names are highlighted to indicate previously reported variant trait associations (in black) and new trait associations (in pink). (**B**) Stacked bar plots for quantitative traits showcase the number of associations with locus-trait pairs across different trait categories. The left panel presents the count of known associations (green), previously unidentified trait associations (blue), and previously unidentified SNPs (light blue). Trait categories are ordered by the number of lead SNPs in descending order. The right panel is a dodged bar plot highlighting associations with lead SNPs based on their MAF categories: common variants (lower opacity) and low-frequency variants (higher opacity). The distribution of known associations (green) and previously unidentified SNPs (light blue) is shown for each trait category.

**Fig. 4.**
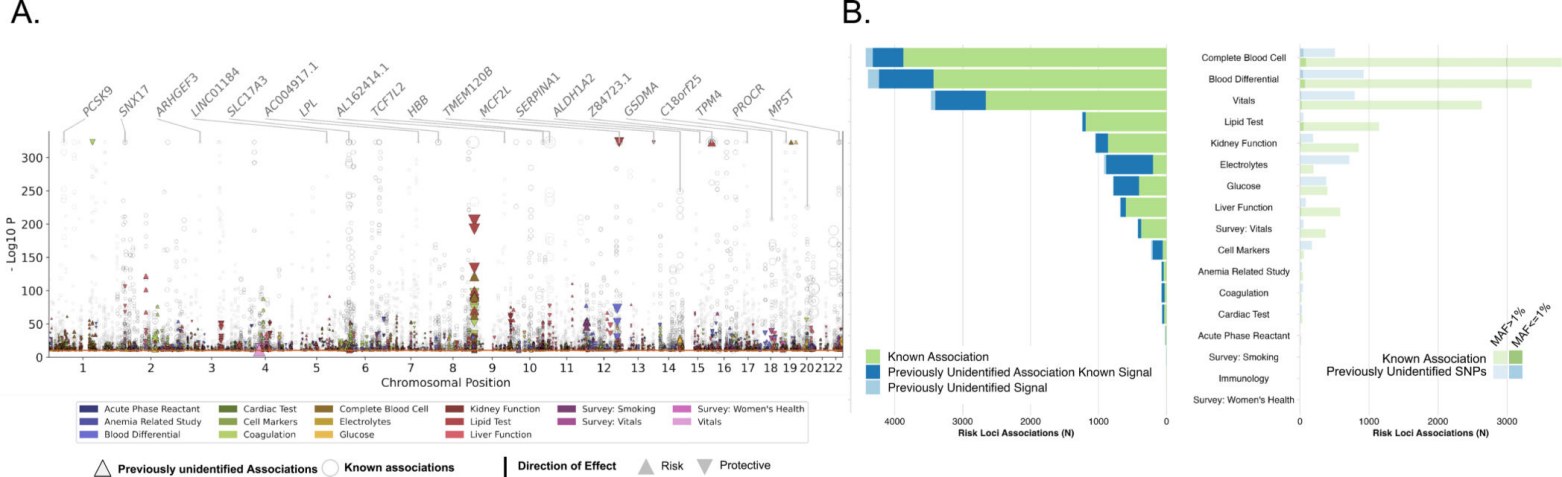
Multipopulation genetic associations with 178 quantitative traits. Combined multitrait Manhattan plots and bar plots summarizing 17,879 locus-trait associations for quantitative traits (*P*-value < 4.6 × 10^−11^). (**A**) Manhattan plot for quantitative traits display associations across chromosomes (*x*-axis) and −log10P values (*y*-axis). Circles represent previously reported associations and triangles indicate previously unidentified trait associations. Triangle size corresponds to effect size, with upward triangles denoting risk associations and downward triangles signifying protective associations. On the top, gene names are highlighted to indicate previously reported variant trait associations (in black) and new trait associations (in pink). (**B**) Stacked bar plot for quantitative traits showcasing the number of associations with locus-trait pairs across different trait categories. The left panel presents the count of known associations (green), previously unidentified trait associations (blue), and previously unidentified SNPs (light blue). Trait categories are ordered by the number of lead SNPs in descending order. The right panel is a dodged bar plot highlighting associations with lead SNPs based on their MAF categories: common variants (lower opacity) and low-frequency variants (higher opacity). The distribution of known associations (green) and previously unidentified SNPs (light blue) is shown for each trait category.

To better understand the genetic factors influencing complex traits across population groups, we carried out a multi-population meta analysis of our GWAS results. This approach facilitated the identification of genetic risk loci that were similar or different across the population groups and enhanced our ability to draw insights from non-EUR populations. We identified 26,049 associations (13,672 loci for 1270 traits) with a study-wide significance of *P* < 4.6 × 10^−11^ (Methods, Fig. 3, and table S6); 1092 binary traits (on average, 21 mean associations per trait, Fig. 3A) and 178 quantitative traits (on average, 421 mean associations per trait, Fig. 4A) exhibited significant associations. The mean genomic inflation factor across all traits was 1.01 (range from 0.85 to 1.19), indicating that the test statistic error rates were relatively controlled (fig. S2). We found that 72% (5885) of locus-binary trait associations (Fig. 3, A and B, and tables S7 and S8) and 23% (4104) of locus-quantitative trait associations (Fig. 4, A and B, and tables S7 and S8) were not previously identified (9) in the NHGRI-EBI GWAS (11) and Open Target Genetics catalogs (12). In fact, 11% (432) of the variants associated with quantitative traits and 34% (1986) with binary traits have not previously been associated with any other trait, likely due to our ability to interrogate low frequency and rare alleles as approximately 57% of these risk variants had MAF < 1% (Figs. 3B and 4B).

To quantify the discoveries made through expanding representation of understudied populations in genetic analysis, we compared the results of the multipopulation meta analysis to those of the EUR-only GWAS. Over half of the variants analyzed in the meta analysis were not included in the EUR group GWAS as a result of MAF or imputation quality and a quarter (10M) were only present in AFR (9). The inclusion of individuals genetically similar to AFR, AMR, and EAS reference populations identified 1608 additional genomic loci, which were not significant (P > 4.6 × 10^−11^) in the EUR-only analysis (table S9). This led to a total of 3477 variant-trait associations across 893 traits, 76% of which were with binary traits. The most significant of these results was a rare intronic variant, rs72725854, located near the long non-coding RNA (lncRNA), *PCAT2*, associated with prostate cancer (table S9). This SNP is low-frequency in African populations but exceedingly rare in other groups (MAF_AFR_ = 0.06, MAF_AMR_ = 0.0068, MAF_EUR_ = 0.0006) and has been previously reported to increase the risk of prostate cancer twofold, aligning with our study findings. We also replicated findings previously reported from AFR analyses, such as *ACKR1* for neutropenia and reduced white blood count levels (15) and a missense variant in *APOL1* (rs73885319) with kidney-related conditions such as end-stage renal disease (table S9) (16).

Moreover, we identified 834 variant-trait associations primarily driven by the inclusion of participants from non-EUR populations; these associations were not even nominally significant in the EUR group (P > 0.05, table S9). We identified an AFR-specific noncoding index variant in *FAM234A* associated with iron deficiency anemias (P_AFR_ = 2.37 × 10^−37^, P_AMR_ = 0.05, P_EUR_ = 0.42, table S9) and hereditary hemolytic anemias (P_AFR_ = 5.32 × 10^−33^, P_AMR_ = 0.28, P_EUR_ = 0.25, table S9) only in the AFR group. We also observed an association between rs3104394 in *MTCO3P1* with alopecia areata only in the AMR population (P_AFR_ = 0.01, P_AMR_ = 1.27 × 10^−11^, P_EUR_ = 7.66 × 10^−6^). Although there is no information available about the relationship between the *MTCO3P1* gene and alopecia, a cross sectional analysis of the NIH All of US cohort found that alopecia areata is more prevalent in Hispanic/Latinx individuals, suggesting potential genetic factors contributing to the development of this condition (17).

### Fine-mapping of multipopulation associations reveals single-variant credible sets

**Fig. 5.**
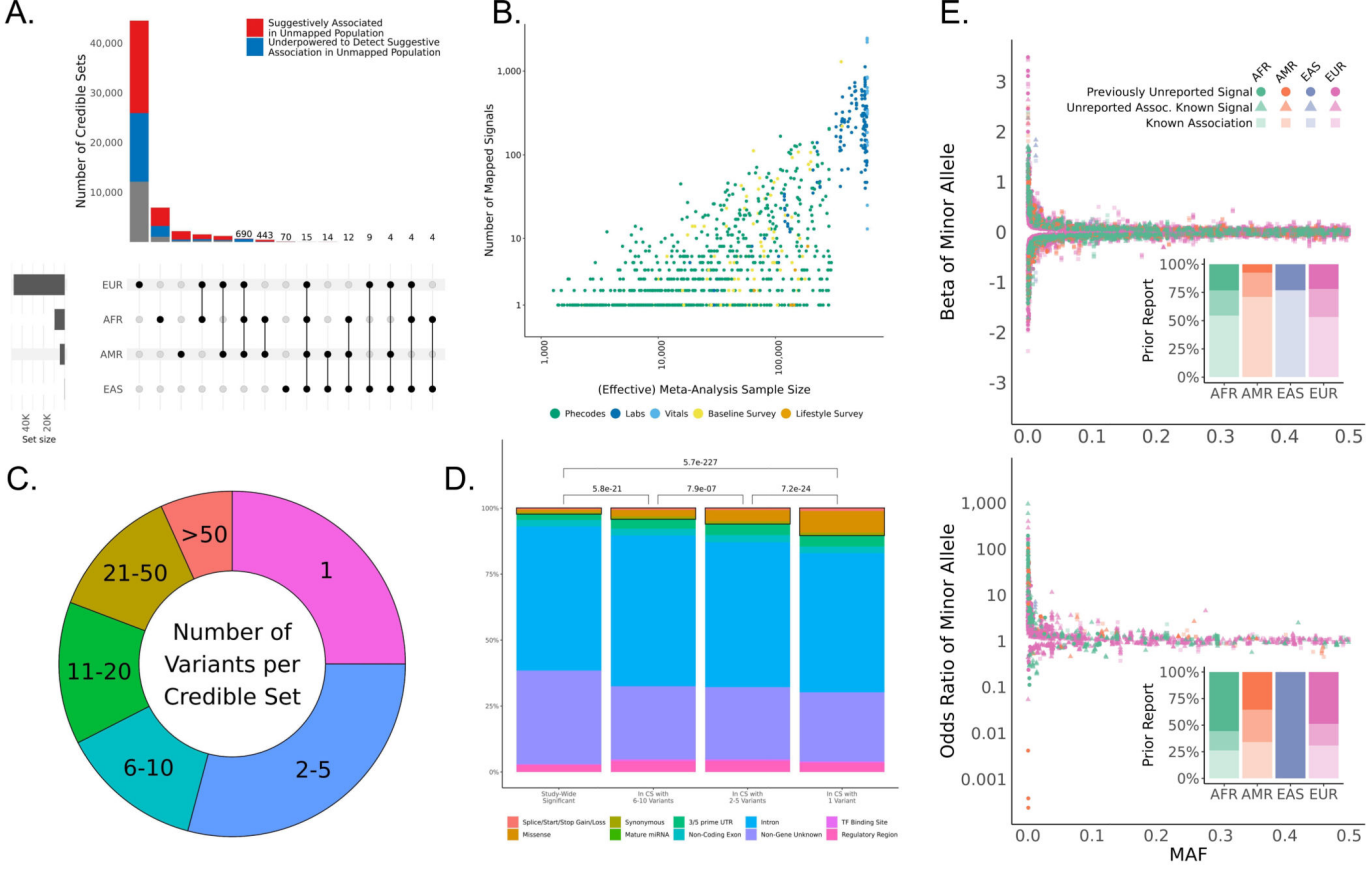
Multipopulation fine-mapped signals. (**A**) Upset plot of cross-population signal sharing for the 57,601 fine-mapped signals. Red portions of bars represent signals that had one or more variants showing suggestive association (*P*-value < 1×10^−3^) in an unmapped population and blue portions represent signals where the unmapped populations were underpowered in the unmapped ancestries to detect suggestive associations for any of the variants in the merged approximate credible set. Signal counts are displayed above the bars for intersections in which fewer than 1000 signals were identified. (**B**) Scatter plot of the number of signals detected per phenotype versus the meta analyzed sample size for the phenotype. Effective sample sizes were used for binary phenotypes and points are colored by the phenotype category. (**C**) The distribution of merged approximate credible set sizes for the fine-mapped signals. (**D**) Coding enrichment in precisely mapped signals. Bars are colored by the proportion of each represented by each grouped Variant Effect Predictor (VEP) annotation and the black boxes illustrate the proportion of each bar attributable to coding variation. *P*-values reflect the results of Fisher exact tests for coding annotation enrichment. (**E**) Distribution of effect sizes versus minor allele frequencies for high-confidence (PIP > 0.95) associations fine-mapped in quantitative (top) and binary (bottom) phenotypes. Each point represents a unique high-confidence variant-phenotype-population mapping. Point colors reflect the population in which they are mapped and their shapes reflect whether they are a phenotype association previously reported in the GWAS catalog (square), a phenotype association previously unidentified for a signal already reported in the catalog (triangle), or a signal and association that were both previously unidentified (circle). Inset bar plots reflect the proportions of high-confidence associations in these three categories across the four tested populations.

To create a catalog of putative causal genetic variants that could be qualitatively and quantitatively compared across population groups, we performed within-population group fine-mapping using the Sum of Single Effects model implemented in SuSiE (18, 19) followed by multipopulation credible set integration. We defined 25,953 locus-trait pairs, corresponding to 1257 traits with one or more study-wide significant variants outside the major histocompatibility complex (MHC) (fig. S3). We fine-mapped 99.96% of these pairs within each population group using exact, in-sample matched linkage disequilibrium (LD) matrices for the trait and identified signals at 22,866 (88%) of the pairs (fig. S3) (9). The 0.03% of locus-trait pairs that failed to map were primarily due to computational constraints (table S10). The fine-mapped signals included 15,045 distinct variant-trait pairs (6318 variants and 613 traits) that mapped with high confidence, meaning a posterior inclusion probability (PIP)> 0.95 in one or more populations. We merged signals across populations based on their Jaccard similarity indices (20) and identified 57,601 multipopulation signals across 936 phenotypes (fig. S3 and tables S11) (21); 53,669 (93.1%) of the signals were mapped in a single population including 44,516 (77%) that were fine-mapped in only the EUR group (Fig. 5A). However, we note that >75% of the signals that were fine-mapped in only a single population turned out to either be modestly associated (P < 1 × 10^−3^) with the same allele implicated as trait increasing in one or more populations not subjected to fine-mapping, or the underlying GWAS was simply under-powered to detect a suggestive association in the unmapped population (at less than 80% power). A larger effective overall sample size and thus greater power was correlated with a larger number of mapped signals (Fig. 5B), likely explaining why most signals were seen in the EUR group. Among the 15,045 high-confidence pairs, 2069 variant-trait associations were fine-mapped with high confidence only in the non-EUR groups (table S12). These associations correspond to 974 unique variants and 271 traits. To quantify the precision of fine-mapping for the multipopulation results, we defined an “approximate” credible set for each Jaccard-similarity population-aligned signal as the union of variants in each population-level credible set. Despite this definition, we observed that >54% of the merged signals identified by the fine-mapping pipeline contained ≤5 variants and 14,405 (25%) contained a single variant (Fig. 5C). Although there is no gold standard for validating the accuracy of credible sets we observed notable enrichments in fine-mapped signals for genomic anotations with known functional roles, namely coding variation (Fig. 5D), as well as higher functional prediction scores from RegulomeDB (fig. S4).

To compare the relative precision of fine mapping between population groups, we determined whether there was a difference in the size of our approximate credible sets for signals that mapped in multiple groups. Signals identified in both the AFR and EUR groups generally had slightly but significantly smaller sets when mapped in the AFR group than that of the EUR group (Wilcoxon signed-rank *P* = 2.26 × 10^−10^; fig. S5). By contrast, our approximate credible sets in the AMR group were larger than their AFR group (*P* = 1.30 × 10^−84^, fig. S5) and EUR group counterparts (*P* = 7.36× 10^−162^, fig. S5). Believing that sample size influenced the ability to detect signals and the sizes of credible sets, we downsampled the EUR group to match the AFR group in terms of age, sex, and the overall numbers of affected and unaffected individuals for the traits of interest. We then reanalyzed the 2142 traitloci pairs where at least one signal was detected in both the AFR and EUR groups. After downsampling, we were able to detect only 858 of the original 2236 shared signals. More importantly, differences in credible set sizes for the 858 remaining shared signals also grew, with credible sets from the AFR group notably smaller than their EUR counterparts (Wilcoxon signed-rank P = 3.8 × 10^−52^; fig. S6), thus demonstrating that the presence of smaller LD-blocks in the AFR population, as compared to the EUR population, permits more accurate fine-mapping at a given sample size.

We next analyzed the distribution of effect sizes and allele frequencies for lead variants and fine-mapped signals for the 15,822 variant-trait-population combinations with high confidence (PIP > 0.95) fine-mapped signals. Consistent with previous reports (22, 23), we observed an inverse relationship between the minor allele frequency of a variant and its effect size for both lead variants (fig. S7) and high-confidence signals (Fig. 5E) across all four population groups. For the high-confidence signals, we examined the relationship between frequencies and effect sizes for alleles derived in the human lineage since the last common ancestor of chimpanzees and bonobos (fig. S7). As 87% of derived alleles were minor alleles, it was not surprising that we observed strong effects for variants with allele frequencies close to zero. Large effect sizes were also observed for several variants whose derived allele was high frequency; some of these map to previously reported targets of positive selection in human populations (24–26). We observed this relationship between allele frequency and effect size for both newly observed variant-trait associations and those previously reported in the GWAS Catalog (11), with similar relative proportions in the three well-powered population groups (AFR, AMR, and EUR).

We next observed that the distribution of effects in binary and quantitative phenotypes was different. Although it was equally common for minor and derived alleles at high-confidence signals to associate with an increase or decrease in a quantitative trait, such as higher white blood cell count (WBC) or lower WBC (49.6% of minor and derived alleles were associated with a higher value of the quantitative trait), the majority (71%) of these alleles conveyed increased risk for binary traits (Fig. 5E). The increased risk effect among minor alleles was also observed for lead variants; 73% of lead-SNP minor alleles increased the risk (fig. S7).

Finally, we screened for heterogeneity of estimated effect size across common signals (MAF > 0.05) at 1888 fine-mapped loci (representing 1329 separate traits) with overlapping credible sets in multiple groups. We identified 16 heterogeneous variant-trait associations when comparing the AFR to the EUR group and 11 when comparing the AMR to the EUR group (table S13). Focusing on coding variants that mapped to the same trait with high confidence (PIP > 0.95) in multiple populations, we observed six associations with marked heterogeneity in effect size between the estimates in the AFR and the EUR groups, and two when comparing the AMR group to the EUR group (table S14). All variant-trait pairs had the same direction of effect. Most of the differences across signals mapped to rs429358-C, the coding variant tag for APOE-e4 associated with a 30% lower risk of dementia in the AFR compared to the EUR group (27). There was also marginal heterogeneity between the AMR and the EUR group while the EAS group was underpowered for this analysis.

### Characterization of fine-mapped associations specific to non-EUR population groups

Recognizing the power of our study to elucidate biology among population groups traditionally understudied in human genetics, we sought to interrogate the fine-mapping results between populations. Of the 25,953 high-confidence variant-trait pairs identified by fine-mapping, 2069 (974 unique variants and 271 phenotypes) were unique to the analyses of the non-EUR groups (table S12). Although most of the signals were from low-frequency or rare variants, 15 previously unreported signals (10 AFR, 3 AMR, 2 EAS) were located in coding variants and had a MAF > 0.05. Among these was a missense variant, rs73382631, associated with lower WBC and neutrophil counts in the AFR group (MAF_AFR_ = 0.10, MAF_AMR_ = 0.01, not present in the EAS or EUR group). Another example was a missense coding variant in *ABCG2* (rs35965584, MAF_AFR_ = 0.002, not present in AMR, EAS, or EUR groups), for which our analysis identified an association with gout not found in previous studies. Previous reports have identified an association between *ABCG2* and hyperuricemia (28) and susceptibility to gout (29), with another known *ABCG2* missense variant (rs2231142) (30). In MVP, the previously identified missense variant rs2231142 was within the 95% credible set of a distinct gout signal (*n* = 8 variants) mapped in EAS and EUR groups but was not in linkage disequilibrium with rs35965584 (r^2^ = 0.0001).

Most of the population group-specific signals were in noncoding regions. To gather insights into these variants, we used functional prediction scores from RegulomeDB (table S12), identifying 43 previously known associations and 20 previously unreported associations with SNPs that had strong evidence of regulatory activity (RegulomeDB score > 0.9). The previously reported loci were associated with factors such as hemoglobin A1c, cholesterol measures, heart rate, red blood cell count, and type 2 diabetes (31, 32). All other newly identified associations were related to quantitative traits, such as rs574674363 and lower high-density lipoprotein (HDL) cholesterol levels.

### Cross-trait genetic architecture identifies pleiotropic genes

Next, we identified putative causal genes associated with fine-mapped variants using a two-step nomination scheme (fig. S8). First, we intersected the fine-mapped variants with exons of protein-coding genes based on Gencode release 19 annotations. In the second step, we utilized the Activity-by-Contact (ABC) (33) model, which allowed for the nomination of additional genes associated with synonymous and non-coding variants. This involved intersecting active promoter and enhancer regions with the fine-mapped variants. Our approach identified 31,764 trait-variant-gene combinations representing 15,596 trait-gene associations. 20% of the nominations were through non-synonymous coding variants, 52% involved ABC interactions, and 28% were in ABC promoters (fig. S9 and table S15). Consistent with the power to detect associations in GWASs and signals in fine mapping, we observed a pattern where more genes were implicated in traits with larger sample sizes (fig. S9).

Seeking to demonstrate the plausibility of our nominated genes, we tested the genes associated with each trait for overrepresentation in KEGG pathways. We identified 467 KEGG pathways that were overrepresented across 142 traits (table S16), which largely reflected known biology for their respective traits.

**Fig. 6.**
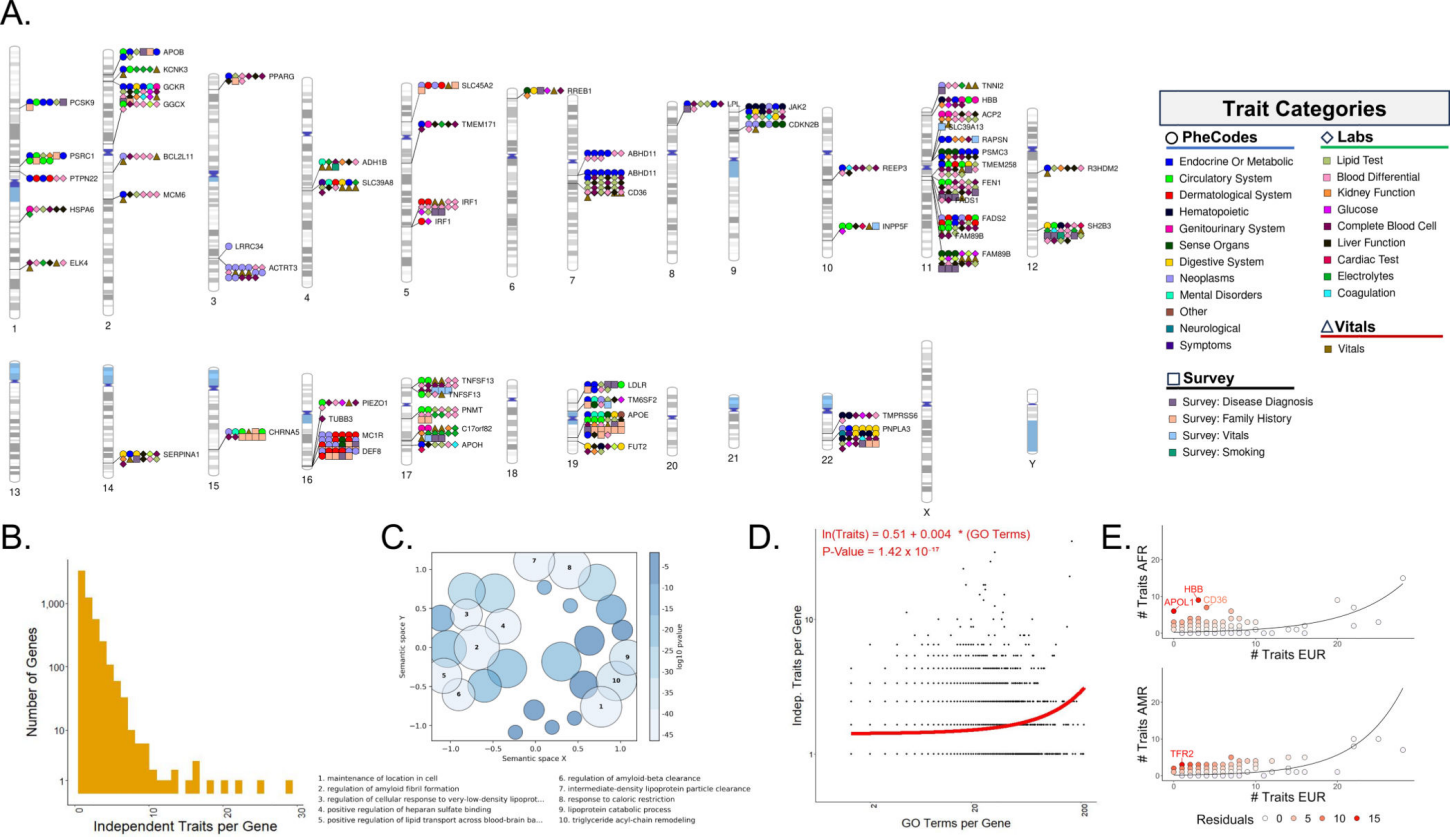
Putative causal gene and gene-level pleiotropy. (**A**) Chromosome ideogram illustrating high-confidence cross-trait associations (PIP > 0.95) between genetic variants and independent traits. The ideogram highlights putative causal gene nominated using non-synonymous coding variation and Activity-by-Contact (ABC) promoters and enhancers to implicate genes for fine-mapped variants. (**B**) Histogram of the number of independent traits identified per gene. (**C**) GO-Figure plot showing clusters of biological process GO terms that are significantly predictive of the number of independent traits associated with each gene. (**D**) Scatter plot and Poisson regression of the number of independent traits per gene on the number of GO terms annotated per gene. (**E**) Scatter plot and Poisson regression of the number of independent traits per gene in the AFR and EUR groups (top) and the AMR and EUR groups (bottom). Genes with the greatest residuals from the regressions have been labeled.

2279 genes associated with two or more genetically independent traits, resulting in 6711 pleiotropic associations (table S17). 70 genes (677 associations) associated with seven or more independent traits (Fig 6A). In particular, *APOE* was the most pleiotropic gene, linked to 29 different traits, including previously identified conditions such as HDL levels, macular degeneration, abdominal aortic aneurysm, and Alzheimer’s dementia. We also observed previously unreported associations between this APOE and chronic liver disease and cirrhosis.

To further investigate the functional role and pleiotropy of the nominated genes, we assessed whether membership in specific gene ontology (GO) categories was predictive of the number of genetically independent traits associated with each gene. 567 GO terms associated with gene pleiotropy, each of which had an increasing effect on the number of independent traits identified per gene (table S18). After clustering these GO terms based on their semantic similarity, we observed that a small set of highly pleiotropic genes, including *APOE*, *PNPLA3*, *GCKR*, and *JAK2*, were responsible for the GO clusters with the most correlated GOterms (Fig. 6C and fig. S10). Recognizing that all significant GO associations had increasing effects on gene-level pleiotropy, we also interrogated the relationship between the number of GO terms annotated per gene and the number of traits associated with the gene using a Poisson generalized linear model. This analysis yielded a highly significant positive relationship (P = 1.4 × 10^−17^, Fig. 6D).

At the gene level, 549 of the 15,596 gene-trait associations were only identified through variants that are either monomorphic or ultra-rare (MAF < 0.1%) in the EUR group (table S16). For example, *SLC22A18*, a known tumor suppressor (34, 35), and its antisense transcript *SLC22A18AS* were both associated with keloid scarring through rs76024540, a variant that was common in the AFR group (MAF ~11%) but monomorphic in the EUR group. rs76024540 was found in an ABC enhancer that interacts with the promoter of both genes in numerous cell types, including several comprising skin tissues.

Lastly, we sought to identify genes that were pleiotropic outliers in the AFR or the AMR groups, relative to the EUR group. To do this, we separately considered the genes nominated by variants mapped in each of the three population groups. Most genes were associated with more independent traits in the EUR group than in either the AFR or AMR groups, and the relationship between the number of independent traits per gene in the non-EUR and EUR groups could be well-modeled through a Poisson regression (Fig. 6E and table S19). However, in comparing the AFR and EUR variant-gene-trait mappings, a handful of genes with known AFR-specific variants and roles in disease etiology were found to deviate substantially from the observed relationship and were more pleiotropic in the AFR than the EUR group. This list of outliers was led by *APOL1*, *HBB*, and *CD36*, all hypothesized to confer some survival advantage to trypanosome or malarial infection (36). Because of the limited sample size in the EAS group we only observed 62 trait-gene nominations prior to pruning traits by their genetic correlations. This was too few to confidently conduct the Poisson Regression and determine outlier genes.

## Discussion

In this study we present a series of comprehensive phenome-wide GWASs analyses conducted within the VA Million Veteran Program, the largest multipopulation biobank to date, with a diversity that supports large-scale analyses of similarities and differences between variants and traits across populations. We studied 44.3 million variants across 2068 traits among 635,969 US Veterans, of which 613 traits were fine-mapped with high precision. Cross-population analyses identified 834 previously unreported variant-trait associations driven by the inclusion of individuals not genetically similar to European reference populations, 15 signals from coding variants that are either rare or not observed to be present in these populations, and numerous genes that had pleiotropy predominantly among individuals genetically similar to African reference populations; this highlights the substantial contribution conferred by including diverse populations in genetic research. At the same time, cross-population heritability analyses, fine mapping, and heterogeneity analyses demonstrated substantial similarities in the genetic architecture between population groups driven by variants common across populations.

Our analyses of variant-trait associations detected several signals that would not have been identified in a GWAS comprising solely individuals genetically similar to EUR reference populations. One example is rs72725854 at the *PCAT2* locus, which has been associated with an increased risk of prostate cancer identified in prior studies (37–39). Prostate cancer is more common in self-reported Black men compared to the general population, and approaches that incorporate this variant and others into risk scores are under active investigation as a component of precision medicine–based prostate cancer screening approaches. Additionally, the previously unidentified signal for gout among the AFR population group at rs35965584 may represent an independent *ABCG2* signal for gout risk, in addition to the known variant rs2231142 (30). As many risk factors are associated with gout, whether this signal contributes to the observed higher prevalence of gout in self-reported Black compared to self-reported White populations requires further study (40). Finally, the association with keloid scarring of the variant rs76024540 at the *SLC22A18/SLC22A18AS* locus, common among individuals genetically similar to AFR reference populations but not in other population groups, may point to a genetic etiology for the increased prevalence of this condition among individuals genetically similar to AFR reference populations compared to other population groups. These findings exemplify how the inclusion of individuals from diverse populations in human genetics experiments generates important insights into health and disease traits that may disproportionately affect these groups.

Our analysis expands on previous, large-scale fine-mapping experiments (20, 41, 42) aimed at determining candidate causal variant(s), substantially increasing the number of fine-mapped traits and signals, particularly among individuals genetically similar to AFR and AMR reference populations, which have traditionally been underrepresented in genetic studies. Additionally, the increased representation of diverse participants in our analysis facilitated improved precision of fine mapping. Notably, the analysis among the AFR group yielded the most precise approximate credible sets of our three well-powered groups, followed by the EUR and the AMR groups. This finding was anticipated because haplotype blocks are smaller in populations that are genetically similar to African reference populations (43, 44). Despite the paired Wilcoxon test showing greater precision in the credible set sizes for the AFR group, it did not lead to the expected variation in median credible set sizes between the AFR and the EUR groups, with the median difference being zero. However, through a downsampling experiment in which we matched the size of the EUR group to that of the AFR group, we demonstrated that the absence of difference stems from the larger sample size in the EUR group, which in turn boosts statistical power. Thus, we expect that the inclusion of increasing numbers of diverse individuals will continue to improve the precision of signal fine mapping efforts, and newer signal fine-mapping methods which fully leverage LD differences across populations to identify independent signals will further refine credible sets.

The fine-mapping analyses also demonstrated overwhelmingly more similarities than differences in the genetic associations between groups. The vast majority of differences observed were largely attributable to variations in allele frequency or the presence of genetic variants in one group that were not detectable in other groups. In fact, among the most common variants mapped with high precision, there was minimal evidence of heterogeneity in effect estimates. The *APOE* locus was a notable exception, where we observed an association between the high-confidence fine-mapped signal rs429358 and increased risk for dementia across all four population groups examined. However, the risk was 30% lower in the AFR group compared to the EUR group, corroborating prior studies that observed differential risk between *APOE* alleles and dementia in non-EUR compared to EUR populations (27, 45). Additionally, while *APOE* was among the most pleiotropic genes across all populations, among the AFR group, *HBB* had associations with traits beyond sickle cell anemia, including gout.

Analysis of genome-wide architecture through the genetic correlation of individual traits across population groups demonstrated largely preserved, rather than divergent, genetic architectures. The weaker correlations are likely driven by the association with variants that had a higher allele frequency in specific population groups. The limited correlation observed between the EUR and the AMR groups is primarily due to the inherent limitation described in the Popcorn method, which does not adequately account for the long-range linkage disequilibrium (LD) present in admixed populations. Overall, these findings imply that, with the exception of population-specific variation in allele frequency, foundational genetic architecture is more similar than different across diverse populations.

Our work must be interpreted within the context of its limitations. First, to efficiently conduct large-scale GWAS analyses across the phenome, we used an automated approach for phenotyping. This approach involved using Phecodes for collating clinical diagnosis codes; while efficient, it could be more precise for most phenotypes. Similarly, our regression models also had to be standardized, accounting only for age, sex, and principal components, and performing inverse-normal transformations to quantitative traits prior to analysis. Undoubtedly trait-specific bespoke phenotype definitions and regression modeling would have improved power for variant discovery. Second, we applied the widely accepted 50% probability threshold for population assignment in our study, categorizing genetic diversity into discrete groups. This method led to the exclusion of 5,953 participants, who constitute less than 1% of our total study population. While aiming to reduce within group heterogeneity for more robust genetic analysis, this approach introduces a significant limitation by potentially neglecting the complex admixture present in genetic data. Third, while applying LD score regression and Popcorn analysis to evaluate genetic architecture across various traits, we acknowledge the limitation of assuming uniform polygenicity and homogenous genetic distribution, which may not hold true for all phenotypes. Fourth, despite the diversity of MVP, the cohort still mostly comprises individuals similar to European reference populations, which, together with varying disease prevalence across population groups, leads to differential power to detect and fine-map causal variants across. Fifth, in order to perform fine-mapping at this scale, we had to make a number of compromises in our analytic approach. Our method of defining loci has potential pitfalls as it is based on meta analyzed data, not considering whether the population-specific GWAS peak was present. Consequently, we fine-mapped some regions with no significant signals, especially within the EAS group, which was the smallest population group and had limited power. Our approach may have also overlooked group-specific peaks eclipsed in the meta analysis and certain loci too vast to be completely mapped under our scheme. Our conservative approach, adhering to a minimum threshold of significance and purity for signals to maintain precision (positive predictive value), could result in missing true signals. Similarly, our preference for precision over recall (sensitivity) meant we limited the fine-mapping to a maximum of five signals per locus. This approach can lead to an underestimation of the number of signals at certain highly significant loci. We also encountered challenges in deploying the fine-mapping method at this scale. In particular, the LD matrices used did not ideally synchronize with the SAIGE methodology due to our reliance on hard-called genotypes and not accounting for covariates. This could have led to minor LD mismatches, which may influence sensitive loci, resulting in inaccuracies or spurious results in the fine-mapping stage. Future research may consider these constraints and propose alternative approaches to further enhance the validity and comprehensiveness of the results. Lastly, while diverse in ancestry, the Veteran population is predominantly male and older than the general US population. Thus, this study is less well-powered to study conditions more prevalent in females or younger populations.

Diversity is critical in advancing genomic studies, providing foundational data for downstream implementation ranging from risk prediction to targeted therapeutics. Despite efforts from large biobanks such as UK Biobank, FinnGen, and Biobank Japan, lack of diversity in genomic studies remains a challenge. As of this writing, the MVP has enrolled its 1 millionth participant, with over 175,000 participants genetically similar to the African population, making it the biobank with the greatest representation of this population group (5). Recently, additional diverse biobanks, such as the All of Us Study Research Program (6), America Latino Research Biobank (46), and Human Hereditary and Health in Africa (H3Africa) (47), as well as hospital and institutional biobanks have been established and continue to grow. Since its inception, the MVP has aimed to encompass a population representative of the diverse United States Veteran community. Our comprehensive phenome-wide GWASs presented here underscores the increased power of discovery that comes from including individuals from diverse populations, enriching our understanding of the genetics of complex health and disease traits, while highlighting the large degree of similarities in genetic architecture of these traits across populations.

## Methods summary

We conducted GWASs of 2068 traits across four population groups defined by genetic similarity to the 1000 Genomes Project AFR, AMR, EAS, and EUR reference superpopulations (8). The 635,969 individuals comprising the four groups were participants in the VA’s Million Veterans Program (MVP), and the 2068 traits were composed of diagnosis codes, laboratory measures, vital signs extracted from the VA EHR and trait derived from survey questionnaire at enrollment. We executed the GWASs with a GPU-optimized version of SAIGE on the DOE’s Summit supercomputer (48, 49).

Following up on each GWAS, we used LDSC (50) to identify traits with significant heritability in each of the four separate populations and Popcorn (13) to identify traits with significant genetic correlations across distinct population groups. Significant loci were then defined based on a threshold of *P* < 4.6 × 10^−11^, which was set by calculating the number of independent traits in our study: 1038. We determined genomic loci and lead variant via LD clumping in Plink 1.9 (51) using a two-tiered approach similar to that previously described in FUMA (52). Following comparison of population-specific GWAS results and trait prevalences, GWASs were meta analyzed across populations using the fixed-effect, inverse-variance weighted method implemented in GWAMA (53). We compared the meta analysis to the EUR-specific results to quantify the additional loci identified through the non-EUR contribution to our study. Moreover, we also checked lead variants for previous reporting in the NHGRI-EBI GWAS Catalog (11) and Open Targets (12) databases.

To identify causal variants, we fine-mapped the population-specific GWASs using SuSiE (18, 19) and in-sample LD matrices matched to each trait. We compared signal counts across populations and across traits. Moreover, we also compared allele frequencies and the previous reporting status of high-precision fine-mapped signals (PIP > 0.95) across populations. To validate the observed credible sets, we used VEP (54) and RegulomeDB (55) to annotate the fine-mapped variants and detect functional enrichments in precisely mapped signals. We also compared fine-mapping precision across populations using signals mapped in multiple groups. As part of this analysis, we down-sampled the EUR population to match the size and composition of the AFR group thereby controlling for the effect of sample-size on precision. Additionally, we tested for effect size heterogeneity across common signals (MAF > 0.05) at the 1888 fine-mapped loci with overlapping credible sets in multiple groups.

In a final analysis, we leveraged the fine-mapped signals to nominate effector genes for traits by leveraging nonsynonymous coding variation and regulatory connections predicted by the ABC model (33). We detected over-represented KEGG pathways (56) for each trait’s set of putative effector genes and leveraged the trait nominations to quantify the pleiotropy of each gene. To do so, we defined gene-level pleiotropy as the number of independent traits nominated for each gene as determined by iterative pruning of traits with a phenotypic correlation > 0.2. Using Poisson regression, we then identified GO terms significantly associated with overall gene-level pleiotropy (Benjamini-Hochberg adj. P < 0.05) and genes that are pleiotropic outliers when comparing AFR or AMR trait nominations with those made using EUR-mapped variants.

## Supplementary Material

Supplemental Text

Supplemental Table S1-10

Supplemental Table S12-20

Supplemental Table S11

MDAR Checklist

## Data Availability

Full summary statistics of all the GWASs are publicly available for public browsing and download through dbGAP (accession number phs002453). A PheWeb browser is available at: https://phenomics.va.ornl.gov/web/. Questions about access to summary statistics should be directed to MVP_gwPheWAS@va.gov. The GPU-based version of SAIGE is available on GitHUB (48) as well as Zenodo repository (49). Signal-level summary file of fine-mapping results can be accessed on Dryad (21).
