# Supplementary material for: Diversity and scale: Genetic architecture of 2068 traits in the VA Million Veteran Program
Source: Science. Author manuscript; Available in PMC 2026 Jan 30. (PMC12857194; doi:10.1126/science.adj1182)
Supplement: Supplemental Text [file NIHMS2091849-supplement-Supplemental_Text.pdf]

Science AAAS

Supplementary Materials for

Diversity and scale: Genetic architecture of 2068 traits in the VA Million Veteran Program

Anurag Verma *et al*.

Corresponding author: Scott Damrauer, scott.damrauer@pennmedicine.upenn.edu

Science **385**, eadj1182 (2024)
DOI: 10.1126/science.adj1182

The PDF file includes:

Materials and Methods
Supplementary Text
Figs. S1 to S14
References

Other Supplementary Material for this manuscript includes the following:

MDAR Reproducibility Checklist
Tables S1 to S20
Data S1

Materials and Methods

1. Data description

Million Veteran Program study cohort

The VA Million Veteran Program (MVP) is a national cohort launched in 2011 to determine the contributions of genetics, lifestyle, and military exposures to health and disease among US Veterans (*7*) Blood biospecimens were collected for DNA isolation and genotyping. The biorepository was linked with the VA electronic health record (EHR), which includes diagnosis codes (International Classification of Diseases ninth revision [ICD-9] and tenth revision [ICD-10]), laboratory measures, and detailed survey questionnaires collected at the time of enrollment for all Veterans and followed in the healthcare system until September 2020.

Genotyping and imputation

Genotyping and imputation methods for the MVP were described previously (*57*). In brief, the single nucleotide polymorphism (SNP) data in the MVP cohort were generated using a custom ThermoFisher Axiom MVP 1.0 genotyping platform, and imputation was performed to a hybrid reference panel comprised of the African Genome Resources panel (*58*) and 1000 Genomes Project (1000G) Phase 3 Version 5 (*59*). Following imputation, variant level quality control (QC) was performed, and genetic variants with a) imputation quality <0.3, b) minor allele count (MAC) <20, c) call rate <97.5% for common variants (minor allele frequency [MAF] >1%), and d) call rate <99% for rare variants (MAF<1%) were excluded. Additionally, variants were also excluded if they deviated >10% from their expected allele frequency based on 1000G reference data.

Population assignment

To estimate population group membership, we obtained a reference dataset from 1000G and used the smartpca module in the EIGENSOFT (*60*) package to project the PC loadings from a group of unrelated individuals in the reference dataset. We merged this dataset with the MVP dataset and ran smartpca to project the PCA loadings from the reference dataset. We trained a random forest classifier using continental ancestry meta-data based on the top 10 principal components from the reference training data to define genetically inferred ancestry. We then applied this random forest to the predicted MVP PCA data and assigned populations to individuals with a probability greater than 50%. Those with a probability less than 50% for any particular population group were excluded from the study. **Figure S11** shows the PCA projection of MVP participants on the 1000G reference panel. Individuals were assigned to populations groups defined as genetically similar to African (AFR), Admixed American (AMR), East Asian (EAS), European (EUR), and South Asian (SAS). Only the first four populations groups had enough cases to be included in these analyses. Lastly, we selected PC1-10 to adjust our analysis. **Figure S11** shows the variance explained by each PC.

Additionally, for compatibility with historuc MVP data we also utilized harmonized ancestry and race/ethnicity (HARE) (*61*) population groups and implemented the same quality control (QC) criteria. The HARE group's GWAS summary statistics are also available via the dbGAP ftp site.

Phenotyping

The initial phenotype data were accessed through the Centralized Interactive Phenomics Resource (CIPHER, https://phenomics.va.ornl.gov/), which consists of EHR-based phenotypes derived from various sources, including EHR diagnosis codes, clinical laboratory tests, and survey questionnaire responses. In this study, we used phenotypes from all three sources to examine the associations with genetic variants.

A) Phenotypes from ICD codes

Binary clinical outcomes from the electronic health records (EHRs) were defined using phecodes (*62*), which are curated groupings of ICD codes. Each phecode represents a group of International Classification of Diseases (ICD) codes clinically relevant for a particular phenotype. Using this approach, all ICD codes for all Veterans in the MVP cohort were extracted and assigned a phecode-defined phenotype. ICD-9 and ICD-10 codes were mapped to 1,876 phecodes. For each phecode, participants with at least two phecode-mapped ICD-9 or ICD-10 codes were defined as cases, while those with no instances of a phecode-mapped ICD-9 or ICD-10 code were defined as controls. Based on our previous simulation studies of EHR data (*63*), populations where the phecode comprises fewer than 200 cases are more likely to produce spurious results, so we applied this threshold within each population group (AFR, AMR, EAS, EUR). Additionally, the study excluded certain conditions considered protected at the VA, such as sickle cell anemia and HIV status, as their data cannot be reported broadly.

B) Phenotypes from laboratory measurements

We defined quantitative phenotypes based on the laboratory measurements collected during outpatient and inpatient visits. Only quantitative traits with data for populations groups with more than 1,000 individuals were included in the analyses. For the 69 laboratory measurements that passed quality control, we calculated the mean, median, and max values across all visits for each participant then excluded individuals who were extreme outliers (defined as value greater than six standard deviations from the trait mean). The resulting phenotype was normalized using a rank-based inverse-normal transformation prior to analysis.

C) Phenotypes from survey questionnaires

The two surveys (questionnaires) for MVP were designed to augment data contained in each participant's electronic health record and have been described previously (*64*). As with other study activities and all study materials sent to participants, these documents were approved by the VA Central IRB. As participants are enrolled, informed consent and HIPAA authorization forms were scanned by field site staff and sent to the CERC to be checked for accuracy and completeness, and the data were entered in GenISIS. Conceptually, the MVP Baseline Survey was designed to collect information regarding demographics, family pedigree, health status, lifestyle habits, military experience, medical history, family history of specific illnesses, and physical features. The MVP

Lifestyle Survey contains questions from validated instruments in domains selected to provide information on sleep and exercise habits, environmental exposures, dietary habits, and sense of well-being. We selected surveys with either binary responses (yes/no) or quantitative responses (such as height, weight, age at smoking onset, and age at menopause). Quantitative traits underwent the same quality control and data transformation procedures as the laboratory measurements.

This work is covered under VA Central IRB# 10-02: Million Veteran Program (MVP) PI: Gaziano.

2. Genetic association analyses

Optimization of SAIGE

This study aimed to conduct a series of genome-wide association studies (GWAS) using a curated set of 2,068 traits. GWAS were conducted using the linear mixed model method implemented in the Scalable and Accurate Implementation of Generalized Mixed Model algorithm (SAIGE) R package. We optimized SAIGE to run on the US Department of Energy (DOE) Oak Ridge Leadership Computing Facility's (OLCF) Summit infrastructure, which used the power of graphical processing units (GPUs) to speed up the matrix computation involved in building the genetic relationship matrix. This optimization allowed us to distribute the matrix operations evenly among a number of GPUs depending on the size of the genetic relationship matrix. For example, for the largest population group (EUR) we were able to use eight high-memory GPUs, while for EAS, we only needed 1 GPU. The use of GPUs improved the execution time of the analysis, allowing us to quickly and efficiently conduct the GWAS on a large number of phenotypes (**fig. S14**). The GPU-based version of SAIGE is available on GitHUB (*48*) as well as Zenodo (*49*).

Genome-wide Association Analysis (GWAS)

Within each population group, we performed a GWAS for each trait of interest to determine the association with each imputed DNA variant. We used the generalized linear mixed model framework to account for participant relatedness and unbalanced case-control ratios. This was completed using the optimized version of SAIGE described above and on OLCF's Summit supercomputer. Directly genotyped variants were used for step 1 of SAIGE then imputed genetic dosages for step 2. Only variants with an imputation quality >0.3 and MAC >40 within the relevant population group were included in the GWAS. Analyses were adjusted for age, sex, and ten population-specific genetic PCs. All code is available in the GitHUB and Zenodo repositories referenced above.

Post-GWAS Quality Control

GWAS results were filtered using a custom R script based on EasyQC (*65*). Quality control (QC) was implemented to remove variants with missing values for major summary statistics (effect size, standard error, etc.) or with unreasonable values (*P*-values or allele frequencies >1 or <0). Variants that were monomorphic, poorly imputed ($r^2 < 0.3$), or very rare (MAF<0.0001) in only the subset of individuals included in the GWAS were also removed. The post-GWAS QC, and subsequent meta-analysis and QC were completed using OLCF's Andes supercomputing cluster.

Meta-analysis
Multi-population meta-analysis was performed using the fixed-effect, inverse-variance weighted method implemented in GWAMA (*53*). Genomic control was implemented for each population but not applied to the resulting meta-analysis summary statistics. Meta-analysis results were subjected to the same QC procedures as the GWAS results. Imputation quality filters were not implemented; however, an additional filter excluded variants specific to only one population group.

Determination of the number of independent traits tested and the genome-wide significance threshold
We calculated Pearson pairwise correlations for the phenotype residuals derived during step 1 of SAIGE to determine the number of independent traits tested (**fig. S12**). Calculations were made for each population separately and only for the meta-analyzed traits (those that passed phenotype QC in more than one population). PCs were calculated from the correlations, as well as the variance explained by each of the PCs. The number of independent traits for each population was determined by the number of PCs required to explain a variance of 0.99. For the meta-analysis, population-specific residuals were combined before calculating PCs. This approach resulted in 1,083 independent traits. The population-specific and meta-analysis P-value significance thresholds were then determined by dividing the traditional genome-wide threshold of $5x10^{-8}$ by the number of independent traits: $5\times10^{-8}/1083$, resulting in our study-wide significance of 4.6×10^{-11}.

3. Determination of significant loci and independently associated SNPs

Creation of reference panels
LD information is crucial in the post-GWAS analysis of summary statistics (*66*, *67*). MVP participants were genotyped on a custom chip, including a small number of population-specific variants not present in the external reference panel. Estimating LD among SNPs in a genomic region is critical for GWAS analysis, and using a reference panel including populations used for GWAS can improve the accuracy of LD estimation. External reference panels may not be representative of the population being studied, which can lead to biased results. Hence, we created reference panels representing each population group using the individual level genetic data from MVP participants from each population group. Three different reference panels were created to use for different analyses:

A) Panel for identification of independent loci and lead variants

5,000 individuals were selected at random from each of the populations groups included in the study. We then used PLINK 1.9 (*51*) to filter the data, keeping only SNPs with a MAC>20 in each population group. Afterward, we combined all the individual files to create a multi-population reference panel of 20,000 MVP participants. This panel served as a reference for clumping and thresholding to identify independent loci and lead variants from a meta-analysis of summary statistics across multiple populations.

B) Panel for calculating SNP heritability and genetic correlations

The reference panel developed for each population group in (A) was subset to just the SNPs contained in the HapMAP3 SNP list (*68*) provided by the LDSC (*14*) developer team.

C) Panel for fine-mapping

We prepared the population group matched matrices for each significant locus-phenotype combination by filtering 1) the genotype data for variants at the locus and 2) individuals included in the population specific GWAS for the phenotype. We excluded indels and multiallelic sites to avoid potential errors in alignment or strand flipping. After filtering, we calculated all pairwise correlations between the remaining variants using the "--r square --keep-allele-order" options in PLINK v1.9 (*51*) Because PLINK uses hard-called genotypes to calculate LD between variants, but our imputation process generated dosage-based genotypes, it was impossible to calculate correlations between specific pairs of variants using PLINK's default thresholds for hard-call conversion. This occurred both when there were no individuals with convertible genotypes for a given variant and when the sets of individuals with convertible genotypes for distinct variants did not overlap. Because the fine-mapping process cannot tolerate missing correlations in the LD matrix, for any matrix with missing correlations, we iteratively removed the variant with the most missing values until the matrix was complete.

Derivation of independent signals, lead SNPs, genomic risk loci

We processed each set of full GWAS summary statistics dataset using PLINK for clumping and thresholding. Our approach is similar to that previously described in FUMA (*52*) . We used the cross-population and population-specific reference panels described in (A) above to estimated pairwise correlations within each haplotype block to identify independent variants, focusing on those with the lowest P-values and counting the number of independent variants. In the initial clumping phase, we grouped variants that met the study-wide P-value threshold ($P < 4.6\times10^{-11}$) and were independent at an $r^2 < 0.6$, thereby identifying significant, independent variants. The second clumping phase aimed at pinpointing the lead variants, focusing on significant independent variants at an $r^2 < 0.1$. To define genomic risk loci, we first merged linkage disequilibrium (LD) blocks of independent significant variants linked to the same lead variants. We then combined LD blocks that overlapped physically or were within 250Kb. We denoted each risk locus by its lead variant, specifically the one with the lowest P-value in that locus.

SNP heritability and genetic correlations

We used LDSC (*14*) to calculate each trait's SNP heritability and genetic correlation based on the GWAS summary statistics and the reference panel described in (B) above. To generate population-specific LD scores, we followed the steps outlined in this resource (https://github.com/bulik/ldsc/wiki/LD-Score-Estimation-Tutorial). The steps were repeated for each population group to obtain the respective population-specific LD scores.

Locus fine-mapping

Population-specific fine-mapping was completed to create a catalog of putative causal genetic variants at each associated locus, followed by multi-population credible set integration.

Defining loci for fine-mapping

We tiled the genome into adjoining, non-overlapping 250kb segments and, for each phenotype, we identified all segments that contained one or more variants with a meta-analyzed P< 5×10^{-8}. Due to its long-range LD complexities, we excluded the major histocompatibility complex (MHC) (chr6: 25–36 Mb) from the tiling. Next, within each phenotype, we joined all adjacent significant segments into loci and padded the loci with 250kb on both sides. When significant loci overlapped telomere ends, the telomere-side boundaries of the loci were trimmed to coincide with the chromosomal boundaries. Significant loci overlapping the boundaries of the MHC were similarly trimmed. Finally, we mapped only the loci containing at least one variant significant at the study-wide threshold, $P<4.6\times10^{-11}$. These parameters for defining loci differed from those used to identify lead variants due to the exclusion of the MHC and the retaining of loci that contained significant short insertions or deletions but not SNPs. Insertions and deletions were later removed from the variants used for fine-mapping to avoid strand-flip issues though the loci were still mapped. Twenty-one traits had no lead SNPs outside the MHC, and 4 had no significant variants besides insertions and deletions.

Fine-mapping with SuSiE

We statistically fine-mapped the significant phenotype-locus pairs using the Sum of the Single Effects framework (SuSiE) (*18*) with the population-specific summary statistics and computed LD matrices. The LD matrices were matched to each population group and trait to eliminate any potential artifacts that could arise from an LD mismatch between the meta-analyzed GWAS outcomes and the reference panel. We carried out this process independently for each population group, which encompassed AFR, AMR, EAS, and EUR ancestries (as described above in "C) Panel for fine-mapping"). We allowed up to five signals per locus and ran SuSiE through the coloc R package (*69*) with the z-scores as inputs, the "estimate_residual_variance" flag set to true, and the default uniform prior probability of causality. We calculated 95% credible sets for each identified signal representing the fewest number of variants whose posterior inclusion probabilities (PIP) for the signal summed to ≥ 0.95. We discarded credible sets in which the variants had an absolute minimum correlation < 0.1 and/or a minimum $P > 5\times10^{-8}$ for the population group in which the signal was mapped.

We set the maximum number of signals per locus to five instead of the default setting of ten because during testing we observed multiple instances of loci with suspicious credible sets at the higher setting. We tested by calculating the residual association for each signal after removing the effects of the other signals according to the following equation:

$$residual\ association\ for\ signal\ l = \frac{\underline{\rho_l}}{\sqrt{N} * \sigma}$$

Here $\underline{\rho_l}$ corresponds to the expected residuals from the SuSiE algorithm after disregarding the effect of the *l*-th signal (see line 4 of the algorithm for Iterative Bayesian stepwise selection using sufficient or summary statistics) (*18*), N is the sample size, and σ is the standard deviation for the phenotype under the SuSiE model. Under the null model, the residual association ~ $N(0,1)$.

The suspicious credible sets were non-primary signals at loci for phenotypes with suspected highly polygenic architecture including, most notably, height-related traits. These signals generally manifested as single-variant credible sets whose signal-level residual associations appeared as strong outliers in what would have otherwise been loci with the null residual

association for the given signals. Reducing the number of signals mapped per locus from ten to five reduced the frequency of these suspicious credible sets.

Merging signals across population groups

To identify fine-mapped signals in multiple populations, we merged the retained 95% credible sets using an approach first reported by Kanai *et al.*(*20*) . This method involves calculating the PIP-weighted Jaccard similarity indices between all pairs of signals identified for each unique phenotype-locus pair. For each pair of signals, we computed the similarity index as $\Sigma_i \min(x_i,y_i) / \Sigma_i \max(x_i, y_i)$, where x_i and y_i are PIP values for the two signals for each variant *i* that was retained in both populations after making the LD reference panels. We converted each Jaccard similarity index into a distance by taking one minus the similarity index. Then we used the distances to hierarchically cluster the signals using the complete linkage method. We cut the resulting dendrogram tree at the height of 0.9, thereby merging any two credible sets with PIP-weighted Jaccard indices above 0.1 into a single credible set. For any analyses that examined the number of variants in a merged credible set, we defined the variants in each merged approximate credible set as the union of those comprising the component sets.

Computational environment and constraints

We defined loci, constructed LD matrices, fine-mapped, and merged signals on OLCF's supercomputing infrastructure. LD matrix construction and fine-mapping were parallelized across unique combinations of population, phenotype, and locus, and each combination was given 48 hours of wall time and 1 TB of RAM to complete each step. Any analyses that could not be completed within those constraints or that resulted in an error due to SuSiE estimating a negative residual variance were excluded (**table S10**). All phenotype-locus pairs that appeared in at least one population were put through the signal margining process for the completed populations, and any of their merged signals that passed the absolute minimum correlation and significance thresholds were retained in the fine-mapping results.

Suggestive associations and power calculations

To investigate the cause of a large number of signals mapped in a single population group, we looked for signals that had suggestive evidence of an association or lacked the power to detect a suggestive association in one or more unmapped populations. Signals were considered to show suggestive evidence of association in an unmapped population if any variants in the merged approximate credible set had directionally consistent effects between an unmapped and mapped population and population-specific $P<0.001$ in the unmapped population. We also calculated the approximate power to identify associations in the unmapped populations using a two-step approach based on the t-distribution. In the first step, we calculated the critical effect size (absolute beta) for each variant within each unmapped population above which we would detect a suggestive association, given the standard errors and sample sizes for the unmapped population groups. In the second step, we assessed the proportion of an identically shaped t-distribution centered at the "true" effect size for each variant that lay above the critical value or below its negative opposite. For the "true" effect size of a given variant, we assumed the smallest absolute beta across the populations in which the variant was mapped in the credible set. If the proportion outside the critical values was < 80% in any of the unmapped populations and the signal did not show suggestive association, we considered that the signal lacked power.

Effective sample size calculations

To jointly examine the relationship between sample size and the number of fine-mapped signals for quantitative and binary traits, we calculated effective sample sizes for binary traits as the harmonic mean of the cross-population case and control counts (*70*) for the trait. For quantitative traits, we similarly used the cross-population sample sizes.

Comparison of our fine-mapping results with other biobank-based analyses

Compared to other large-scale fine-mapping experiments (*20*, *71*, *72*), our analysis is the broadest and most comprehensive with regard to biggest in terms of the number of phenotypes mapped and signals identified, and only the analysis by Kanai et al. (*20*) is more extensive in terms of samples included. We borrowed much from Kanai et al. (*20*) when designing our approach to fine-mapping, though we chose to deviate from them in several respects, primarily in how we defined loci, in the maximum number of signals we attempted to map at each locus, and in what fine-mapping methods we used.

Regarding locus definition, we wanted to minimize the size of each locus while ensuring that we did not arbitrarily crop the pattern of association and create false positive signals. Though our custom approach resulted in some very large loci, including several greater than 10 MB (for example, at the previously known Duffy/*DARC* locus on chromosome 1 (*73*)), in general, it was successful; it allowed us to fine-map 99% of the loci in all four populations and 99.7% in at least one population. The key shortcoming of our locus-defining scheme is that it can create overlapping loci, which can and did result in double-counted signals; 115 signals (0.2% of signals) were double-counted. As far as we know, Kanai et al. (*20*) did not provide fine-mapping completion statistics for their approach, however, their locus-defining scheme should have excluded the possibility of double-counted signals.

At a summary level, our fine-mapping results were broadly similar to those obtained by Kanai et al. (*20*) except that we obtained more-precise credible sets. Across the three biobanks they analyzed, between 34 and 37% of the signals Kanai et al. (*20*) mapped contained five or fewer variants. At the same time, 54% of our merged credible sets were fine-mapped to the same precision. We observed an enrichment of rarer variants among more precisely mapped credible sets (**fig. S4**) that could explain this precision difference. Though Kanai et al. (*20*) applied inconsistent frequency-based variant filters across their cohorts, in general, it appears that we retained more rare variants in our analysis. Since we observed important coding (**Fig. 3D**) and noncoding (**fig. S4**) functional enrichments among our precisely mapped credible sets, the inclusion of these rarer variants was robust and an advantage of our approach. Apart from allele frequencies, differences in which phenotypes were fine-mapped could also explain the precision gap, though this possibility is less likely. We examined the precision of our fine-mapped signals across the five phenotype categories we examined, and even in the lowest category we fine-mapped 41% of the signals to five or fewer variants (**fig. S3**). Subcategory-level differences in precision rates could still account for the precision difference, as could differences in LD matrix construction, population composition, and fine-mapping methods. However, more work would be needed to investigate these possibilities.

4. Locus annotation

Functional annotations and coding enrichment

We used the Variant Effect Predictor (VEP) (*54*) to determine the most severe functional consequence of each associated and fine-mapped variant. The 39 annotations assigned by VEP were grouped into ten larger categories, including three comprising coding variants: splice/start/stop gain/loss, missense, and synonymous (**table S20**). For each variant annotated as non-coding, we used RegulomeDB v2.2 (*55*) to assign a probability score and category indicating the variant's probability of being functional. To assess enrichment in coding variation and higher RegulomeDB scores across different categories of variants, we respectively used Fisher's exact and Wilcoxon rank sum tests.

Derived and ancestral allele identification for high-confidence, fine-mapped variants

We determined the identities of the ancestral and derived alleles for all variants that were fine-mapped with high confidence in any population (PIP > 0.95) by referencing the parsimony-derived ancestral allele identities in the 1000G Phase III variant call format (vcf) files (*59*) Given our imputation scheme, 6,150 of 6,318 (97.3%) of the high-confidence variants were included in the 1000G callsets. Of the included variants, only those with consistently annotated ancestral alleles across the inferred orangutan-chimp-human progenitor, inferred chimp-human progenitor, and chimp lineages were retained for analyses involving ancestral/derived alleles. These remaining variants comprise 12,613 high-confidence SNP-phenotype pairs.

Defining known and previously unidentified associations

We compared the genomic locus and fine-mapped variants and their proxies (with an $r^2 > 0.1$) to determine their previously reported association in both the NHGRI EMBL GWAS Catalog (*11*) and Open Targets Genetics database (*12*).These databases employ Experimental Factor Ontology (EFO) terms as the principal vocabulary for standardizing traits and phenotypes, prompting us to initially map all traits analyzed in our study to EFO terms. We implemented semi-automated processes to map specific trait descriptions to EFO terms to bridge the gap between disparate data sources. This process involves processing various mapping files and EFO terms from the GWAS Catalog and Open Targets Genetics, which provide phecode, labs, and vitals to EFO term mappings. Initially, we map these phecodes to their corresponding EFO terms using pre-existing mappings. For traits not categorized as phecodes, we employed a "text descriptive fuzzy mapping" technique to assign these remaining traits to EFO terms. Subsequently, we carried out a manual review of trait to EFO term mappings since there were instances where different EFO terms were used for the same term by GWAS Catalog and Open Targets Genetics. Upon a thorough review the final table of traits and their corresponding EFO terms was created for cross-reference with the GWAS Catalog and Open Targets Genetics.

For each trait, we searched for any variants at a given locus identified in our study as having an association in the GWAS Catalog and Open Targets Genetics with same trait. For fine-mapped variants, we identify tag variants linked to them (with an $r^2 > 0.1$ and within a 500kb window). We then cross-reference the fine-mapped variant and tag variants in the GWAS Catalog and Open Targets Genetics to verify their association with the trait of interest. We classify each association into one of three categories: a) Known Association: either the variant in genomic loci or fine-mapped variant or tag variants are already associated with the same trait in the GWAS Catalog or Open Targets Genetics. b) Previously Unidentified Association with Known Signal:

the fine-mapped variant or tag variants are not associated with the same trait but have known associations with other traits. c) Previously Unidentified Signal: We use this category when the fine-mapped variant or tag variants do not have any association with traits listed in the GWAS Catalog or Open Targets Genetics.

Cross-population comparisons of credible set sizes

We compared the number of variants in the population-specific credible sets for signals successfully merged across more than one population (excluding the EAS population, as it lacked sufficient power). We used a paired Wilcoxon sign-rank test to identify significant differences in the credible set sizes for signals mapped in AFR/AMR, AFR/EUR, and AMR/EUR populations.

Down-sampling Analysis

In order to investigate the power difference between EUR and AFR due to genetic architecture rather than sample size, we down-sampled the EUR population to match AFR then reran the GWAS for this down-sampled EUR population (EUR_{dsAFR}).

EUR and AFR participants were allocated to bins matched on biological sex and age (5-year bins). One EUR participant was randomly selected for each AFR participant in each bin, in order to create a subsample with similar sex and age distribution. Minor allele frequencies and imputation qualities were recalculated for EUR_{dsAFR} using plink2. GWAS was completed for EUR_{dsAFR} using the same SAIGE methods as the main analysis, then the results underwent the same quality control procedures using the recalculated EUR_{dsAFR} minor allele frequencies and imputation qualities.

The trait-loci combinations that were previously found to have 1 or more signal identified in both the AFR and full EUR populations were fine-mapped using results from EUR_{dsAFR}. We used Jaccard similarity indices calculated on the signal-level PIPs to merge the signals identified into EUR_{dsAFR} and the full AFR population for each of these trait-loci. We analyzed the differences in credible set sizes for any signals that were identified in both populations.

Putative Gene Nomination for Fine-Mapped Variants

We used a two-step approach to nominate putative causal genes mediating the association of fine-mapped variants at GWAS loci. In the first step of our nomination scheme we used bedr (*74*) to intersect fine-mapped variants with exons of protein-coding genes annotated in GENCODE release 19 (*75*). We then linked all fine-mapped variants within a protein-coding exon to the gene in which they fell, provided the variants were characterized as either missense or splice/start/stop gain/loss by VEP (**table S20**). We used VEP (*54*) to identify and remove synonymous variants, those in 5'/3' untranslated regions, and a small minority falling in occasionally-retained introns.

For the second step of our nomination approach, we leveraged the predictions of the Activity-by-Contact Model (ABC) (*33*) previously applied in 131 human cell types and tissues (*76*) ABC determines active regulatory elements and the genes they regulate via two methods. In the first, active promoter regions of expressed genes are linked to their respective genes. In the second, ABC links active distal enhancers to expressed genes whose promoters were observed to physically interact with the enhancers by average Hi-C contacts across 10 human datasets. In

both cases activity is determined on a cell-type basis via the geometric mean of chromatin accessibility, determined from either ATAC-seq (assay for transposase-accessible chromatin using sequencing) or DNase-seq (DNase I hypersensitive site sequencing), and H3K27ac, determined via ChIP-seq (chromatin immunoprecipitation sequencing).

To use ABC to nominate genes for synonymous and non-coding variants, we first downloaded the predicted regulatory connections in 131 cell types from the Engreitz Lab website (*77*). We then used bedr (*74*) to intersect the active promoter and enhancer regions with all remaining fine-mapped variants not linked to a gene via non-synonymous coding effects. Seeking to limit erroneous nominations, we only used regulatory elements with an ABC score > 0.1 and restricted both steps of our approach to trait-variant pairs fine-mapped with PIP >0.01 in at least one population.

We also observed that for quantitative traits we frequently fine-mapped the same variant for all three versions of the trait (mean, minimum, and maximum value across all visits). To prevent inflating the number of nominated genes we collapsed the three versions of each quantitative trait into a single trait for the purpose of reporting the number of nominations and reviewing the genes and pathways linked to any one particular trait.

KEGG Pathway Enrichments

We used the clusterProfiler R package (*78*) to identify significantly enriched KEGG pathways for each trait via over-representation analysis (*79*). We corrected the results for multiple testing jointly across all traits using the Benjamini-Hochberg procedure (*80*).

Regressions of Gene-Level Pleiotropy on GO Terms

We sought to use our cross-trait gene nominations to assess the biological factors that influence gene pleiotropy. To do so, we first identified the number of independent (uncorrelated) traits associated with each nominated gene using a procedure similar to the one employed for the analysis of pleiotropic variant annotations. As before, a trait (trait #1) was selected for each gene based on the largest PIP value across all variants that implicated the trait in any population in which the variant was fine-mapped. If there was a tie, we selected trait #1 at random from among all traits with the maximum PIP. Next, all associated traits with an absolute phenotypic correlation coefficient > 0.2 with trait #1 were flagged as correlated and removed from further consideration. We iterated over these steps until only uncorrelated traits remained for each gene. Since we necessarily calculated inter-trait genetic correlations with the uncollapsed quantitative traits, we ran this selection process on the uncollapsed traits. Doing so did not inflate the number of independent traits per gene as the three versions of each quantitative trait are strongly correlated. Additionally, a random seed was set prior to running this procedure to ensure reproducibility.

We next used BioMart (*81*) to identify for each of the 13,287 biological process, cellular component, and molecular function gene ontology (GO) terms which nominated genes were categorized with the annotation. Separately for each GO term, we then regressed the number of independent traits per gene onto the binary variable indicating whether a gene was part of the GO term. We used a Poisson generalized linear model for each regression as the number of independent traits is a discrete count variable. We extracted the regression coefficient and its P-

value for each of the regressions and corrected the p-values using the Benjamini-Hochberg procedure. To detect related categories of GO-terms and visualize the results of this analysis, we used GO-Figure (*82*). GO-Figure clustered the significant terms (adj. $P < 0.05$) via semantic similarities (*83*) that can be thought of as measuring the minimum number of edges required to connect two terms on the GO graph weighted by the specificity of the terms. This method assigns a representative term as a label to each cluster that aims to prioritize more significant terms and parent terms over their children.

As a final analysis of the nominated genes, we regressed the number of independent traits per gene onto the number of GO terms annotated for each gene using a Poisson generalized linear model.

Identifying variant-trait associations specific to the non-EUR populations

We focused on characterizing previously unidentified SNPs in the non-EUR groups fine-mapped to a trait by identifying variants mapped with high confidence (PIP > 0.95) in the non-EUR groups and either unmapped or mapped with low confidence in the EUR population (**table S12**). We further restricted to coding variants as defined by VEP (*54*) and with a MAF > 0.05 in each of the non-EUR populations in the main results.

5. Heterogeneity Analysis

Screening for heterogeneous effects across populations was performed on variants fine-mapped to a trait, specifically for phecodes only, from any population with a MAF > 0.05. The quantitative traits were not tested for heterogeneity due to the potential discrepancy in their scales across different population groups. The variant-trait pair must have been fine-mapped with high confidence (PIP > 0.95) in both groups being compared (AFR vs. EUR, AMR vs. EUR; the sample size for EAS was underpowered for the multiple testing among ultra-high-dimensional hypotheses) with MAF > 0.05. To adequately control for the false discovery rate (FDR), the heterogeneity analysis performs an adaptive multiple testing procedure on the heterogeneity effect (*84*) on the full set of variant-phenotype pairs. The adaptive heterogeneity multiple testing procedure improves power by reweighting the heterogeneity test statistics according to the level of evidence for the presence of an overall mean effect. In the broad heterogeneity screen, we examined all fine-mapped variants with MAF>0.05 in both groups being compared, such as AFR vs. EUR and AMR vs. EUR, and required overlapping credible sets. This allowed us to potentially detect variants with effects only present in the population or with small effect sizes that may be underpowered for AFR or AMR groups. The primary heterogeneity analysis further required that the variant be amino acid changing with PIP > 0.95 in both populations and that the fine-mapping result showed the same variant-phenotype pair in both populations.

We developed a statistical testing framework detailed below for assessing the heterogeneity of genetic associations with multiple phenotypes across different population groups with a goal to simultaneously test whether the effects of genetic variants on the phenotypes are homogeneous across all groups or if heterogeneity is present in at least one group. The framework used various statistical models, including linear and logistic regression, to model the relationships between genetic variants, adjustment covariates, and phenotypes.

Problem Setup

Let J be the number of population groups considered for testing heterogeneity. For each subject i from each group j, we observe a K-dimensional vector of outcomes $Y_{i,j} = \left(Y_{i,j,1}, Y_{i,j,2}, \dots, Y_{i,j,K}\right)^T$, a vector of adjustment covariates $X_{i,j}$ with demographic variables, and an L-dimensional vector of SNPs $A_{i,j} = \left(A_{i,j,1}, A_{i,j,2}, \dots, A_{i,j,L}\right)^T$. Within each population group j, the outcome $Y_{i,j,k}$ is modeled against each SNP $A_{i,j,l}$ and adjustment covariates $X_{i,j}$. For continuous outcomes, we consider the linear model:

$$Y_{i,j,k} = \beta_{j,l,k} A_{i,j,l} + \gamma_{j,l,k}^T X_{i,j} + \epsilon_{i,j,k},\ where\ \ \epsilon_{i,j,k} \sim N\left(0, \sigma_{j,k}^2\right)$$

while for binary outcomes, we use the logistic model:

$$P\left(Y_{i,j,k} = 1 \mid A_{i,j,l}, X_{i,j}\right) = expit\left(\beta_{j,l,k} A_{i,j,l} + \gamma_{j,l,k}^T X_{i,j}\right),$$

where $expit(a) = e^a/(1 + e^a)$. For each pair of phenotype k and SNP l, let $\beta_{j,l,k} = \mu_{l,k} + \alpha_{j,l,k}$ where $\mu_{l,k}$ denotes the mean association between outcome k and SNP l across all population groups and $\alpha_{j,l,k}$ reflects the heterogeneity in effect from group j. Define the true non-null sets $S_\mu = \{(l,k): \mu_{lk} \neq 0\}$ and $S_\alpha = \{(l,k): \alpha_{j,lk} \neq 0 \text{ for some } j\}$. We assume that approximately: $S_\alpha \subseteq S_\mu$ or S_α and S_μ are very similar. For $(l,k) \in \{1, \dots, L\} \times \{1, \dots, K\}$, we are interested in simultaneously testing:

$$\alpha_{1,lk} = \cdots = \alpha_{J,lk} = 0\ v.s.\ \alpha_{j,lk} \neq 0\ for\ some\ j \in \{1, \dots, J\}.$$

with the false discovery rate (FDR) controlled below some level η (e.g., $\eta = 0.1$):

$$E\left[\frac{\#\ of\ false\ discovery}{Total\ \#\ of\ discovery}\right] \leq \eta.$$

Test Statistics

We first construct the effect estimator $\hat{\beta}_{j,l,k}$ for each phenotype k against each SNP l on each group j and estimate its asymptotic variance $\hat{\sigma}_{j,l,k}$ using the standard score test approach. Then we introduce the mean effect auxiliary statistic constructed as the inverse-variance weighted average across population groups:

$$\hat{\mu}_{l,k} = \frac{\sum_{j=1}^{J} \left(\hat{\sigma}_{j,l,k}\right)^{-2} \hat{\beta}_{j,l,k}}{\sum_{j=1}^{J} \left(\hat{\sigma}_{j,l,k}\right)^{-2}},\ which\ follows\ N\left(0, \frac{1}{\sum_{j=1}^{J} \left(\hat{\sigma}_{j,l,k}\right)^{-2}}\right)\ when\ \beta_{1,l,k} = \cdots = \beta_{J,l,k} = 0,$$

and the heterogeneity test statistic as a quadratic form of the estimated heterogeneity effects:

$$\hat{T}_{l,k} = \sum_{j=1}^{J} \left(\hat{\beta}_{j,l,k} - J^{-1} \sum_{j'=1}^{J} \hat{\beta}_{j',l,k}\right)^2,\ which\ follows\ \sum_{j=1}^{J} \lambda_j \chi_{1(j)}^2\ when\ \beta_{1,l,k} = \cdots = \beta_{J,l,k}.$$

Here $\chi^2_{1(j)}, \ldots, \chi^2_{1(J)}$ are J mutually independent chi-square random variables with degree of freedom 1 , and λ_j 's are estimated by calculating the eigenvalues of the $J \times J$ empirical covariance matrix:

$$\hat{\Sigma}_{l,k} = \frac{\sum_j \quad \left(\hat{\sigma}_{j,l,k}\right)^2}{J^2} 1 - \left[diag\left(\frac{\left(\hat{\sigma}_{j,l,k}\right)^2}{J}\right)\right]_{j=1}^{J} 1_J - 1_J \left[diag\left(\frac{\left(\hat{\sigma}_{j,l,k}\right)^2}{J}\right)\right]_{j=1}^{J} + \left[diag\left(\left(\hat{\sigma}_{j,l,k}\right)^2\right)\right]_{j=1}^{J},$$

where 1_J is a $J \times J$ matrix of all ones. The p-value of $\hat{T}_{l,k}$, denoted as $\hat{p}_{\alpha,l,k}$, characterizes significance of the across-group heterogeneity of the association between phenotype k and SNP l, and is calculated using CompQuadForm package in (5). Meanwhile, we extract the p-value of the mean effect statistics $\hat{\mu}_{l,k}$, denoted as $\hat{p}_{\mu,k,l}$, as a guiding auxiliary information to enhance the testing power. It is not hard to show that $\hat{\mu}_{l,k}$ is asymptotically independent with $\hat{\beta}_{j,l,k} - J^{-1}\sum_{j'=1}^{J}\hat{\beta}_{j',l,k}$ for every $j = 1,2,\ldots,J$, and, thus, is asymptotically independent with $\hat{T}_{l,k}$. This grants the validity of using each $\hat{p}_{\mu,k,l}$ as a side information to re-weight $\hat{p}_{\alpha,k,l}$, which is an important step to be introduced in the next section.

Construction of Weights

We aim at taking $\hat{p}_{\mu,l,k}$ as an auxiliary information to assign weights to $\hat{p}_{\alpha,l,k}$, based upon the prior assumption that the non-null set of heterogeneity S_α is close to that of the mean effects S_μ. Asymptotic independence between $\hat{p}_{\mu,l,k}$ and $\hat{p}_{\alpha,l,k}$ warrants the validity of this strategy and the large effective sample size for the overall effect $\hat{p}_{\mu,l,k}$ makes it a precise enough information to enhance the power of the heterogeneity effects testing. Inspired by recent literature of adaptive multiple testing (*75*, *62*) that leverages auxiliary information to improve the power over the standard Benjamini Hochberg (BH) procedure (*62*) , we propose the following steps to derive $\hat{p}_{\mu,l,k}$ into proper weights of the testing p-values $\hat{p}_{\alpha,l,k}$:

1 Calculate $Z_{\alpha,l,k} = I\left(\hat{p}_{\alpha,l,k} < \tau_\alpha\right)$ where τ_α is some pre-specified cutoff parameter. Practically, one can either fix τ_α as some small value like 10^{-5} or specify it empirically, e.g. choosing τ_α as the p-value cutoff returned from the BH procedure on $\{\hat{p}_{\alpha,k} : k = 1,2,\ldots,K\}$ with level 0.5.

2 Implement a logistic regression on

$$Z_{\alpha,l,k} \sim a \cdot m \quad \{logit\left(1 - \hat{p}_{\mu,l,k}\right), 12\} + b_k,$$

where $logit(x) = log\{x/(1-x)\}, a$ is a regression coefficient and b_k represents coefficient for the phenotype k. Since $\hat{p}_{\mu,l,k}$ could be extremely close to zero, we use logit-transformation and threshold on $\hat{p}_{\mu,l,k}$ to make the weighting model more stable. Let the fitted coefficients be $\hat{a}$ and $\hat{b}_k$ for $k = 1,2,\ldots,K$. We set the weight for $\hat{p}_{\alpha,l,k}$ as

$$\hat{\pi}_{l,k} = m \quad \left\{1 - \frac{1 - exp\{\hat{a} \cdot m \quad \{logit\left(1 - \hat{p}_{\mu,l,k}\right), 12\} + \hat{b}_k\}}{1 - \tau_\alpha}, 0\right\}.$$

3 Standardize $\hat{\pi}_{l,k}$ and obtain the final weights $\hat{q}_{l,k}$ through: $\hat{q}_{l,k} = KL\left(\sum_{k=1}^{K}\sum_{l=1}^{L}\hat{\pi}_{l,k}\right)^{-1}\hat{\pi}_{l,k}$.

Construction of $\hat{\pi}_{l,k}$ is inspired by the optimal bayesian decision rule used in Cai et al. (*74*). Since we do not observe the true set with heterogeneity effects S_{α}, we use $Z_{\alpha,l,k} = I\left(\hat{p}_{\alpha,l,k} < \tau_{\alpha}\right)$ as a surrogate for the presence of heterogeneity on phenotype k and SNP l, and perform regression on it against the auxiliary information to derive the weight $\hat{\pi}_{l,k}$. Since $Z_{\alpha,l,k}$ depends on the testing p-value $\hat{p}_{\alpha,l,k}$, the final FDR control procedure described below is modified correspondingly to ensure validity. Our third step of standardizing $\hat{\pi}_{l,k}$ is also used to protect the validity and FDR control.

Adaptive FDR Control
Finally, we weight and adjust the heterogeneity testing p-values as $\hat{p}_{\alpha,l,k}^{q} = min\left\{\hat{p}_{\alpha,l,k}/\hat{q}_{l,k}, 1\right\}$ for each pair of l and k, and implement the following algorithm for discovery with FDR control of level η.

Computational Dimensionality Reduction
Considering the ultra-high-dimensionality of the genetic variants and phenotypes, we use a simple strategy that can reduce the burden of computation and storage while maintaining little loss on power.

At the Algorithm 1 Adaptive multiple testing with FDR level η.

1: Find $\hat{r} = max\{r \geq 1: \hat{p}_{\alpha,l,k}^{q} \leq \{r\eta/(KL)\} \wedge \tau_{\alpha}$ for at least r many $\hat{p}_{\alpha,l,k}^{q}\}$;

2: Reject null hypothesis with $\hat{p}_{\alpha,l,k}^{q} \leq \{\hat{r}\eta/(KL)\} \wedge \tau_{\alpha}$ for a total of $\hat{r}$ rejections.

beginning, we still use score test to derive the beta coefficient $\hat{\beta}_{j,l,k}$, its asymptotic variance $\hat{\sigma}_{j,l,k}$, and p-value $\hat{p}_{\beta,j,l,k}$ for each j, l and k. Then we introduce a modified multiple testing procedure requiring less resource on computation and storage in Algorithm 2.

Algorithm 2 Computational Dimensionality Reduction.

1: For each pair l and k, if $min\left\{\hat{p}_{\beta,j,l,k}: j = 1,2, \dots, J\right\} > \tau_{\beta}$, skip the steps of constructing $\hat{\mu}_{l,k}, \hat{T}_{l,k}$ and simply set $\hat{p}_{\mu,l,k} = \hat{p}_{\alpha,l,k} = 1$; otherwise, computing $\hat{\mu}_{l,k}, \hat{T}_{l,k}, \hat{p}_{\mu,l,k}$ and $\hat{p}_{\alpha,l,k}$ using the same way described above.

2: Implement the same weight construction procedure with only the pairs satisfying $\hat{p}_{\mu,l,k}, \hat{p}_{\alpha,l,k} \neq 1$;

3: Implement Algorithm 1 on the union set of re-weighted p-values and the p-values set as 1 in the first step, for multiple testing with FDR control.

In Algorithm 2, τ_β is some pre-specified small threshold parameters set as 10^{-6} in our case. The smaller τ_β is, the less computation and storage resource Algorithm 2 will require but there will be a high risk of losing power if τ_β is excessively small. Inspired by Liu et al. (*76*), Algorithm 2 preserves validity because it only makes conservative changes by setting many heterogeneity p-values as 1 and still adjusts for the total number of hypotheses as KL in the weighting and FDR control procedures. Meanwhile, the screening procedure, Step 1 in Algorithm 2, may not impact power since it only excludes pairs of SNP and phenotype with apparently non-significant heterogeneity effects.

Supplementary Text

Contributions and Acknowledgments

MVP Program Office

- Sumitra Muralidhar, Ph.D., Program Director
- Jennifer Moser, Ph.D., Associate Director, Scientific Programs
- Jennifer E. Deen, B.S., Associate Director, Cohort & Public Relations

MVP Executive Committee

- Co-Chair: Philip S. Tsao, Ph.D.
- Co-Chair: Sumitra Muralidhar, Ph.D.
- J. Michael Gaziano, M.D., M.P.H.
- Elizabeth Hauser, Ph.D.
- Amy Kilbourne, Ph.D., M.P.H.
- Shiuh-Wen Luoh, M.D., Ph.D.
- Michael Matheny, M.D., M.S., M.P.H.
- Dave Oslin, M.D.

MVP Co-Principal Investigators

- J. Michael Gaziano, M.D., M.P.H.
- Philip S. Tsao, Ph.D.

MVP Core Operations

- Lori Churby, B.S., Director, MVP Regulatory Affairs
- Stacey B. Whitbourne, Ph.D., Director, MVP Cohort Management
- Jessica V. Brewer, M.P.H., Director, MVP Recruitment & Enrollment
- Shahpoor (Alex) Shayan, M.S., Director, MVP Recruitment and Enrollment Informatics
- Luis E. Selva, Ph.D., Executive Director, MVP Biorepositories
- Saiju Pyarajan Ph.D., Director, Data and Computational Sciences
- Kelly Cho, M.P.H, Ph.D., Director, MVP Phenomics Data Core
- Scott L. DuVall, Ph.D., Director, VA Informatics and Computing Infrastructure (VINCI)
- Mary T. Brophy M.D., M.P.H., Director, VA Central Biorepository
- MVP Coordinating Centers
    - MVP Coordinating Center, Boston - J. Michael Gaziano, M.D., M.P.H.

- MVP Coordinating Center, Palo Alto – Philip S. Tsao, Ph.D.
- MVP Information Center, Canandaigua – Brady Stephens, M.S.
- Cooperative Studies Program Clinical Research Pharmacy Coordinating Center, Albuquerque – Todd Connor, Pharm.D.; Dean P. Argyres, B.S., M.S.

MVP Publications and Presentations Committee

- Co-Chair: Themistocles L. Assimes, M.D., Ph. D
- Co-Chair: Adriana Hung, M.D.; M.P.H
- Co-Chair: Henry Kranzler, M.D.

MVP Local Site Investigators

- Samuel Aguayo, M.D., Phoenix VA Health Care System
- Sunil Ahuja, M.D., South Texas Veterans Health Care System
- Kathrina Alexander, M.D., Veterans Health Care System of the Ozarks
- Xiao M. Androulakis, M.D., Columbia VA Health Care System
- Prakash Balasubramanian, M.D., William S. Middleton Memorial Veterans Hospital
- Zuhair Ballas, M.D., Iowa City VA Health Care System
- Jean Beckham, Ph.D., Durham VA Medical Center
- Sujata Bhushan, M.D., VA North Texas Health Care System
- Edward Boyko, M.D., VA Puget Sound Health Care System
- David Cohen, M.D., Portland VA Medical Center
- Louis Dellitalia, M.D., Birmingham VA Medical Center
- L. Christine Faulk, M.D., Robert J. Dole VA Medical Center
- Joseph Fayad, M.D., VA Southern Nevada Healthcare System
- Daryl Fujii, Ph.D., VA Pacific Islands Health Care System
- Saib Gappy, M.D., John D. Dingell VA Medical Center
- Frank Gesek, Ph.D., White River Junction VA Medical Center
- Jennifer Greco, M.D., Sioux Falls VA Health Care System
- Michael Godschalk, M.D., Richmond VA Medical Center
- Todd W. Gress, M.D., Ph.D., Hershel “Woody” Williams VA Medical Center
- Samir Gupta, M.D., M.S.C.S., VA San Diego Healthcare System
- Salvador Gutierrez, M.D., Edward Hines, Jr. VA Medical Center
- John Harley, M.D., Ph.D., Cincinnati VA Medical Center
- Kimberly Hammer, Ph.D., Fargo VA Health Care System
- Mark Hamner, M.D., Ralph H. Johnson VA Medical Center
- Adriana Hung, M.D., M.P.H., VA Tennessee Valley Healthcare System
- Robin Hurley, M.D., W.G. (Bill) Hefner VA Medical Center
- Pran Iruvanti, D.O., Ph.D., Hampton VA Medical Center
- Frank Jacono, M.D., VA Northeast Ohio Healthcare System
- Darshana Jhala, M.D., Philadelphia VA Medical Center
- Scott Kinlay, M.B.B.S., Ph.D., VA Boston Healthcare System
- Jon Klein, M.D., Ph.D., Louisville VA Medical Center
- Michael Landry, Ph.D., Southeast Louisiana Veterans Health Care System
- Peter Liang, M.D., M.P.H., VA New York Harbor Healthcare System
- Suthat Liangpunsakul, M.D., M.P.H., Richard Roudebush VA Medical Center
- Jack Lichy, M.D., Ph.D., Washington DC VA Medical Center

- C. Scott Mahan, M.D., Charles George VA Medical Center
- Ronnie Marrache, M.D., VA Maine Healthcare System
- Stephen Mastorides, M.D., James A. Haley Veterans' Hospital
- Elisabeth Mates M.D., Ph.D., VA Sierra Nevada Health Care System
- Kristin Mattocks, Ph.D., M.P.H., Central Western Massachusetts Healthcare System
- Paul Meyer, M.D., Ph.D., Southern Arizona VA Health Care System
- Jonathan Moorman, M.D., Ph.D., James H. Quillen VA Medical Center
- Timothy Morgan, M.D., VA Long Beach Healthcare System
- Maureen Murdoch, M.D., M.P.H., Minneapolis VA Health Care System
- James Norton, Ph.D., VA Health Care Upstate New York
- Olaoluwa Okusaga, M.D., Michael E. DeBakey VA Medical Center
- Kris Ann Oursler, M.D., Salem VA Medical Center
- Ana Palacio, M.D., M.P.H., Miami VA Health Care System
- Samuel Poon, M.D., Manchester VA Medical Center
- Emily Potter, Pharm.D., VA Eastern Kansas Health Care System
- Michael Rauchman, M.D., St. Louis VA Health Care System
- Richard Servatius, Ph.D., Syracuse VA Medical Center
- Satish Sharma, M.D., Providence VA Medical Center
- River Smith, Ph.D., Eastern Oklahoma VA Health Care System
- Peruvemba Sriram, M.D., N. FL/S. GA Veterans Health System
- Patrick Strollo, Jr., M.D., VA Pittsburgh Health Care System
- Neeraj Tandon, M.D., Overton Brooks VA Medical Center
- Philip Tsao, Ph.D., VA Palo Alto Health Care System
- Gerardo Villareal, M.D., New Mexico VA Health Care System
- Agnes Wallbom, M.D., M.S., VA Greater Los Angeles Health Care System
- Jessica Walsh, M.D., VA Salt Lake City Health Care System
- John Wells, Ph.D., Edith Nourse Rogers Memorial Veterans Hospital
- Jeffrey Whittle, M.D., M.P.H., Clement J. Zablocki VA Medical Center
- Mary Whooley, M.D., San Francisco VA Health Care System
- Allison E. Williams, N.D., Ph.D., R.N, Bay Pines VA Healthcare System
- Peter Wilson, M.D., Atlanta VA Medical Center
- Junzhe Xu, M.D., VA Western New York Healthcare System
- Shing Shing Yeh, Ph.D., M.D., Northport VA Medical Center

External Resources

Data Resources

- CIPHER - https://phenomics.va.ornl.gov/
- Imputation Reference Panels - https://imputation.sanger.ac.uk/?about=1#referencepanels
- HapMap3 SNP List - https://console.cloud.google.com/storage/browser/_details/broad-alkesgroup-public-requester-pays/LDSCORE/w_hm3.snplist.bz2;tab=live_object
- RegulomeDB v2.2 Scores - https://regulomedb.org/
- 1000G VCFs - https://ftp.1000genomes.ebi.ac.uk/vol1/ftp/release/20130502/
- GWAS Catalog - https://www.ebi.ac.uk/gwas/docs/file-downloads
- Open Targets Genetics - https://genetics-docs.opentargets.org/data-access/data-download

- Gencode Release 19 Comprehensive Gene Set - https://ftp.ebi.ac.uk/pub/databases/gencode/Gencode_human/release_19/gencode.v19.annotation.gtf.gz
- ABC Results from 131 Cell Types - https://mitra.stanford.edu/engreitz/oak/public/Nasser2021/AllPredictions.AvgHiC.ABC0.015.minus150.ForABCPaperV3.txt.gz

Code Resources

- EIGENSOFT - http://www.hsph.harvard.edu/alkes-price/software/.
- EasyQC - http://www.genepi-regensburg.de/easyqc/
- GWAMA - https://www.geenivaramu.ee/tools/GWAMA_v2.2.2.zip
- LDSC - https://github.com/bulik/ldsc
- Plink v1.9 - https://zzz.bwh.harvard.edu/plink/
- Plink v2.0 - https://www.cog-genomics.org/plink/2.0/
- coloc - https://github.com/chr1swallace/coloc
- VEP - https://grch37.ensembl.org/info/docs/tools/vep/index.html
- biomaRt - https://bioconductor.org/packages/release/bioc/html/biomaRt.html
- clusterProfiler - https://bioconductor.org/packages/release/bioc/html/clusterProfiler.html
- GO-Figure - https://gitlab.com/evogenlab/GO-Figure

Figures

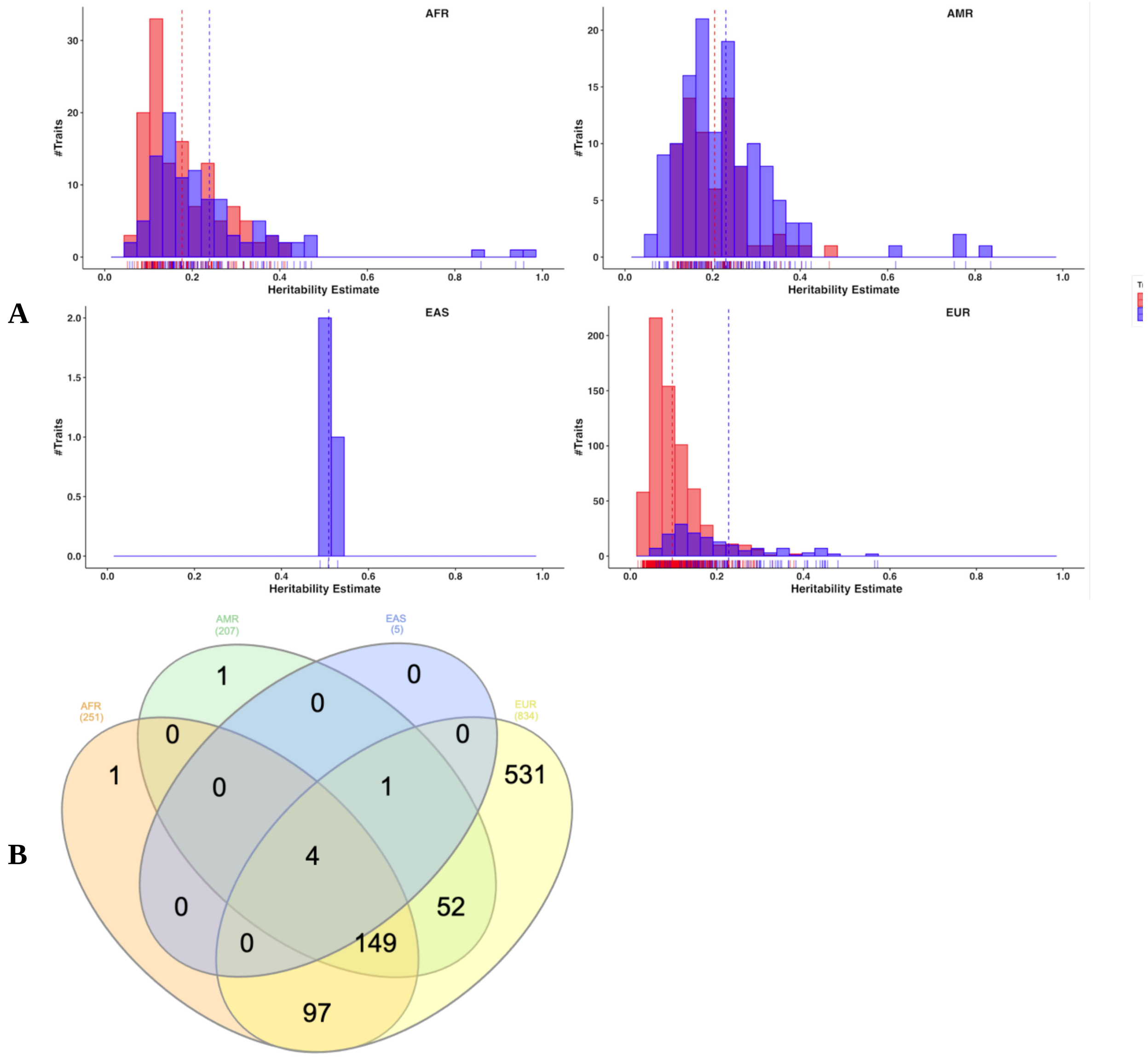

Figure S1. Heritability estimates of traits across four populations groups. (A) Histograms depicting the heritability estimates for MVP genetically-inferred ancestry population groups: African (AFR), Admixed American (AMR), East Asian (EAS), and European (EUR). (B) Venn diagram showing the overlap between significant heritable traits ($P < 9 \times 10^{-6}$) across the same four population groups.

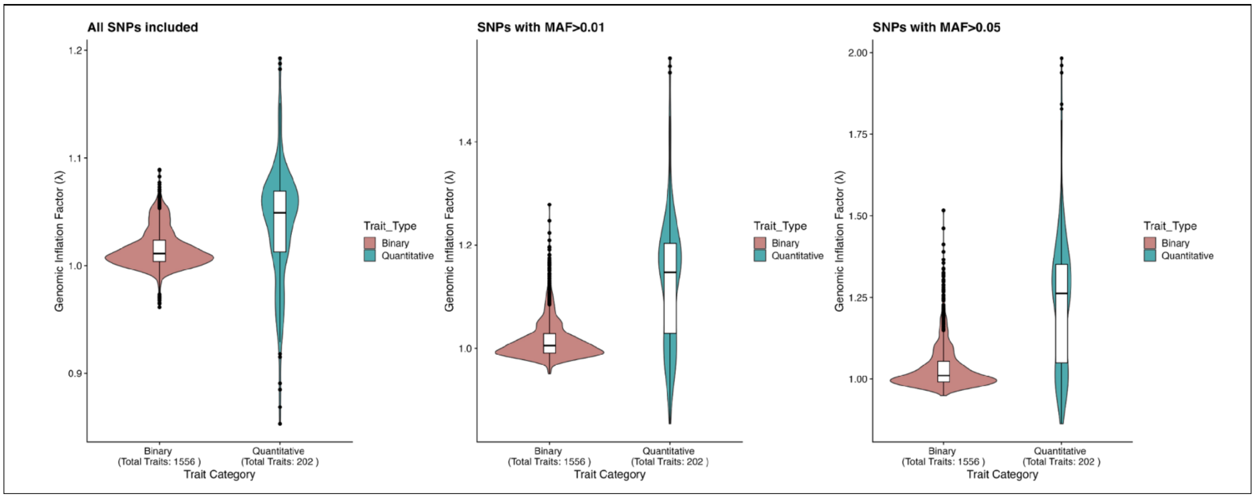

Figure S2. Genomic inflation factors for meta-analyzed traits. Violin plot of meta-analysis genomic inflation factor (lambda) for binary (brown) and quantitative (blue) traits. Lambda was calculated using all SNPs (left), SNPs with a minor allele frequency>0.01 (center), and SNPs with a MAF>0.05 (right).

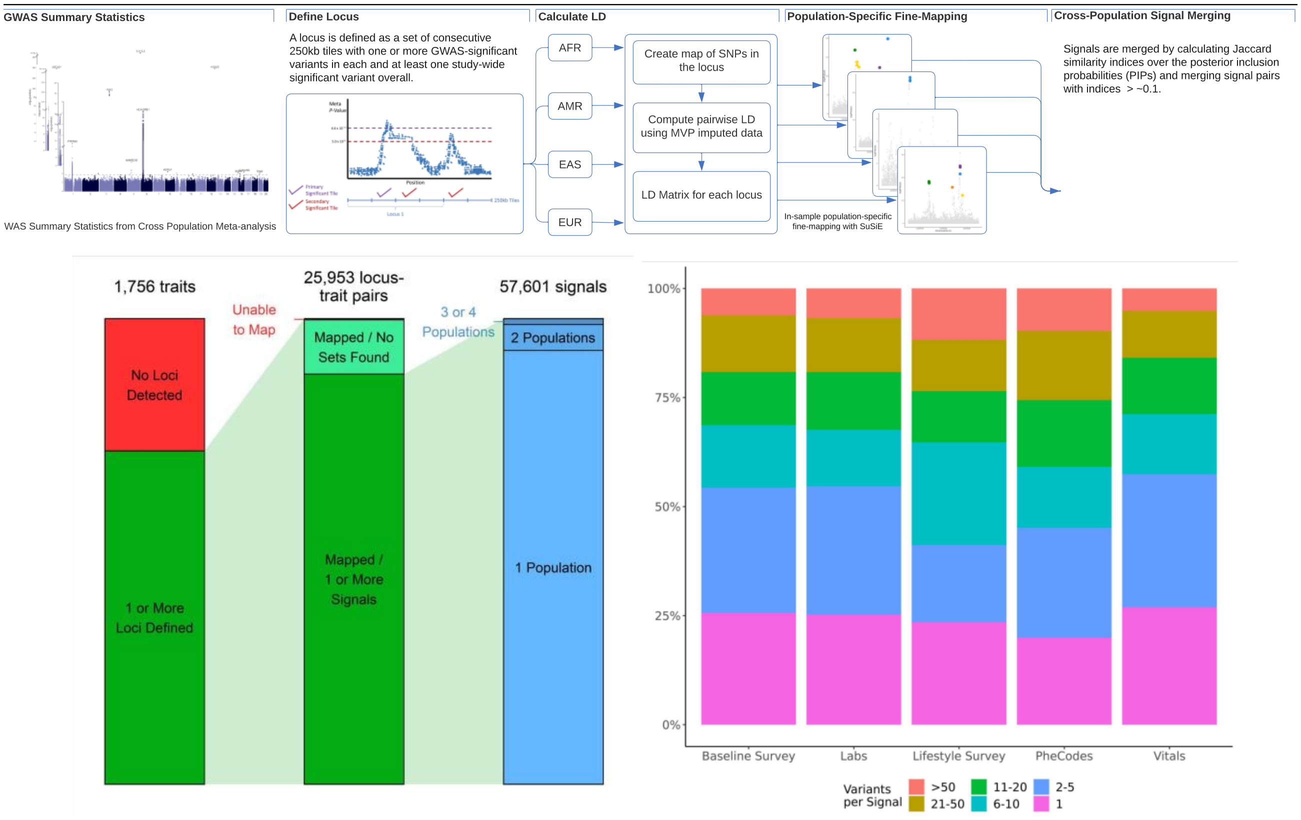

Figure S3. Overview of fine-mapping analysis and results. The top panel is a flow diagram illustrating the design of the fine-mapping analysis. The bottom left is a Sankey-style plot showing the number of traits with mappable loci, the number of successfully mapped locus-trait pairs, and the distribution of signals by the number of populations in which they were mapped. The bottom right bar chart reflects the precision with which signals were fine-mapped across the five main trait categories.

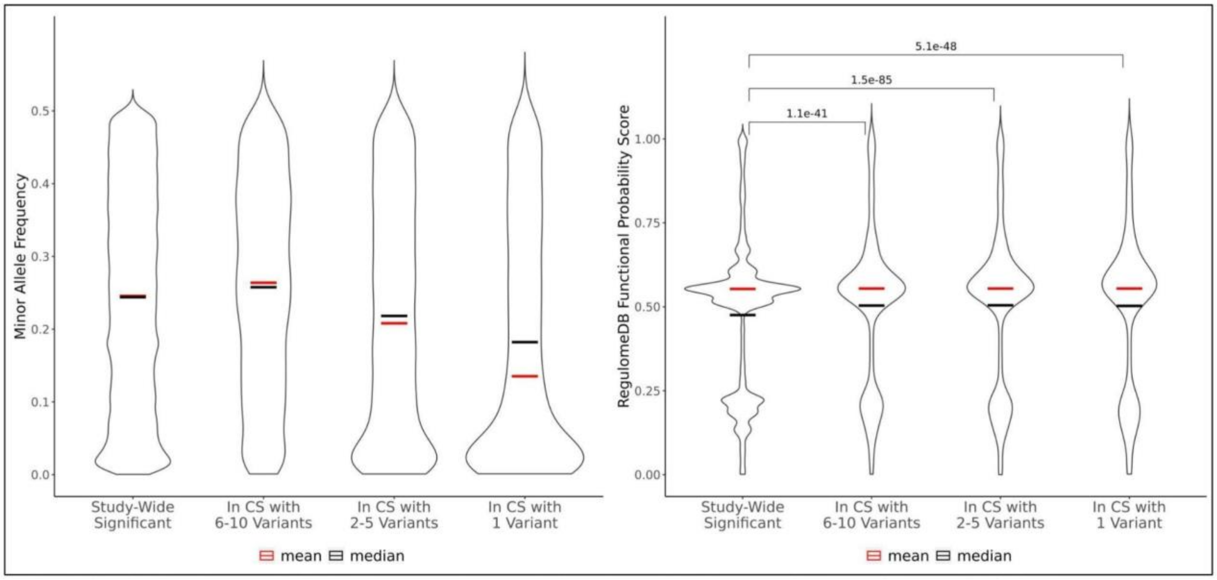

Figure S4. **Allele frequencies and RegulomeDB scores for fine-mapped variants.** Violin plots of allele frequencies (left) and RegulomeDB scores for fine-mapped variants in signals mapped at increasing levels of precision.

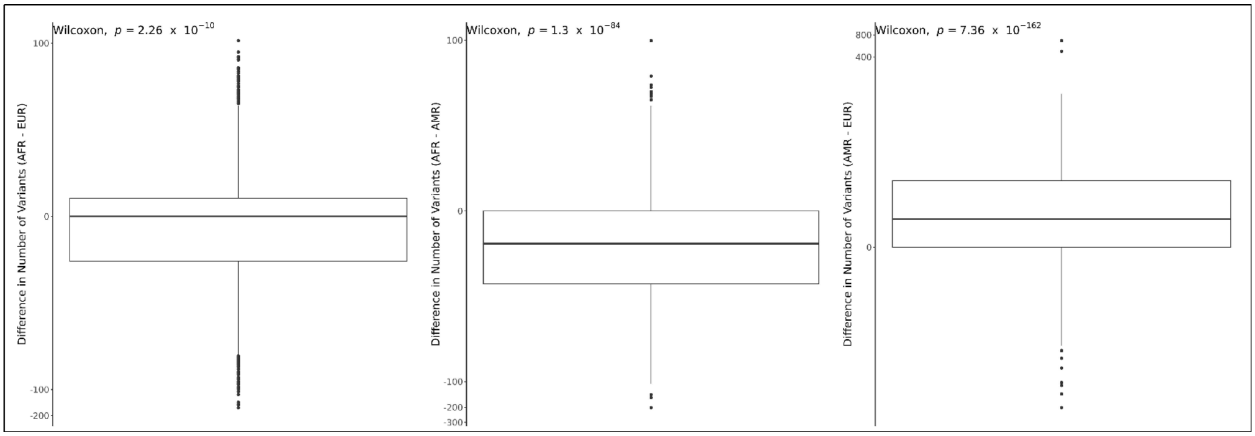

Figure S5. **Credible set size differences for signals mapped in multiple population groups.** Box plots illustrating the differences in credible set sizes for signals mapped in AFR and EUR (left), AFR and AMR (center), and AMR and EUR (right).

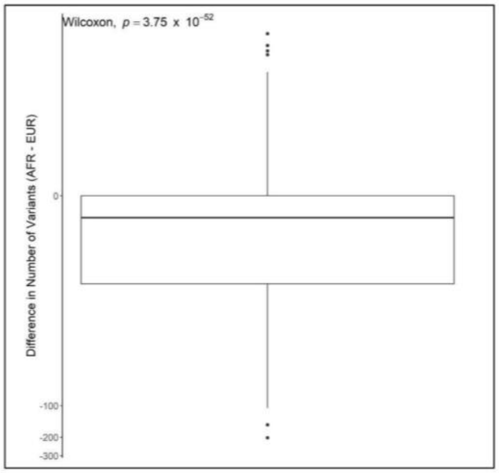

Figure S6. Differences in credible set sizes for signals mapped in size and composition-matched AFR and EUR groups. Box plot illustrating the differences in credible set sizes for signals mapped in AFR and EUR group after down-sampling the EUR group to match the size and age/sex composition of the AFR group.

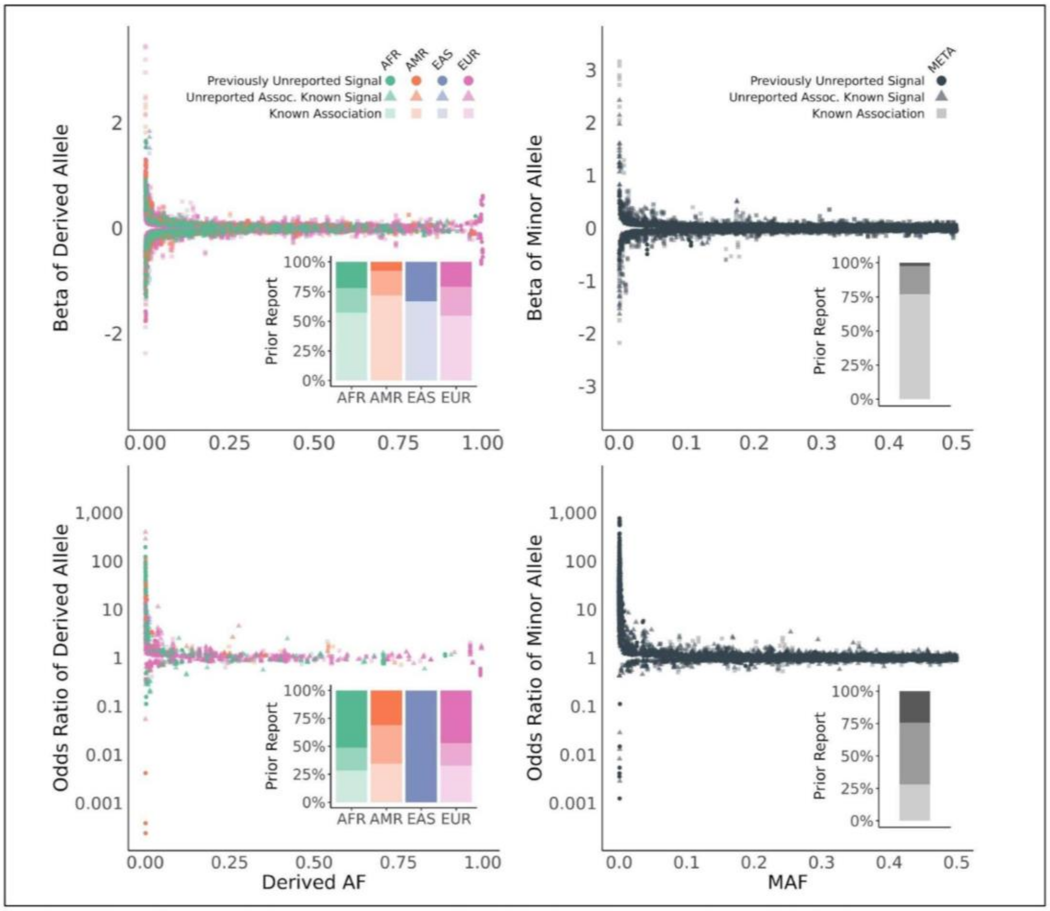

Figure S7. Effect sizes and allele frequencies for high-confidence and lead variants. Scatter plots of variant effect sizes (y-axis) vs allele frequencies (x-axis) for high-confidence fine-mapped variants (PIP > 0.95; left), and lead meta-analysis GWAS variants (right). Quantitative trait associations are plotted on top with respect to regression betas as effect sizes and binary trait associations are plotted at bottom with odds ratios. Variant associations are colored by population in which the association or mapping was made. Fine-mapped variant-trait associations may appear twice on the high-confidence plots if they were mapped in multiple populations. Point shapes and inset bar plots reflect whether the associations were previously reported in the GWAS Catalog and Open Targets Genetics. Fine-mapped variants are plotted with respect to derived allele frequencies and lead variants, with respect to minor allele frequencies.

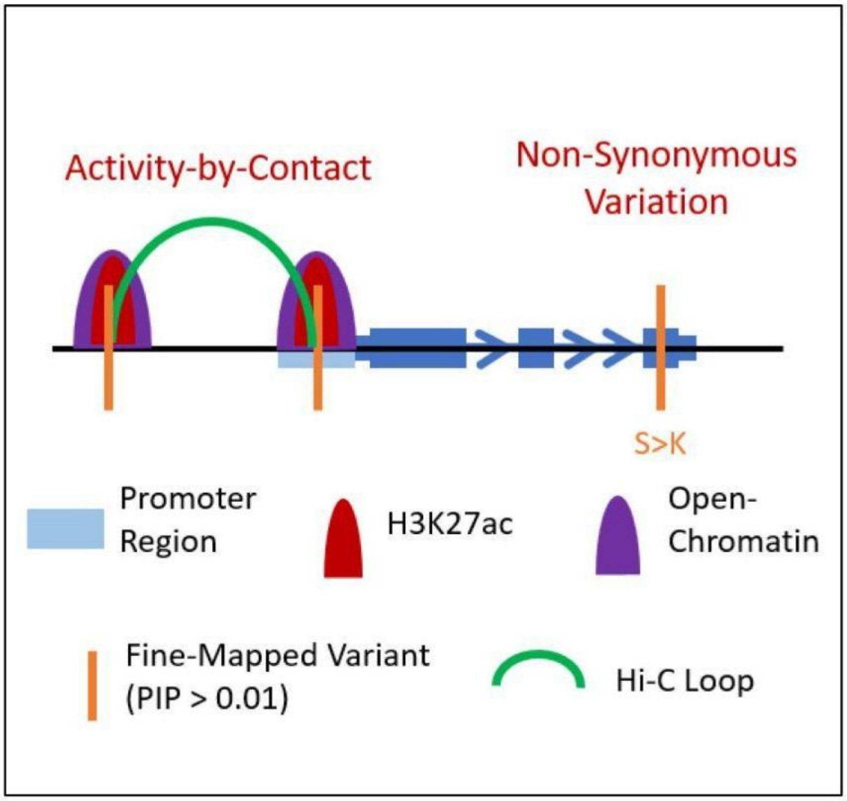

Figure S8. Overview of gene nomination scheme. Illustration of gene nomination scheme utilizing non-synonymous variation and the Activity-by-Contact model. Under the scheme, all three fine-mapped variants with PIP > 0.01 would be linked to the gene shown in blue. The left most variant overlaps a promoter-interacting ABC enhancer marked by H3K27ac and open-chromatin. The middle variant lies within an active promoter, and the right most variant is a non-synonymous missense variant in a coding region.

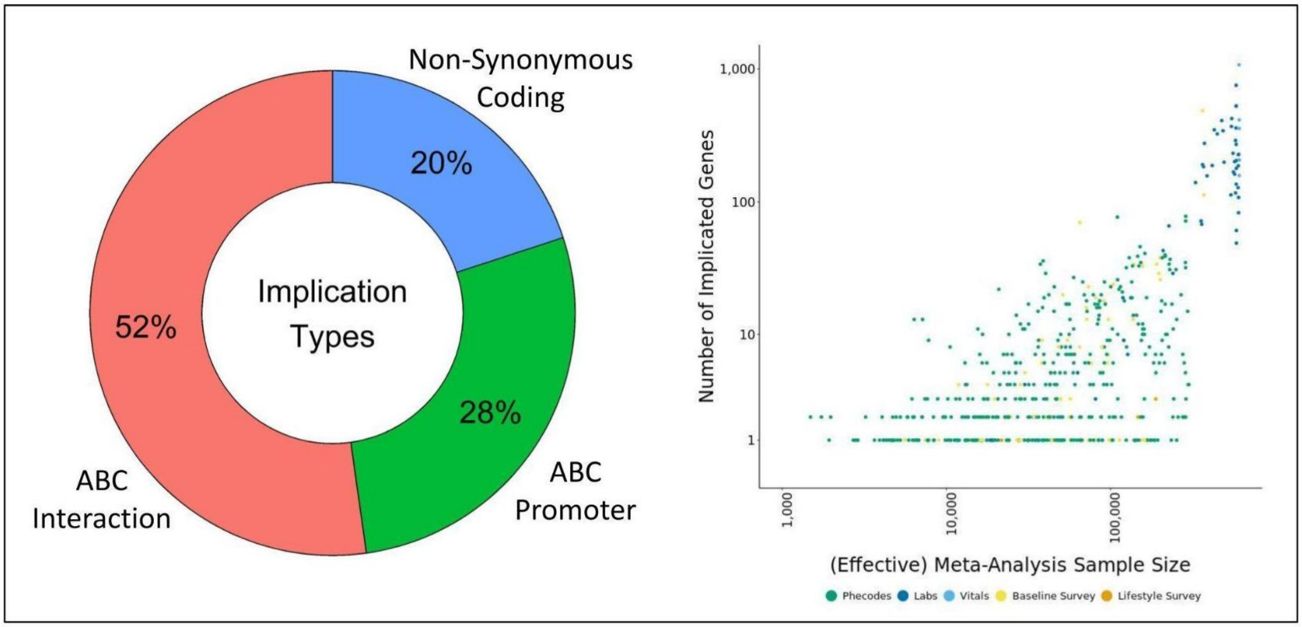

Figure S9. Distribution of gene nominations by method and trait. The donut plot on the left shows the distribution of gene implications across the three nomination categories. The scatter plot at right shows the number of implicated genes per trait vs. the effective meta-analysis sample size of each trait. Log scales are used for both x and y-axes, and the traits are colored by category.

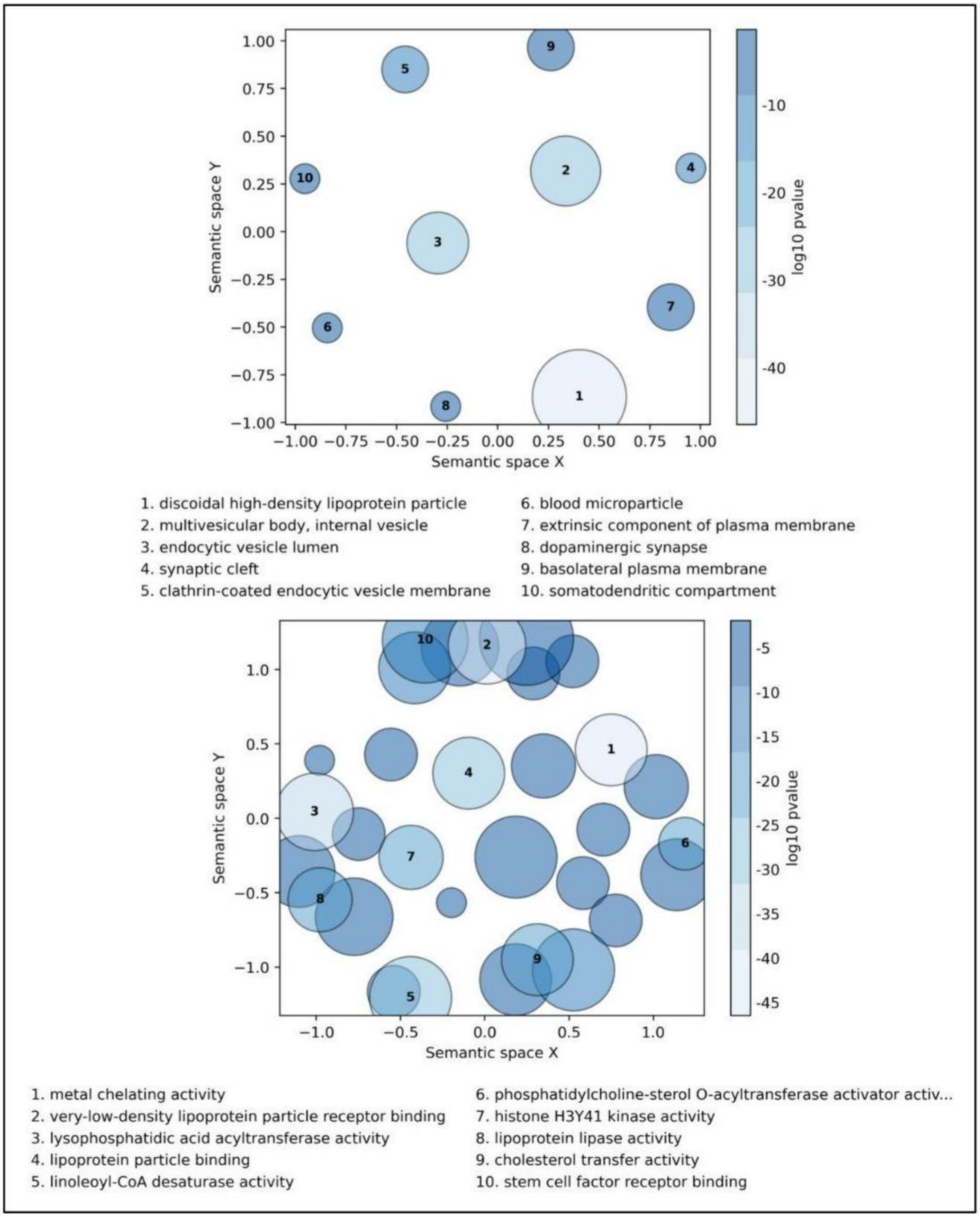

Figure S10. GO-Figure plots of cellular component and molecular function gene ontologies. GO-Figure plot showing clustering of cellular component (top) and molecular function (bottom) gene ontology (GO) terms significantly associated with the number of independent traits nominated per gene (adj. $P < 0.05$). Traits are clustered within each plot by weighted distance on the GO term graph, and clusters with more traits are plotted as larger circles. Coloring reflects the adjusted associated p-values of the representative GO term chosen for each cluster by the GO-Figure algorithm.

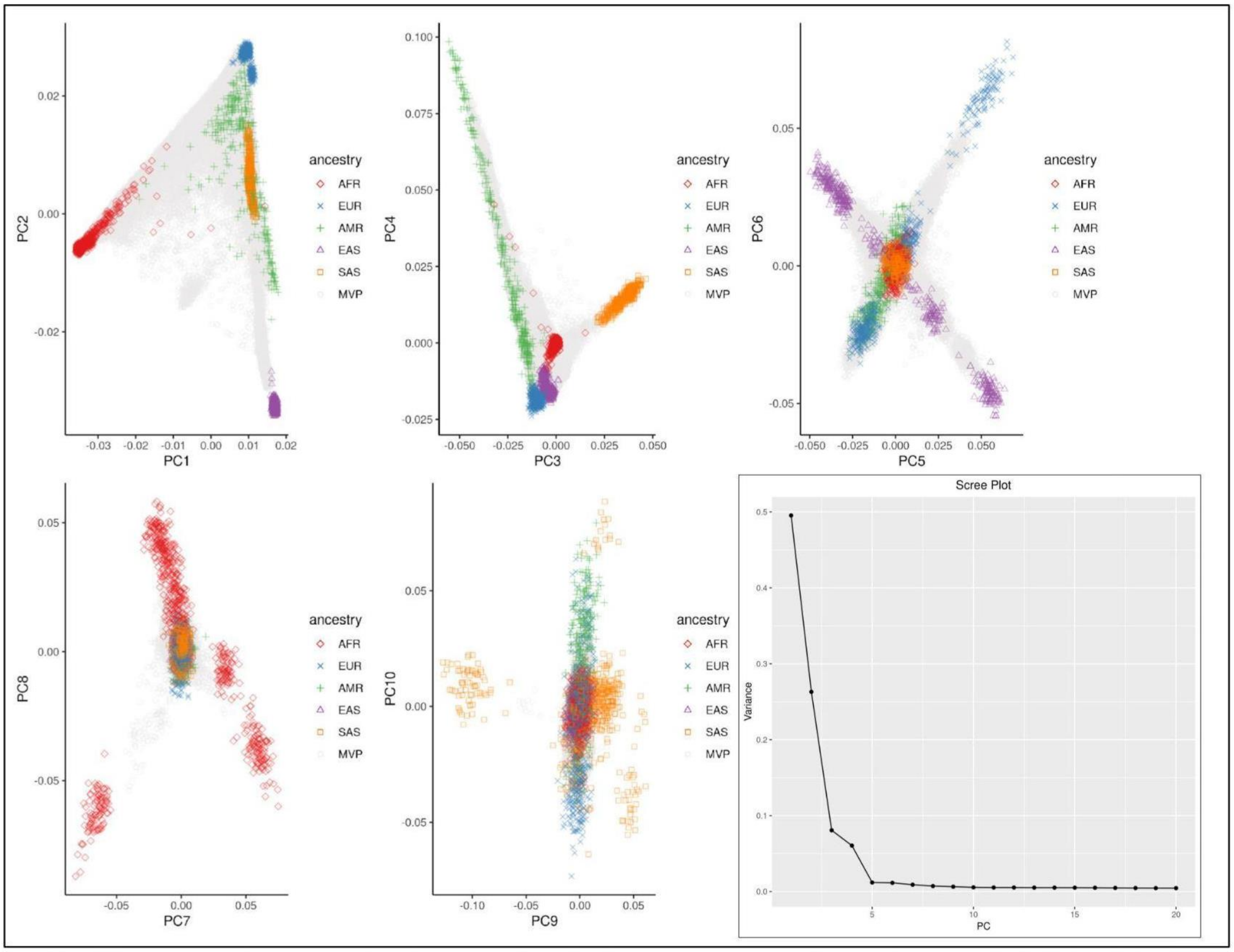

Figure S11. Determination of Genetic Ancestry via Principal Components Analysis. The first ten principal components demonstrate how the MVP participants, represented in gray, are projected onto the 1000 Genomes Project superpopulations (AFR=African, EUR=European, AMR=Admixed American, EAS=East Asian, SAS=South Asian, MVP=Million Veteran Program). Scatter plots show population separation by labeled principal components. The bottom right plot shows a scree plot that indicates the variation explained by each principal component.

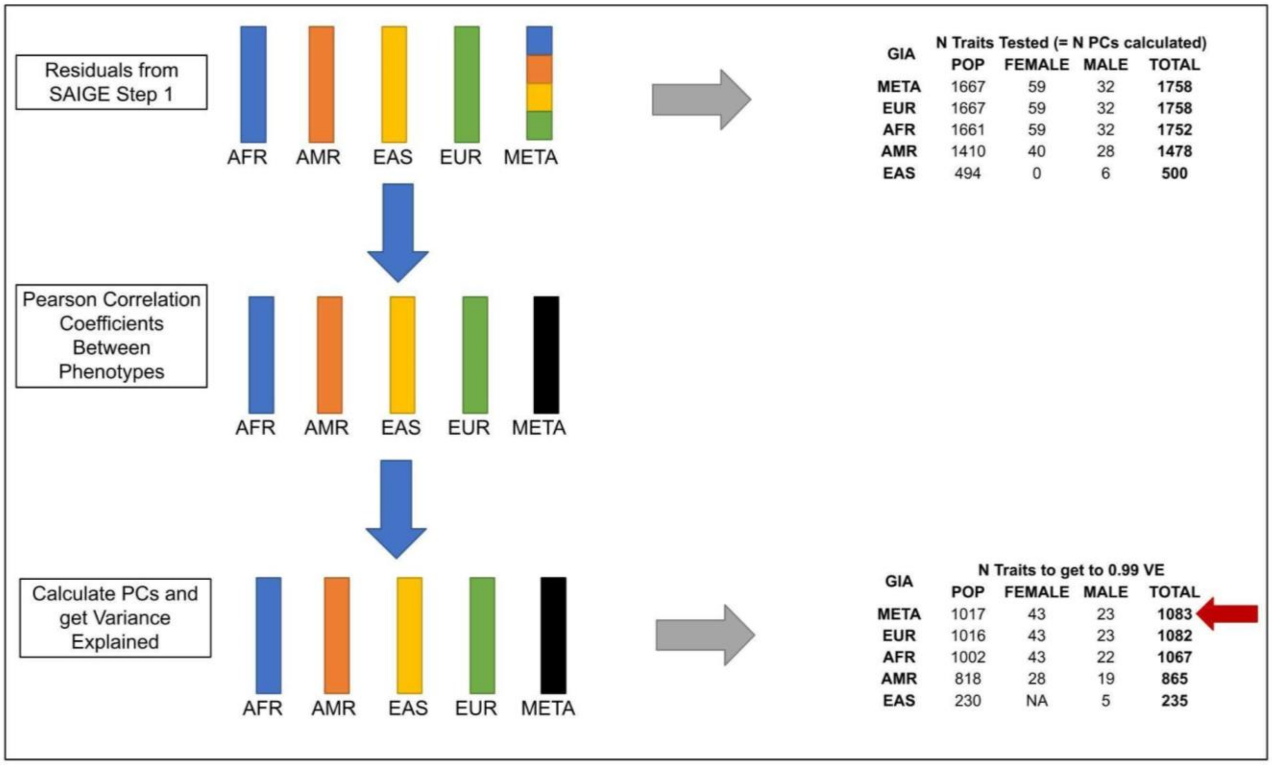

Figure S12. Flow diagram for determining the number of independent traits analyzed. AFR = African population, AMR = Admixed American population, EAS = East Asian population, EUR = European population, META = all people included in the meta-analysis, GIA = genetically informed ancestry, POP = trait measured in everyone (lung cancer), FEMALE = trait only present in females (ovarian cancer), MALE = traits present only in males (prostate cancer), PC = principal component, VE = variance explained. Red arrow indicates the number used for Bonferroni correction. “Female” and “Male” trait was determined by the clinical outcome description, not MVP participant-level biological sex. It was verified by comparing counts in biological male and female MVP participants with biological sex defined through the EHR or self-report, not genetics.

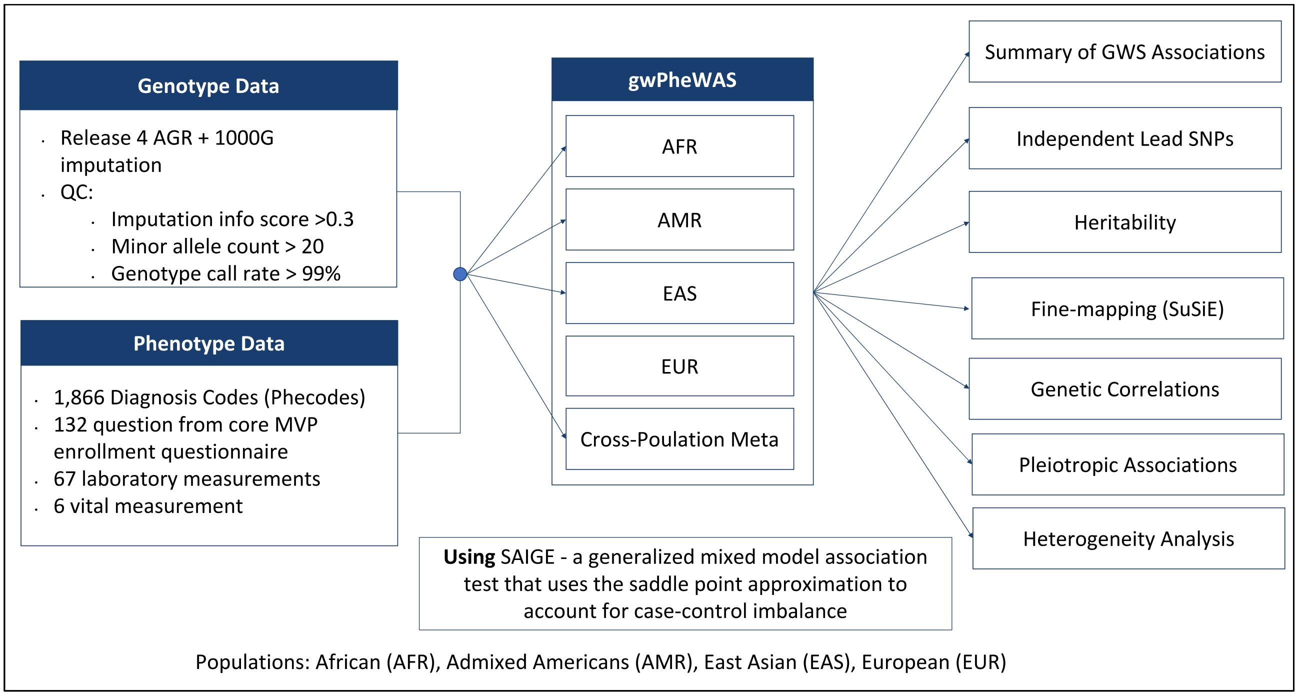

Figure S13. Study design overview. This figure depicts the genotype and phenotype data sources, the populations which underwent genome-wide association analysis, and the subsequent post-GWAS analyses undertaken to digest and summarize the results. GWS=genome-wide significant.

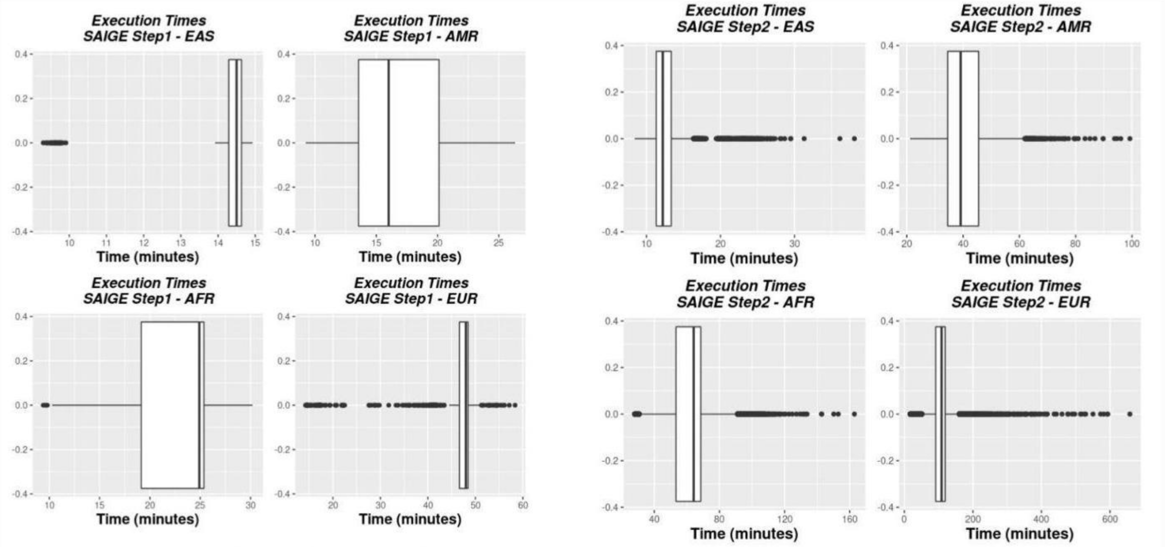

	EAS	AMR	AFR	EUR	Total
	<chr>	<chr>	<chr>	<chr>	<chr>
Total jobs	503	1480	1758	2070	5811
Mean/job (hours)	0.23	0.28	0.38	0.75	NA
Total GPU time (hours)	116.09	411.39	661.66	1548.03	2737.17
Total Node time (hours)	19.35	68.56	220.55	2064.04	2372.51

	EAS	AMR	AFR	EUR	Total
	<chr>	<chr>	<chr>	<chr>	<chr>
Total jobs	110157	324120	385002	453330	1272609
Mean/job (hours)	0.21	0.66	1.03	1.68	NA
Total CPU time (hours)	22723.68	214352.78	397428.04	763101.88	1397606.38
Total Node time (hours)	541.04	5103.64	9462.57	18169.09	33276.34

Figure S14. Execution times of optimized SAIGE on OLCF supercomputing infrastructure. Box plots of execution times for optimized SAIGE split by SAIGE step (left half vs. right half) and population. Tables at bottom presents summary-level statistics of distributions captured in the box plots.

Tables (provided in Excel format)

- **Table S1.** Demographics and Characteristics of Study Participants
- **Table S2.** Prevalence of Traits and Prevalence Differences across Four Populations
- **Table S3.** Population specific Genomic Loci Associations
- **Table S4.** LDSC Heritability Estimates of Traits by Population Groups
- **Table S5.** Cross-population Genetic Correlations of Traits with EUR as Reference Population Using Popcorn
- **Table S6.** Meta-analysis Associations Across 13,672 Genomic Risk Loci for 1,270 traits
- **Table S7.** Summary of Associations Across 40 Broad Trait Categories
- **Table S8.** Overview of Genetic Associations and Fine-Mapping Signals Across 1270 Traits
- **Table S9.** Summary of Genomic Loci Significant Exclusively in Non-European Population Groups and Lacking Significance (P<4.6e-11) in European Population Group
- **Table S10.** Fine-Mapping outcomes of 25,953 Defined Phenotype-locus Pairs
- **Table S11.** Signal-level Summary Information of 57,601 Signals Mapped Across 936 Phenotypes
- **Table S12.** Variants Fine-Mapped with High-Confidence in Non-EUR Groups that were Unmapped or Mapped with Low-Confidence in EUR Group
- **Table S13.** Heterogeneous Effect Sizes of Loci-Trait Associations Across All Fine-Mapped Loci, Compared Between A) African and European Groups (AFR vs EUR) and B) between Admixed American and European Groups (AMR vs EUR)
- **Table S14**. Heterogeneous Effect Sizes of Fine-Mapped Loci-Trait Associations Located in Coding Regions, Compared Between A) African and European Groups (AFR vs EUR) and B) Between Admixed American and European Groups (AMR vs EUR)
- **Table S15.** Gene Nominations for Variants Mapped with PIP > 0.01
- **Table S16.** Over-Represented KEGG Pathways for Each Trait
- **Table S17.** Independent Trait-Gene Associations (rg < 0.2)
- **Table S18.** GO Terms Significantly Associated with Number of Independent Traits per Gene
- **Table S19.** GO Terms Significantly Associated with Number of Independent Traits per Gene
- **Table S20.** Variant Effect Predictor (VEP) annotations

Additional Data Files

- **Data S1.** Variant-level details of 57,601 signals mapped across 936 phenotypes

References and Notes

1. M. C. Mills, C. Rahal, The GWAS Diversity Monitor tracks diversity by disease in real time. *Nat. Genet.* **52**, 242–243 (2020). doi:10.1038/s41588-020-0580-y Medline

2. A. R. Martin, M. Kanai, Y. Kamatani, Y. Okada, B. M. Neale, M. J. Daly, Clinical use of current polygenic risk scores may exacerbate health disparities. *Nat. Genet.* **51**, 584–591 (2019). doi:10.1038/s41588-019-0379-x Medline

3. Z. Chen, J. Chen, R. Collins, Y. Guo, R. Peto, F. Wu, L. Li; China Kadoorie Biobank (CKB) collaborative group, China Kadoorie Biobank of 0.5 million people: Survey methods, baseline characteristics and long-term follow-up. *Int. J. Epidemiol.* **40**, 1652–1666 (2011). doi:10.1093/ije/dyr120 Medline

4. A. Nagai, M. Hirata, Y. Kamatani, K. Muto, K. Matsuda, Y. Kiyohara, T. Ninomiya, A. Tamakoshi, Z. Yamagata, T. Mushiroda, Y. Murakami, K. Yuji, Y. Furukawa, H. Zembutsu, T. Tanaka, Y. Ohnishi, Y. Nakamura, M. Kubo, M. Shiono, K. Misumi, R. Kaieda, H. Harada, S. Minami, M. Emi, N. Emoto, H. Daida, K. Miyauchi, A. Murakami, S. Asai, M. Moriyama, Y. Takahashi, T. Fujioka, W. Obara, S. Mori, H. Ito, S. Nagayama, Y. Miki, A. Masumoto, A. Yamada, Y. Nishizawa, K. Kodama, H. Kutsumi, Y. Sugimoto, Y. Koretsune, H. Kusuoka, H. Yanai, BioBank Japan Cooperative Hospital Group, Overview of the BioBank Japan Project: Study design and profile. *J. Epidemiol.* **27** (3S), S2–S8 (2017). doi:10.1016/j.je.2016.12.005 Medline

5. G. Kolata, "V.A. Recruits Millionth Veteran for Its Genetic Research Database" in *The New York Times* (2023). https://www.nytimes.com/2023/11/15/health/million-veterans-database-va.html.

6. All of Us Research Program Investigators, J. C. Denny, J. L. Rutter, D. B. Goldstein, A. Philippakis, J. W. Smoller, G. Jenkins, E. Dishman, The "All of Us" Research Program. *N. Engl. J. Med.* **381**, 668–676 (2019). doi:10.1056/NEJMsr1809937 Medline

7. J. M. Gaziano, J. Concato, M. Brophy, L. Fiore, S. Pyarajan, J. Breeling, S. Whitbourne, J. Deen, C. Shannon, D. Humphries, P. Guarino, M. Aslan, D. Anderson, R. LaFleur, T. Hammond, K. Schaa, J. Moser, G. Huang, S. Muralidhar, R. Przygodzki, T. J. O'Leary, Million Veteran Program: A mega-biobank to study genetic influences on health and disease. *J. Clin. Epidemiol.* **70**, 214–223 (2016). doi:10.1016/j.jclinepi.2015.09.016 Medline

8. A. Auton, G. R. Abecasis, D. M. Altshuler, R. M. Durbin, G. R. Abecasis, D. R. Bentley, A. Chakravarti, A. G. Clark, P. Donnelly, E. E. Eichler, P. Flicek, S. B. Gabriel, R. A. Gibbs, E. D. Green, M. E. Hurles, B. M. Knoppers, J. O. Korbel, E. S. Lander, C. Lee, H. Lehrach, E. R. Mardis, G. T. Marth, G. A. McVean, D. A. Nickerson, J. P. Schmidt, S. T. Sherry, J. Wang, R. K. Wilson, R. A. Gibbs, E. Boerwinkle, H. Doddapaneni, Y. Han, V. Korchina, C. Kovar, S. Lee, D. Muzny, J. G. Reid, Y. Zhu, J. Wang, Y. Chang, Q. Feng, X. Fang, X. Guo, M. Jian, H. Jiang, X. Jin, T. Lan, G. Li, J. Li, Y. Li, S. Liu, X. Liu, Y. Lu, X. Ma, M. Tang, B. Wang, G. Wang, H. Wu, R. Wu, X. Xu, Y. Yin, D. Zhang, W. Zhang, J. Zhao, M. Zhao, X. Zheng, E. S. Lander, D. M. Altshuler, S. B. Gabriel, N. Gupta, N. Gharani, L. H. Toji, N. P. Gerry, A. M. Resch, P. Flicek, J. Barker, L. Clarke, L. Gil, S. E. Hunt, G. Kelman, E. Kulesha, R. Leinonen, W. M. McLaren, R. Radhakrishnan, A. Roa, D. Smirnov, R. E. Smith, I. Streeter, A. Thormann, I. Toneva, B.

Vaughan, X. Zheng-Bradley, D. R. Bentley, R. Grocock, S. Humphray, T. James, Z. Kingsbury, H. Lehrach, R. Sudbrak, M. W. Albrecht, V. S. Amstislavskiy, T. A. Borodina, M. Lienhard, F. Mertes, M. Sultan, B. Timmermann, M.-L. Yaspo, E. R. Mardis, R. K. Wilson, L. Fulton, R. Fulton, S. T. Sherry, V. Ananiev, Z. Belaia, D. Beloslyudtsev, N. Bouk, C. Chen, D. Church, R. Cohen, C. Cook, J. Garner, T. Hefferon, M. Kimelman, C. Liu, J. Lopez, P. Meric, C. O'Sullivan, Y. Ostapchuk, L. Phan, S. Ponomarov, V. Schneider, E. Shekhtman, K. Sirotkin, D. Slotta, H. Zhang, G. A. McVean, R. M. Durbin, S. Balasubramaniam, J. Burton, P. Danecek, T. M. Keane, A. Kolb-Kokocinski, S. McCarthy, J. Stalker, M. Quail, J. P. Schmidt, C. J. Davies, J. Gollub, T. Webster, B. Wong, Y. Zhan, A. Auton, C. L. Campbell, Y. Kong, A. Marcketta, R. A. Gibbs, F. Yu, L. Antunes, M. Bainbridge, D. Muzny, A. Sabo, Z. Huang, J. Wang, L. J. M. Coin, L. Fang, X. Guo, X. Jin, G. Li, Q. Li, Y. Li, Z. Li, H. Lin, B. Liu, R. Luo, H. Shao, Y. Xie, C. Ye, C. Yu, F. Zhang, H. Zheng, H. Zhu, C. Alkan, E. Dal, F. Kahveci, G. T. Marth, E. P. Garrison, D. Kural, W.-P. Lee, W. Fung Leong, M. Stromberg, A. N. Ward, J. Wu, M. Zhang, M. J. Daly, M. A. DePristo, R. E. Handsaker, D. M. Altshuler, E. Banks, G. Bhatia, G. Angel, S. B. Gabriel, G. Genovese, N. Gupta, H. Li, S. Kashin, E. S. Lander, S. A. McCarroll, J. C. Nemesh, R. E. Poplin, S. C. Yoon, J. Lihm, V. Makarov, A. G. Clark, S. Gottipati, A. Keinan, J. L. Rodriguez-Flores, J. O. Korbel, T. Rausch, M. H. Fritz, A. M. Stütz, P. Flicek, K. Beal, L. Clarke, A. Datta, J. Herrero, W. M. McLaren, G. R. S. Ritchie, R. E. Smith, D. Zerbino, X. Zheng-Bradley, P. C. Sabeti, I. Shlyakhter, S. F. Schaffner, J. Vitti, D. N. Cooper, E. V. Ball, P. D. Stenson, D. R. Bentley, B. Barnes, M. Bauer, R. Keira Cheetham, A. Cox, M. Eberle, S. Humphray, S. Kahn, L. Murray, J. Peden, R. Shaw, E. E. Kenny, M. A. Batzer, M. K. Konkel, J. A. Walker, D. G. MacArthur, M. Lek, R. Sudbrak, V. S. Amstislavskiy, R. Herwig, E. R. Mardis, L. Ding, D. C. Koboldt, D. Larson, K. Ye, S. Gravel, The 1000 Genomes Project Consortium, A global reference for human genetic variation. *Nature* **526**, 68–74 (2015). Medline

9. Detailed materials and methods are available as supplementary materials.

10. W. Zhou, J. B. Nielsen, L. G. Fritsche, R. Dey, M. E. Gabrielsen, B. N. Wolford, J. LeFaive, P. VandeHaar, S. A. Gagliano, A. Gifford, L. A. Bastarache, W.-Q. Wei, J. C. Denny, M. Lin, K. Hveem, H. M. Kang, G. R. Abecasis, C. J. Willer, S. Lee, Efficiently controlling for case-control imbalance and sample relatedness in large-scale genetic association studies. *Nat. Genet.* **50**, 1335–1341 (2018). doi:10.1038/s41588-018-0184-y Medline

11. E. Sollis, A. Mosaku, A. Abid, A. Buniello, M. Cerezo, L. Gil, T. Groza, O. Güneş, P. Hall, J. Hayhurst, A. Ibrahim, Y. Ji, S. John, E. Lewis, J. A. L. MacArthur, A. McMahon, D. Osumi-Sutherland, K. Panoutsopoulou, Z. Pendlington, S. Ramachandran, R. Stefancsik, J. Stewart, P. Whetzel, R. Wilson, L. Hindorff, F. Cunningham, S. A. Lambert, M. Inouye, H. Parkinson, L. W. Harris, The NHGRI-EBI GWAS Catalog: Knowledgebase and deposition resource. *Nucleic Acids Res.* **51**, D977–D985 (2023). doi:10.1093/nar/gkac1010 Medline

12. M. Ghoussaini, E. Mountjoy, M. Carmona, G. Peat, E. M. Schmidt, A. Hercules, L. Fumis, A. Miranda, D. Carvalho-Silva, A. Buniello, T. Burdett, J. Hayhurst, J. Baker, J. Ferrer, A. Gonzalez-Uriarte, S. Jupp, M. A. Karim, G. Koscielny, S. Machlitt-Northen, C. Malangone, Z. M. Pendlington, P. Roncaglia, D. Suveges, D. Wright, O. Vrousgou, E. Papa, H. Parkinson, J. A. L. MacArthur, J. A. Todd, J. C. Barrett, J. Schwartzentruber, D.

G. Hulcoop, D. Ochoa, E. M. McDonagh, I. Dunham, Open Targets Genetics: Systematic identification of trait-associated genes using large-scale genetics and functional genomics. *Nucleic Acids Res.* **49**, D1311–D1320 (2021). doi:10.1093/nar/gkaa840 Medline

13. B. C. Brown, C. J. Ye, A. L. Price, N. Zaitlen, Asian Genetic Epidemiology Network Type 2 Diabetes Consortium, Transethnic Genetic-Correlation Estimates from Summary Statistics. *Am. J. Hum. Genet.* **99**, 76–88 (2016). doi:10.1016/j.ajhg.2016.05.001 Medline

14. B. Bulik-Sullivan, H. K. Finucane, V. Anttila, A. Gusev, F. R. Day, P.-R. Loh, L. Duncan, J. R. B. Perry, N. Patterson, E. B. Robinson, M. J. Daly, A. L. Price, B. M. Neale, ReproGen Consortium; Psychiatric Genomics Consortium; Genetic Consortium for Anorexia Nervosa of the Wellcome Trust Case Control Consortium 3, An atlas of genetic correlations across human diseases and traits. *Nat. Genet.* **47**, 1236–1241 (2015). doi:10.1038/ng.3406 Medline

15. D. Reich, M. A. Nalls, W. H. L. Kao, E. L. Akylbekova, A. Tandon, N. Patterson, J. Mullikin, W.-C. Hsueh, C.-Y. Cheng, J. Coresh, E. Boerwinkle, M. Li, A. Waliszewska, J. Neubauer, R. Li, T. S. Leak, L. Ekunwe, J. C. Files, C. L. Hardy, J. M. Zmuda, H. A. Taylor, E. Ziv, T. B. Harris, J. G. Wilson, Reduced neutrophil count in people of African descent is due to a regulatory variant in the Duffy antigen receptor for chemokines gene. *PLOS Genet.* **5**, e1000360 (2009). doi:10.1371/journal.pgen.1000360 Medline

16. A. R. Bentley, J. Divers, D. Shriner, A. P. Doumatey, O. M. Gutiérrez, A. A. Adeyemo, B. I. Freedman, C. N. Rotimi, APOL1 G1 genotype modifies the association between HDLC and kidney function in African Americans. *BMC Genomics* **16**, 421 (2015). doi:10.1186/s12864-015-1645-7 Medline

17. T. P. Joshi, D. Garcia, F. Gedeon, D. Hinson, E. Strouphauer, F. Okundia, J. Tschen, Epidemiology of alopecia areata in the Hispanic/Latinx community: A cross-sectional analysis of the All of Us database. *J. Am. Acad. Dermatol.* **89**, e61–e62 (2023). doi:10.1016/j.jaad.2023.02.054 Medline

18. G. Wang, A. Sarkar, P. Carbonetto, M. Stephens, A simple new approach to variable selection in regression, with application to genetic fine mapping. *J. R. Stat. Soc. Series B Stat. Methodol.* **82**, 1273–1300 (2020). doi:10.1111/rssb.12388 Medline

19. Y. Zou, P. Carbonetto, G. Wang, M. Stephens, Fine-mapping from summary data with the “Sum of Single Effects” model. *PLOS Genet.* **18**, e1010299 (2022). doi:10.1371/journal.pgen.1010299 Medline

20. M. Kanai, J. C. Ulirsch, J. Karjalainen, M. Kurki, K. J. Karczewski, E. Fauman, Q. S. Wang, H. Jacobs, F. Aguet, K. G. Ardlie, N. Kerimov, K. Alasoo, C. Benner, K. Ishigaki, S. Sakaue, S. Reilly, The BioBank Japan Project, FinnGen, Y. Kamatani, K. Matsuda, A. Palotie, B. M. Neale, R. Tewhey, P. C. Sabeti, Y. Okada, M. J. Daly, H. K. Finucane, Insights from complex trait fine-mapping across diverse populations. medRxiv 2021.09.03.21262975 [Preprint] (2021); doi:10.1101/2021.09.03.21262975.

21. A. Verma, Diversity and Scale: Genetic Architecture of 2,068 Traits in the VA Million Veteran Program Data S1, Dryad (2023); https://doi.org/10.5061/dryad.zgmsbcck4.

22. J.-H. Park, M. H. Gail, C. R. Weinberg, R. J. Carroll, C. C. Chung, Z. Wang, S. J. Chanock, J. F. Fraumeni Jr., N. Chatterjee, Distribution of allele frequencies and effect sizes and their interrelationships for common genetic susceptibility variants. *Proc. Natl. Acad. Sci. U.S.A.* **108**, 18026–18031 (2011). doi:10.1073/pnas.1114759108 Medline

23. A. P. Schoech, D. M. Jordan, P.-R. Loh, S. Gazal, L. J. O'Connor, D. J. Balick, P. F. Palamara, H. K. Finucane, S. R. Sunyaev, A. L. Price, Quantification of frequency-dependent genetic architectures in 25 UK Biobank traits reveals action of negative selection. *Nat. Commun.* **10**, 790 (2019). doi:10.1038/s41467-019-08424-6 Medline

24. G. Wang, J. R. Speakman, Analysis of Positive Selection at Single Nucleotide Polymorphisms Associated with Body Mass Index Does Not Support the "Thrifty Gene" Hypothesis. *Cell Metab.* **24**, 531–541 (2016). doi:10.1016/j.cmet.2016.08.014 Medline

25. S. Wilde, A. Timpson, K. Kirsanow, E. Kaiser, M. Kayser, M. Unterländer, N. Hollfelder, I. D. Potekhina, W. Schier, M. G. Thomas, J. Burger, Direct evidence for positive selection of skin, hair, and eye pigmentation in Europeans during the last 5,000 y. *Proc. Natl. Acad. Sci. U.S.A.* **111**, 4832–4837 (2014). doi:10.1073/pnas.1316513111 Medline

26. R. L. Lamason, M.-A. P. K. Mohideen, J. R. Mest, A. C. Wong, H. L. Norton, M. C. Aros, M. J. Jurynec, X. Mao, V. R. Humphreville, J. E. Humbert, S. Sinha, J. L. Moore, P. Jagadeeswaran, W. Zhao, G. Ning, I. Makalowska, P. M. McKeigue, D. O'donnell, R. Kittles, E. J. Parra, N. J. Mangini, D. J. Grunwald, M. D. Shriver, V. A. Canfield, K. C. Cheng, SLC24A5, a putative cation exchanger, affects pigmentation in zebrafish and humans. *Science* **310**, 1782–1786 (2005). doi:10.1126/science.1116238 Medline

27. F. Rajabli, G. W. Beecham, H. C. Hendrie, O. Baiyewu, A. Ogunniyi, S. Gao, N. A. Kushch, M. Lipkin-Vasquez, K. L. Hamilton-Nelson, J. I. Young, D. M. Dykxhoorn, K. Nuytemans, B. W. Kunkle, L. Wang, F. Jin, X. Liu, B. E. Feliciano-Astacio, G. D. Schellenberg, C. L. Dalgard, A. J. Griswold, G. S. Byrd, C. Reitz, M. L. Cuccaro, J. L. Haines, M. A. Pericak-Vance, J. M. Vance, Alzheimer's Disease Sequencing Project, Alzheimer's Disease Genetic Consortium, A locus at 19q13.31 significantly reduces the ApoE ε4 risk for Alzheimer's Disease in African Ancestry. *PLOS Genet.* **18**, e1009977 (2022). doi:10.1371/journal.pgen.1009977 Medline

28. R. Wrigley, A. J. Phipps-Green, R. K. Topless, T. J. Major, M. Cadzow, P. Riches, A.-K. Tausche, M. Janssen, L. A. B. Joosten, T. L. Jansen, A. So, J. Harré Hindmarsh, L. K. Stamp, N. Dalbeth, T. R. Merriman, Pleiotropic effect of the ABCG2 gene in gout: Involvement in serum urate levels and progression from hyperuricemia to gout. *Arthritis Res. Ther.* **22**, 45 (2020). doi:10.1186/s13075-020-2136-z Medline

29. M. O. Pilon, G. Leclair, E. Oussaïd, I. St-Jean, M. Jutras, M. J. Gaulin, I. Mongrain, D. Busseuil, J. L. Rouleau, J. C. Tardif, M. P. Dubé, S. de Denus, An association study of *ABCG2* rs2231142 on the concentrations of allopurinol and its metabolites. *Clin. Transl. Sci.* **15**, 2024–2034 (2022). doi:10.1111/cts.13318 Medline

30. K.-H. Yu, P.-Y. Chang, S.-C. Chang, Y.-H. Wu-Chou, L.-A. Wu, D.-P. Chen, F.-S. Lo, J.-J. Lu, A comprehensive analysis of the association of common variants of ABCG2 with gout. *Sci. Rep.* **7**, 9988 (2017). doi:10.1038/s41598-017-10196-2 Medline

31. L. M. Polfus, B. F. Darst, H. Highland, X. Sheng, M. C. Y. Ng, J. E. Below, L. Petty, S. Bien, X. Sim, W. Wang, P. Fontanillas, Y. Patel, M. Preuss, C. Schurmann, Z. Du, Y. Lu,

S. K. Rhie, J. M. Mercader, T. Tusie-Luna, C. González-Villalpando, L. Orozco, C. N. Spracklen, B. E. Cade, R. A. Jensen, M. Sun, Y. Y. Joo, P. An, L. R. Yanek, L. F. Bielak, S. Tajuddin, A. Nicolas, G. Chen, L. Raffield, X. Guo, W.-M. Chen, G. N. Nadkarni, M. Graff, R. Tao, J. S. Pankow, M. Daviglus, Q. Qi, E. A. Boerwinkle, S. Liu, L. S. Phillips, U. Peters, C. Carlson, L. R. Wikens, L. L. Marchand, K. E. North, S. Buyske, C. Kooperberg, R. J. F. Loos, D. O. Stram, C. A. Haiman, 23andMe Research Team; DIAMANTE Hispanic/Latino Consortium; MEta-analysis of type 2 DIabetes in African Americans Consortium, Genetic discovery and risk characterization in type 2 diabetes across diverse populations. *HGG Adv.* **2**, 100029 (2021). doi:10.1016/j.xhgg.2021.100029 Medline

32. J. Chen, M. Sun, A. Adeyemo, F. Pirie, T. Carstensen, C. Pomilla, A. P. Doumatey, G. Chen, E. H. Young, M. Sandhu, A. P. Morris, I. Barroso, M. I. McCarthy, A. Mahajan, E. Wheeler, C. N. Rotimi, A. A. Motala, Genome-wide association study of type 2 diabetes in Africa. *Diabetologia* **62**, 1204–1211 (2019). doi:10.1007/s00125-019-4880-7 Medline

33. C. P. Fulco, J. Nasser, T. R. Jones, G. Munson, D. T. Bergman, V. Subramanian, S. R. Grossman, R. Anyoha, B. R. Doughty, T. A. Patwardhan, T. H. Nguyen, M. Kane, E. M. Perez, N. C. Durand, C. A. Lareau, E. K. Stamenova, E. L. Aiden, E. S. Lander, J. M. Engreitz, Activity-by-contact model of enhancer-promoter regulation from thousands of CRISPR perturbations. *Nat. Genet.* **51**, 1664–1669 (2019). doi:10.1038/s41588-019-0538-0 Medline

34. Y. D. Bhutia, E. Babu, S. Ramachandran, S. Yang, M. Thangaraju, V. Ganapathy, SLC transporters as a novel class of tumour suppressors: Identity, function and molecular mechanisms. *Biochem. J.* **473**, 1113–1124 (2016). doi:10.1042/BJ20150751 Medline

35. T. W. Kim, D. H. Pyo, E. Ko, N. H. Yun, S. J. Song, S. M. Choi, H. K. Hong, S.-H. Kim, Y.-L. Choi, J. Lee, W. Y. Lee, Y. B. Cho, Expression of SLC22A18 regulates oxaliplatin resistance by modulating the ERK pathway in colorectal cancer. *Am. J. Cancer Res.* **12**, 1393–1408 (2022). Medline

36. A. Kousathanas *et al.*, Whole-genome sequencing reveals host factors underlying critical COVID-19. *Nature* **607**, 97–103 (2022). doi:10.1038/s41586-022-04576-6 Medline

37. B. F. Darst, P. Wan, X. Sheng, J. T. Bensen, S. A. Ingles, B. A. Rybicki, B. Nemesure, E. M. John, J. H. Fowke, V. L. Stevens, S. I. Berndt, C. D. Huff, S. S. Strom, J. Y. Park, W. Zheng, E. A. Ostrander, P. C. Walsh, S. Srivastava, J. Carpten, T. A. Sellers, K. Yamoah, A. B. Murphy, M. Sanderson, D. C. Crawford, S. M. Gapstur, W. S. Bush, M. C. Aldrich, O. Cussenot, M. Yeager, G. Petrovics, J. Cullen, C. Neslund-Dudas, R. A. Kittles, J. Xu, M. C. Stern, Z. Kote-Jarai, K. Govindasami, A. P. Chokkalingam, L. Multigner, M.-E. Parent, F. Menegaux, G. Cancel-Tassin, A. S. Kibel, E. A. Klein, P. J. Goodman, B. F. Drake, J. J. Hu, P. E. Clark, P. Blanchet, G. Casey, A. J. M. Hennis, A. Lubwama, I. M. Thompson Jr., R. Leach, S. M. Gundell, L. Pooler, L. Xia, J. L. Mohler, E. T. H. Fontham, G. J. Smith, J. A. Taylor, R. A. Eeles, L. Brureau, S. J. Chanock, S. Watya, J. L. Stanford, D. Mandal, W. B. Isaacs, K. Cooney, W. J. Blot, D. V. Conti, C. A. Haiman, A Germline Variant at 8q24 Contributes to Familial Clustering of Prostate Cancer in Men of African Ancestry. *Eur. Urol.* **78**, 316–320 (2020). doi:10.1016/j.eururo.2020.04.060 Medline

38. O. A. Panagiotou, R. C. Travis, D. Campa, S. I. Berndt, S. Lindstrom, P. Kraft, F. R. Schumacher, A. Siddiq, S. I. Papatheodorou, J. L. Stanford, D. Albanes, J. Virtamo, S. J. Weinstein, W. R. Diver, S. M. Gapstur, V. L. Stevens, H. Boeing, H. B. Bueno-de-Mesquita, A. Barricarte Gurrea, R. Kaaks, K.-T. Khaw, V. Krogh, K. Overvad, E. Riboli, D. Trichopoulos, E. Giovannucci, M. Stampfer, C. Haiman, B. Henderson, L. Le Marchand, J. M. Gaziano, D. J. Hunter, S. Koutros, M. Yeager, R. N. Hoover, S. J. Chanock, S. Wacholder, T. J. Key, K. K. Tsilidis; PRACTICAL Consortium, A genome-wide pleiotropy scan for prostate cancer risk. *Eur. Urol.* **67**, 649–657 (2015). doi:10.1016/j.eururo.2014.09.020 Medline

39. F. Chen, R. K. Madduri, A. A. Rodriguez, B. F. Darst, A. Chou, X. Sheng, A. Wang, J. Shen, E. J. Saunders, S. K. Rhie, J. T. Bensen, S. A. Ingles, R. A. Kittles, S. S. Strom, B. A. Rybicki, B. Nemesure, W. B. Isaacs, J. L. Stanford, W. Zheng, M. Sanderson, E. M. John, J. Y. Park, J. Xu, Y. Wang, S. I. Berndt, C. D. Huff, E. D. Yeboah, Y. Tettey, J. Lachance, W. Tang, C. T. Rentsch, K. Cho, B. H. Mcmahon, R. B. Biritwum, A. A. Adjei, E. Tay, A. Truelove, S. Niwa, T. A. Sellers, K. Yamoah, A. B. Murphy, D. C. Crawford, A. V. Patel, W. S. Bush, M. C. Aldrich, O. Cussenot, G. Petrovics, J. Cullen, C. M. Neslund-Dudas, M. C. Stern, Z. Kote-Jarai, K. Govindasami, M. B. Cook, A. P. Chokkalingam, A. W. Hsing, P. J. Goodman, T. J. Hoffmann, B. F. Drake, J. J. Hu, J. M. Keaton, J. N. Hellwege, P. E. Clark, M. Jalloh, S. M. Gueye, L. Niang, O. Ogunbiyi, M. O. Idowu, O. Popoola, A. O. Adebiyi, O. I. Aisuodionoe-Shadrach, H. O. Ajibola, M. A. Jamda, O. P. Oluwole, M. Nwegbu, B. Adusei, S. Mante, A. Darkwa-Abrahams, J. E. Mensah, H. Diop, S. K. Van Den Eeden, P. Blanchet, J. H. Fowke, G. Casey, A. J. Hennis, A. Lubwama, I. M. Thompson, R. Leach, D. F. Easton, M. H. Preuss, R. J. Loos, S. M. Gundell, P. Wan, J. L. Mohler, E. T. Fontham, G. J. Smith, J. A. Taylor, S. Srivastava, R. A. Eeles, J. D. Carpten, A. S. Kibel, L. Multigner, M.-É. Parent, F. Menegaux, G. Cancel-Tassin, E. A. Klein, C. Andrews, T. R. Rebbeck, L. Brureau, S. Ambs, T. L. Edwards, S. Watya, S. J. Chanock, J. S. Witte, W. J. Blot, J. Michael Gaziano, A. C. Justice, D. V. Conti, C. A. Haiman, Evidence of Novel Susceptibility Variants for Prostate Cancer and a Multiancestry Polygenic Risk Score Associated with Aggressive Disease in Men of African Ancestry. *Eur. Urol.* **84**, 13–23 (2023). doi:10.1016/j.eururo.2023.01.022 Medline

40. N. McCormick, N. Lu, C. Yokose, A. D. Joshi, S. Sheehy, L. Rosenberg, E. T. Warner, N. Dalbeth, T. R. Merriman, K. G. Saag, Y. Zhang, H. K. Choi, Racial and Sex Disparities in Gout Prevalence Among US Adults. *JAMA Netw. Open* **5**, e2226804 (2022). doi:10.1001/jamanetworkopen.2022.26804 Medline

41. O. Weissbrod, F. Hormozdiari, C. Benner, R. Cui, J. Ulirsch, S. Gazal, A. P. Schoech, B. van de Geijn, Y. Reshef, C. Márquez-Luna, L. O'Connor, M. Pirinen, H. K. Finucane, A. L. Price, Functionally informed fine-mapping and polygenic localization of complex trait heritability. *Nat. Genet.* **52**, 1355–1363 (2020). doi:10.1038/s41588-020-00735-5 Medline

42. S. Rao, Y. Yao, D. E. Bauer, Editing GWAS: Experimental approaches to dissect and exploit disease-associated genetic variation. *Genome Med.* **13**, 41 (2021). doi:10.1186/s13073-021-00857-3 Medline

43. S. B. Gabriel, S. F. Schaffner, H. Nguyen, J. M. Moore, J. Roy, B. Blumenstiel, J. Higgins, M. DeFelice, A. Lochner, M. Faggart, S. N. Liu-Cordero, C. Rotimi, A. Adeyemo, R. Cooper, R. Ward, E. S. Lander, M. J. Daly, D. Altshuler, The structure of haplotype blocks in the human genome. *Science* **296**, 2225–2229 (2002). doi:10.1126/science.1069424 Medline

44. D. E. Reich, M. Cargill, S. Bolk, J. Ireland, P. C. Sabeti, D. J. Richter, T. Lavery, R. Kouyoumjian, S. F. Farhadian, R. Ward, E. S. Lander, Linkage disequilibrium in the human genome. *Nature* **411**, 199–204 (2001). doi:10.1038/35075590 Medline

45. A. J. Griswold, K. Celis, P. L. Bussies, F. Rajabli, P. L. Whitehead, K. L. Hamilton-Nelson, G. W. Beecham, D. M. Dykxhoorn, K. Nuytemans, L. Wang, O. K. Gardner, D. A. Dorfsman, E. H. Bigio, M. M. Mesulam, S. Weintraub, C. Geula, M. Gearing, E. McGrath-Martinez, C. L. Dalgard, W. K. Scott, J. L. Haines, M. A. Pericak-Vance, J. I. Young, J. M. Vance, Increased APOE ε4 expression is associated with the difference in Alzheimer's disease risk from diverse ancestral backgrounds. *Alzheimers Dement.* **17**, 1179–1188 (2021). doi:10.1002/alz.12287 Medline

46. O. D. Parra, L. N. Kohler, L. Landes, A. A. Soto, D. Garcia, J. Mullins, P. Molina, E. Pereira, D. J. Spegman, L. Soltani, L. J. Mandarino, Biobanking in Latinos: Current status, principles for conduct, and contribution of a new biobank, El Banco por Salud, designed to improve the health of Latino patients of Mexican ancestry with type 2 diabetes. *BMJ Open Diabetes Res. Care* **10**, e002709 (2022). doi:10.1136/bmjdrc-2021-002709 Medline

47. N. Mulder, A. Abimiku, S. N. Adebamowo, J. de Vries, A. Matimba, P. Olowoyo, M. Ramsay, M. Skelton, D. J. Stein, H3Africa: Current perspectives. *Pharm. Genomics Pers. Med.* **11**, 59–66 (2018). doi:10.2147/PGPM.S141546 Medline

48. SAIGE-GPU, A GPU version of SAIGE for full GRM GWAS analysis. https://github.com/exascale-genomics/SAIGE-GPU.

49. SAIGE-GPU, A GPU version of SAIGE for full GRM GWAS analysis. https://zenodo.org/records/10395632.

50. B. K. Bulik-Sullivan, P.-R. Loh, H. K. Finucane, S. Ripke, J. Yang, N. Patterson, M. J. Daly, A. L. Price, B. M. Neale; Schizophrenia Working Group of the Psychiatric Genomics Consortium, LD Score regression distinguishes confounding from polygenicity in genome-wide association studies. *Nat. Genet.* **47**, 291–295 (2015). doi:10.1038/ng.3211 Medline

51. C. C. Chang, C. C. Chow, L. C. Tellier, S. Vattikuti, S. M. Purcell, J. J. Lee, Second-generation PLINK: Rising to the challenge of larger and richer datasets. *Gigascience* **4**, 7 (2015). doi:10.1186/s13742-015-0047-8 Medline

52. K. Watanabe, E. Taskesen, A. van Bochoven, D. Posthuma, Functional mapping and annotation of genetic associations with FUMA. *Nat. Commun.* **8**, 1826 (2017). doi:10.1038/s41467-017-01261-5 Medline

53. R. Mägi, A. P. Morris, GWAMA: Software for genome-wide association meta-analysis. *BMC Bioinformatics* **11**, 288 (2010). doi:10.1186/1471-2105-11-288 Medline

54. W. McLaren, L. Gil, S. E. Hunt, H. S. Riat, G. R. S. Ritchie, A. Thormann, P. Flicek, F. Cunningham, The Ensembl Variant Effect Predictor. *Genome Biol.* **17**, 122 (2016). doi:10.1186/s13059-016-0974-4 Medline

55. S. Dong, N. Zhao, E. Spragins, M. S. Kagda, M. Li, P. Assis, O. Jolanki, Y. Luo, J. M. Cherry, A. P. Boyle, B. C. Hitz, Annotating and prioritizing human non-coding variants with RegulomeDB. bioRxiv 2022.10.18.512627 [Preprint] (2022); doi: doi:10.1101/2022.10.18.512627.

56. M. Kanehisa, S. Goto, KEGG: Kyoto encyclopedia of genes and genomes. *Nucleic Acids Res.* **28**, 27–30 (2000). doi:10.1093/nar/28.1.27 Medline

57. H. Hunter-Zinck, Y. Shi, M. Li, B. R. Gorman, S.-G. Ji, N. Sun, T. Webster, A. Liem, P. Hsieh, P. Devineni, P. Karnam, X. Gong, L. Radhakrishnan, J. Schmidt, T. L. Assimes, J. Huang, C. Pan, D. Humphries, M. Brophy, J. Moser, S. Muralidhar, G. D. Huang, R. Przygodzki, J. Concato, J. M. Gaziano, J. Gelernter, C. J. O'Donnell, E. R. Hauser, H. Zhao, T. J. O'Leary, P. S. Tsao, S. Pyarajan, VA Million Veteran Program, Genotyping Array Design and Data Quality Control in the Million Veteran Program. *Am. J. Hum. Genet.* **106**, 535–548 (2020). doi:10.1016/j.ajhg.2020.03.004 Medline

58. Wellcome Sanger Institute, Sanger Imputation Service, https://imputation.sanger.ac.uk/?about=1#referencepanels.

59. 1000 Genomes Project Consortium, A. Auton, L. D. Brooks, R. M. Durbin, E. P. Garrison, H. M. Kang, J. O. Korbel, J. L. Marchini, S. McCarthy, G. A. McVean, G. R. Abecasis, A global reference for human genetic variation. *Nature* **526**, 68–74 (2015). doi:10.1038/nature15393 Medline

60. A. Price, EIGENSOFT (Alkes Price's Faculty Website, Software); https://www.hsph.harvard.edu/alkes-price/software/.

61. H. Fang, Q. Hui, J. Lynch, J. Honerlaw, T. L. Assimes, J. Huang, M. Vujkovic, S. M. Damrauer, S. Pyarajan, J. M. Gaziano, S. L. DuVall, C. J. O'Donnell, K. Cho, K.-M. Chang, P. W. F. Wilson, P. S. Tsao, Y. V. Sun, H. Tang, J. M. Gaziano, R. Ramoni, J. Breeling, K.-M. Chang, G. Huang, S. Muralidhar, C. J. O'Donnell, P. S. Tsao, S. Muralidhar, J. Moser, S. B. Whitbourne, J. V. Brewer, J. Concato, S. Warren, D. P. Argyres, B. Stephens, M. T. Brophy, D. E. Humphries, N. Do, S. Shayan, X.-M. T. Nguyen, S. Pyarajan, K. Cho, E. Hauser, Y. Sun, H. Zhao, P. Wilson, R. McArdle, L. Dellitalia, J. Harley, J. Whittle, J. Beckham, J. Wells, S. Gutierrez, G. Gibson, L. Kaminsky, G. Villareal, S. Kinlay, J. Xu, M. Hamner, K. S. Haddock, S. Bhushan, P. Iruvanti, M. Godschalk, Z. Ballas, M. Buford, S. Mastorides, J. Klein, N. Ratcliffe, H. Florez, A. Swann, M. Murdoch, P. Sriram, S. S. Yeh, R. Washburn, D. Jhala, S. Aguayo, D. Cohen, S. Sharma, J. Callaghan, K. A. Oursler, M. Whooley, S. Ahuja, A. Gutierrez, R. Schifman, J. Greco, M. Rauchman, R. Servatius, M. Oehlert, A. Wallbom, R. Fernando, T. Morgan, T. Stapley, S. Sherman, G. Anderson, E. Sonel, E. Boyko, L. Meyer, S. Gupta, J. Fayad, A. Hung, J. Lichy, R. Hurley, B. Robey, R. Striker, VA Million Veteran Program, Harmonizing Genetic Ancestry and Self-identified Race/Ethnicity in Genome-wide Association Studies. *Am. J. Hum. Genet.* **105**, 763–772 (2019). doi:10.1016/j.ajhg.2019.08.012 Medline

62. L. Bastarache, Using Phecodes for Research with the Electronic Health Record: From PheWAS to PheRS. *Annu. Rev. Biomed. Data Sci.* **4**, 1–19 (2021). doi:10.1146/annurev-biodatasci-122320-112352 Medline

63. A. Verma, Y. Bradford, S. Dudek, A. M. Lucas, S. S. Verma, S. A. Pendergrass, M. D. Ritchie, A simulation study investigating power estimates in phenome-wide association studies. *BMC Bioinformatics* **19**, 120 (2018). doi:10.1186/s12859-018-2135-0 Medline

64. X. T. Nguyen, S. B. Whitbourne, Y. Li, R. M. Quaden, R. J. Song, H. A. Nguyen, K. Harrington, L. Djousse, J. V. V. Brewer, J. Deen, S. Muralidhar, R. B. Ramoni, K. Cho, J. P. Casas, P. S. Tsao, J. M. Gaziano, S. Muralidhar, J. Moser, J. E. Deen, J. Michael Gaziano, S. Muralidhar, J. Beckham, K.-M. Chang, P. S. Tsao, S.-W. Luoh, J. P. Casas, J. Michael Gaziano, P. S. Tsao, J. P. Casas, L. Churby, S. B. Whitbourne, J. V. Brewer, M. T. Brophy, L. E. Selva, S. A. Shayan, K. Cho, S. Pyarajan, P. S. Tsao, K. Cho, S. L. DuVall, T. Connor, D. P. Argyres, P. S. Tsao, J. M. Gaziano, B. Stephens, P. Wilson, R. McArdle, L. Dellitalia, K. Mattocks, J. Harley, J. Whittle, F. Jacono, J. Beckham, J. Wells, S. Gutierrez, K. Alexander, K. Hammer, J. Norton, G. Villareal, S. Kinlay, J. Xu, M. Hamner, R. Mathew, S. Bhushan, P. Iruvanti, M. Godschalk, Z. Ballas, R. Smith, S. Mastorides, J. Moorman, S. Gappy, J. Klein, N. Ratcliffe, A. Palacio, O. Okusaga, M. Murdoch, P. Sriram, S. S. Yeh, N. Tandon, D. Jhala, S. Aguayo, D. Cohen, S. Sharma, S. Liangpunsakul, K. A. Oursler, M. Whooley, S. Ahuja, J. Constans, P. Meyer, J. Greco, M. Rauchman, R. Servatius, M. Gaddy, A. Wallbom, T. Morgan, T. Stapley, P. Liang, D. Fujii, P. Tsao, P. Strollo, E. Boyko, J. Walsh, S. Gupta, M. Huq, J. Fayad, A. Hung, J. Lichy, R. Hurley, B. Robey, P. Balasubramanian, VA Million Veteran Program, Data Resource Profile: Self-reported data in the Million Veteran Program: survey development and insights from the first 850 736 participants. *Int. J. Epidemiol.* **52**, e1–e17 (2023). doi:10.1093/ije/dyac133 Medline

65. T. W. Winkler, F. R. Day, D. C. Croteau-Chonka, A. R. Wood, A. E. Locke, R. Mägi, T. Ferreira, T. Fall, M. Graff, A. E. Justice, J. Luan, S. Gustafsson, J. C. Randall, S. Vedantam, T. Workalemahu, T. O. Kilpeläinen, A. Scherag, T. Esko, Z. Kutalik, I. M. Heid, R. J. F. Loos; Genetic Investigation of Anthropometric Traits (GIANT) Consortium, Quality control and conduct of genome-wide association meta-analyses. *Nat. Protoc.* **9**, 1192–1212 (2014). doi:10.1038/nprot.2014.071 Medline

66. Y. Deng, W. Pan, Improved Use of Small Reference Panels for Conditional and Joint Analysis with GWAS Summary Statistics. *Genetics* **209**, 401–408 (2018). doi:10.1534/genetics.118.300813 Medline

67. R. E. Peterson, K. Kuchenbaecker, R. K. Walters, C.-Y. Chen, A. B. Popejoy, S. Periyasamy, M. Lam, C. Iyegbe, R. J. Strawbridge, L. Brick, C. E. Carey, A. R. Martin, J. L. Meyers, J. Su, J. Chen, A. C. Edwards, A. Kalungi, N. Koen, L. Majara, E. Schwarz, J. W. Smoller, E. A. Stahl, P. F. Sullivan, E. Vassos, B. Mowry, M. L. Prieto, A. Cuellar-Barboza, T. B. Bigdeli, H. J. Edenberg, H. Huang, L. E. Duncan, Genome-wide Association Studies in Ancestrally Diverse Populations: Opportunities, Methods, Pitfalls, and Recommendations. *Cell* **179**, 589–603 (2019). doi:10.1016/j.cell.2019.08.051 Medline

68. HapMAP3 SNP list for LDSC Heritability and Genetic Correlations; https://console.cloud.google.com/storage/browser/_details/broad-alkesgroup-public-requester-pays/LDSCORE/w_hm3.snplist.bz2;tab=live_object.

69. C. Wallace, C. Giambartolomei, V. Plagnol, coloc: Colocalisation Tests of Two Genetic Traits. R package version 5.2.2 (2023); https://cran.r-project.org/web/packages/coloc/index.html

70. C. J. Willer, Y. Li, G. R. Abecasis, METAL: Fast and efficient meta-analysis of genomewide association scans. *Bioinformatics* **26**, 2190–2191 (2010). doi:10.1093/bioinformatics/btq340 Medline

71. O. Weissbrod, F. Hormozdiari, C. Benner, R. Cui, J. Ulirsch, S. Gazal, A. P. Schoech, B. van de Geijn, Y. Reshef, C. Márquez-Luna, L. O'Connor, M. Pirinen, H. K. Finucane, A. L. Price, Functionally informed fine-mapping and polygenic localization of complex trait heritability. *Nat. Genet.* **52**, 1355–1363 (2020). doi:10.1038/s41588-020-00735-5 Medline

72. The Schizophrenia Workgroup of Psychiatric Genomics Consortium, K. Yuan, R. J. Longchamps, A. F. Pardiñas, M. Yu, T.-T. Chen, S.-C. Lin, Y. Chen, M. Lam, R. Liu, Y. Xia, Z. Guo, W. Shi, C. Shen, M. J. Daly, B. Neale, Y.-C. A. Feng, Y.-F. Lin, C.-Y. Chen, M. O'Donovan, T. Ge, H. Huang, Fine-mapping across diverse ancestries drives the discovery of putative causal variants underlying human complex traits and diseases. medRxiv 2023.01.07.23284293 [Preprint] (2023); doi:10.1101/2023.01.07.23284293.

73. M.-H. Chen, L. M. Raffield, A. Mousas, S. Sakaue, J. E. Huffman, A. Moscati, B. Trivedi, T. Jiang, P. Akbari, D. Vuckovic, E. L. Bao, X. Zhong, R. Manansala, V. Laplante, M. Chen, K. S. Lo, H. Qian, C. A. Lareau, M. Beaudoin, K. A. Hunt, M. Akiyama, T. M. Bartz, Y. Ben-Shlomo, A. Beswick, J. Bork-Jensen, E. P. Bottinger, J. A. Brody, F. J. A. van Rooij, K. Chitrala, K. Cho, H. Choquet, A. Correa, J. Danesh, E. Di Angelantonio, N. Dimou, J. Ding, P. Elliott, T. Esko, M. K. Evans, J. S. Floyd, L. Broer, N. Grarup, M. H. Guo, A. Greinacher, J. Haessler, T. Hansen, J. M. M. Howson, Q. Q. Huang, W. Huang, E. Jorgenson, T. Kacprowski, M. Kähönen, Y. Kamatani, M. Kanai, S. Karthikeyan, F. Koskeridis, L. A. Lange, T. Lehtimäki, M. M. Lerch, A. Linneberg, Y. Liu, L.-P. Lyytikäinen, A. Manichaikul, H. C. Martin, K. Matsuda, K. L. Mohlke, N. Mononen, Y. Murakami, G. N. Nadkarni, M. Nauck, K. Nikus, W. H. Ouwehand, N. Pankratz, O. Pedersen, M. Preuss, B. M. Psaty, O. T. Raitakari, D. J. Roberts, S. S. Rich, B. A. T. Rodriguez, J. D. Rosen, J. I. Rotter, P. Schubert, C. N. Spracklen, P. Surendran, H. Tang, J.-C. Tardif, R. C. Trembath, M. Ghanbari, U. Völker, H. Völzke, N. A. Watkins, A. B. Zonderman, P. W. F. Wilson, Y. Li, A. S. Butterworth, J.-F. Gauchat, C. W. K. Chiang, B. Li, R. J. F. Loos, W. J. Astle, E. Evangelou, D. A. van Heel, V. G. Sankaran, Y. Okada, N. Soranzo, A. D. Johnson, A. P. Reiner, P. L. Auer, G. Lettre, VA Million Veteran Program, Trans-ethnic and Ancestry-Specific Blood-Cell Genetics in 746,667 Individuals from 5 Global Populations. *Cell* **182**, 1198–1213.e14 (2020). doi:10.1016/j.cell.2020.06.045 Medline

74. S. Haider, D. Waggott, E. Lalonde, C. Fung, F.-F. Liu, P. C. Boutros, A bedr way of genomic interval processing. *Source Code Biol. Med.* **11**, 14 (2016). doi:10.1186/s13029-016-0059-5 Medline

75. A. Frankish, M. Diekhans, I. Jungreis, J. Lagarde, J. E. Loveland, J. M. Mudge, C. Sisu, J. C. Wright, J. Armstrong, I. Barnes, A. Berry, A. Bignell, C. Boix, S. Carbonell Sala, F. Cunningham, T. Di Domenico, S. Donaldson, I. T. Fiddes, C. García Girón, J. M. Gonzalez, T. Grego, M. Hardy, T. Hourlier, K. L. Howe, T. Hunt, O. G. Izuogu, R. Johnson, F. J. Martin, L. Martínez, S. Mohanan, P. Muir, F. C. P. Navarro, A. Parker, B. Pei, F. Pozo, F. C. Riera, M. Ruffier, B. M. Schmitt, E. Stapleton, M.-M. Suner, I. Sycheva, B. Uszczynska-Ratajczak, M. Y. Wolf, J. Xu, Y. T. Yang, A. Yates, D. Zerbino, Y. Zhang, J. S. Choudhary, M. Gerstein, R. Guigó, T. J. P. Hubbard, M. Kellis, B. Paten, M. L. Tress, P. Flicek, GENCODE 2021. *Nucleic Acids Res.* **49** (D1), D916–D923 (2021). doi:10.1093/nar/gkaa1087 Medline

76. J. Nasser, D. T. Bergman, C. P. Fulco, P. Guckelberger, B. R. Doughty, T. A. Patwardhan, T. R. Jones, T. H. Nguyen, J. C. Ulirsch, F. Lekschas, K. Mualim, H. M. Natri, E. M. Weeks, G. Munson, M. Kane, H. Y. Kang, A. Cui, J. P. Ray, T. M. Eisenhaure, R. L. Collins, K. Dey, H. Pfister, A. L. Price, C. B. Epstein, A. Kundaje, R. J. Xavier, M. J. Daly, H. Huang, H. K. Finucane, N. Hacohen, E. S. Lander, J. M. Engreitz, Genome-wide enhancer maps link risk variants to disease genes. *Nature* **593**, 238–243 (2021). doi:10.1038/s41586-021-03446-x Medline

77. ENGREITSLAB, Mapping the regulatory wiring of the genome to discover genetic mechanisms of heart development and disease. https://www.engreitzlab.org/.

78. G. Yu, L.-G. Wang, Y. Han, Q.-Y. He, clusterProfiler: An R package for comparing biological themes among gene clusters. *OMICS* **16**, 284–287 (2012). doi:10.1089/omi.2011.0118 Medline

79. M. Kanehisa, M. Furumichi, Y. Sato, M. Ishiguro-Watanabe, M. Tanabe, KEGG: Integrating viruses and cellular organisms. *Nucleic Acids Res.* **49**, D545–D551 (2021). doi:10.1093/nar/gkaa970 Medline

80. Y. Benjamini, Y. Hochberg, Controlling the False Discovery Rate: A Practical and Powerful Approach to Multiple Testing. *J. R. Stat. Soc. Series B Stat. Methodol.* **57**, 289–300 (1995). doi:10.1111/j.2517-6161.1995.tb02031.x

81. D. Smedley, S. Haider, B. Ballester, R. Holland, D. London, G. Thorisson, A. Kasprzyk, BioMart—Biological queries made easy. *BMC Genomics* **10**, 22 (2009). doi:10.1186/1471-2164-10-22 Medline

82. M. J. M. F. Reijnders, R. M. Waterhouse, Summary Visualizations of Gene Ontology Terms With GO-Figure! *Front. Bioinform.* **1**, 638255 (2021). doi:10.3389/fbinf.2021.638255 Medline

83. D. Lin, in *Proceedings of the International Conference on Machine Learning* (ICML, 1998), Vol. 98, pp. 6645–6649.

84. X. Wang, I.-E. Nogues, M. Liu, T. Chen, X. Xiong, C.-L. Bonzel, H. Zhang, C. Hong, K. Dahal, L. Costa, J. M. Gaziano, S. C. Kim, Y.-L. Ho, K. Cho, T. Cai, K. P. Liao, Differential Associations of Interleukin 6 Receptor Variant Across Genetic Ancestries and Implications for Targeted Therapies. medRxiv 2022.09.24.22280325 [Preprint] (2022). doi:10.1101/2021.08.29.21262792v1.
